# Control over multiple molecular states with directional changes driven by molecular recognition

**DOI:** 10.1038/s41467-018-03220-0

**Published:** 2018-02-26

**Authors:** Takehiro Hirao, Dong Sub Kim, Xiaodong Chi, Vincent M. Lynch, Kazuaki Ohara, Jung Su Park, Kentaro Yamaguchi, Jonathan L. Sessler

**Affiliations:** 1 Department of Chemistry, The University of Texas at Austin, 105 East 24th Street-Stop A5300, Austin, Texas 78712-1224 USA; 2 Faculty of Pharmaceutical Sciences at Kagawa Campus, Tokushima Bunri University, 1314-1 Shido, Sanuki-city, Kagawa 769-2193 Japan; 3 Department of Chemistry, Sookmyung Women’s University, Cheongpa-ro 47-gil, Yongsan-gu, Seoul South Korea; 4 Department of Chemistry, Center for Supramolecular Chemistry and Catalysis, Shanghai University, Shanghai, 200444 China

## Abstract

Recently, ligand–metal coordination, stimuli-responsive covalent bonds, and mechanically interlinked molecular constructs have been used to create systems with a large number of accessible structural states. However, accessing a multiplicity of states in sequence from more than one direction and doing so without the need for external energetic inputs remain as unmet challenges, as does the use of relatively weak noncovalent interactions to stabilize the underlying forms. Here we report a system based on a bispyridine-substituted calix[4]pyrrole that allows access to six different discrete states with directional control via the combined use of metal-based self-assembly and molecular recognition. Switching can be induced by the selective addition or removal of appropriately chosen ionic guests. No light or redox changes are required. The tunable nature of the system has been established through a combination of spectroscopic techniques and single crystal X-ray diffraction analyses. The findings illustrate a new approach to creating information-rich functional materials.

## Abstract (web summary)

Multistate molecular systems usually rely on external energy inputs to switch between states. Here, the authors show that a bispyridyl calixpyrrole system directed by only weak noncovalent interactions and metal coordination can access six discrete structural states, with directional and sequential control.

## Introduction

Structurally well-defined supramolecular architectures have attracted considerable recent attention, both for their aesthetic appeal and for the new chemistry they enable. Metal cation coordination has had a central role in the blossoming of this fast-evolving field. The relatively greater strength of ligand–metal coordination bonds compared with other noncovalent interactions has permitted the stabilization of a range of elegant structures, including many with properties that are not mimicked in the case of less complex architectures^1–6^. An exciting new direction in the metal-based self-assembly field involves the use of metal–organic complexes to access multiple discrete states. Several systems are now known that allow switching between discrete states, such as open one-dimensional polymeric assemblies, closed two-dimensional rings, and three-dimensional capsule-like assemblies, via application of an external stimulus^7–15^. However, for the most part only monomer to closed, monomer to open, and closed to closed structural conversions have been achieved using this approach. Accessing > 2 discrete states in sequence from more than one direction has also proved challenging. In contrast, stimuli-responsive covalent bonds and mechanically interlinked molecular constructs have been used to effect reversible state changes and do so with control over directionality^16–26^. However, here energetic inputs, such as light, coupled chemical reactions, or redox changes are required to drive the systems in question.

**Fig. 1:**
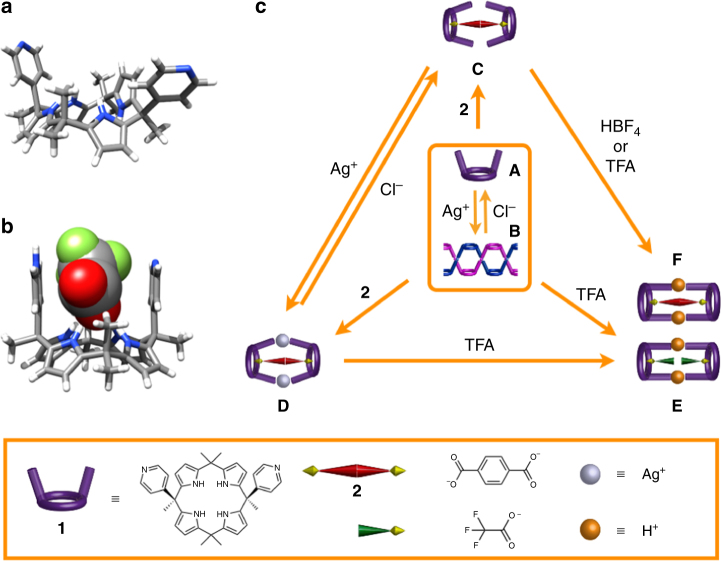
Crystal structures and graphical summary of the underlying equilibria. **a** Single crystal X-ray structures of bispyridine-substituted calix[4]pyrrole **1**. **b** Single crystal structure of trifluoroacetate-bound **1**. **c** Schematic representation of single crystal X-ray structures of the six different equilibrium states that can be accessed by judicious addition of ionic guests

Calix[4]pyrroles are non-aromatic tetrapyrrolic macrocycles that have been extensively studied as anion, ion-pair receptors, molecular containers, and, more recently, transmembrane transporters and building blocks for self-assembly^27–30^. They are readily accessible by simple acid-catalyzed condensation reactions involving (di)pyrrolic precursors and ketones. This has permitted a wide range of functionalized calix[4]pyrroles to be prepared^31–42^. When binding a Lewis basic anion, the calix[4]pyrrole ring undergoes a conformational “flip” from the so-called 1,3-alternate to the corresponding cone conformation^27,31,41^. During the conformational flip, substituents on the *meso* bridges undergo wing-like motion from an open to closed orientation (e.g., Fig. 1a,b).

We postulate that combining weak supramolecular interactions with ligand–metal coordination would allow access to multiple state changes with directional control provided via the simple addition of guest molecules. To test this hypothesis, we choose to work with the bispyridine-functionalized calix[4]pyrrole **1**^39,43–47^. In the case of the pyridine-functionalized calix[4]pyrrole **1**, this would have the effect of orientating the pyridyl nitrogen lone pairs in such a way that they might stabilize metal complexes with very different structures. Moreover, and in marked contrast to what is true for pure metal complexation-based systems, calix[4]pyrrole **1** would be expected to stabilize molecular ensembles that do not rely on metal coordination. Here we report that by using this mixed supramolecular–metal complexation approach it is possible to access six limiting states, namely a monomeric form **1**, an infinite coordination polymer, [**1**•Ag]_∞_, and four types of anion- and cation-bridged capsule dimers, and to do so under conditions of controlled equilibria. The states in question are readily accessible in sequence from two different directions and they have been fully characterized both in solution and in the solid state. Finally, introduction of chirality, namely the use of a non-racemic pyridine calix[4]pyrrole **3**, allows the system to operate off equilibrium in accord with the so-called “sergeant and soldiers” principle^48^.

## Results

### Studies of monomer **1**

Typical for a calix[4]pyrrole, receptor **1** in its anion bound cone conformation is characterized by a concave, bowl-like cavity into which charge diffuse cations, such as the cesium cation or imidazolium cations, can bind. However, unlike simple calix[4]pyrroles, the pyridine-functionalized system **1** contains putative metal complexation sites in two of its *meso*-positions. In the absence of a bound anion, these two potential donor sites are poorly preorganized (Fig. 1a). However, upon anion binding, the two pyridyl subunits present in **1** align in a parallel manner such that the two pyridine-derived nitrogen lone pairs point the same direction (Fig. 1b). As noted above and detailed below, this coordination-induced preorganization of the pyridinyl subunits allows for the stabilization of complex metal cation-containing structures and their transformation to other forms.

### Formation of silver-bridged double helical C[4]Ps

**Fig. 2:**
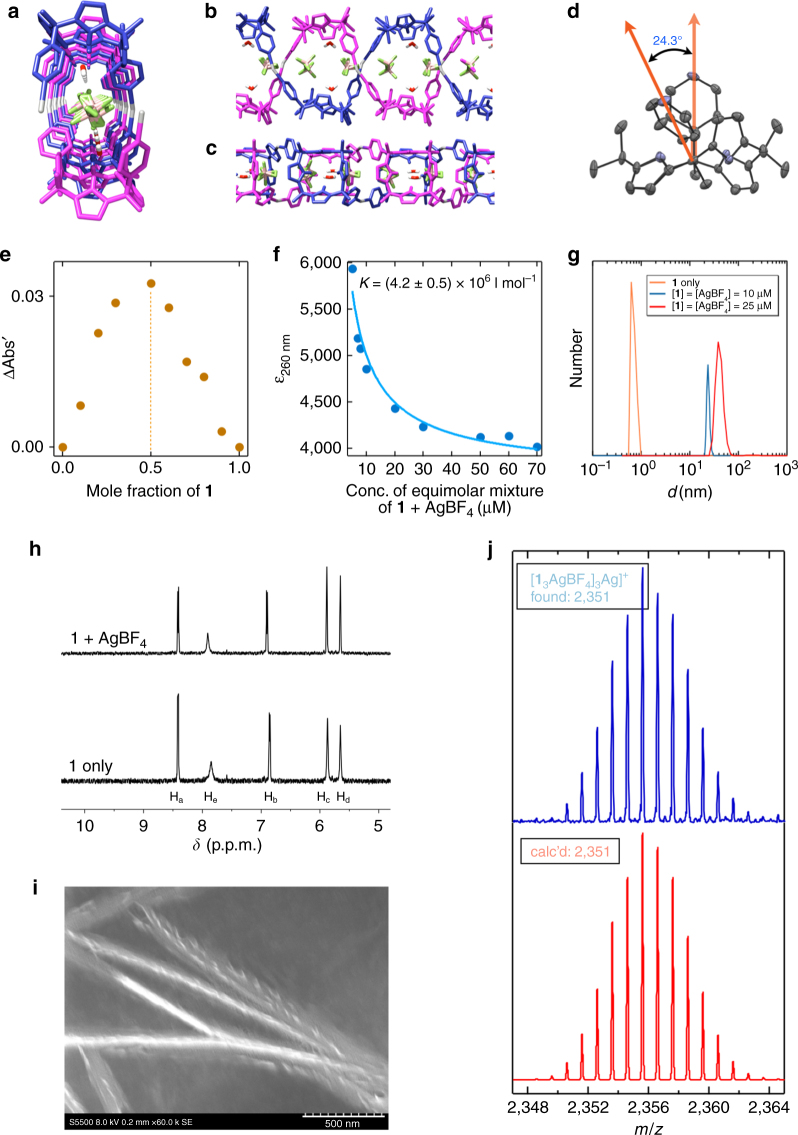
Studies of the Ag(I)-linked double-stranded helical complex. **a**–**c** Single-crystal X-ray structure of double helix comprised of **1**, AgBF_4_, and a water molecule viewed from the front **a**, the side **b**, and the top **c**. **d** Partial structure illustrating the torsional angle between the two pyridine subunits (protons are omitted for clarity). **e** Job-plot analysis of the components, **1** and AgBF_4_, which combine to produce a helix with net 1 : 1 binding stoichiometry. **f** Binding isotherm analysis corresponding to the formation of a double-stranded complex from Ag(I) and **1**. **g** Size comparison between **1** and the helix derived from it, as inferred from dynamic light scattering experiments. **h** Expanded ^1^H NMR spectra highlighting the minimal spectral changes induced when **1** is exposed to Ag(I). **i** Scanning electron microscopic image of the helix. The scale bar denotes 0.5 μm. **j** Cold spray ionization (CSI)–MS spectrum of the mixture of **1** + AgBF_4_. Shown here is a mass spectrum corresponding to the presumed trimeric complex, [**1**_3_(AgBF_4_)_3_Ag]^+^

Single crystals of **1** and its complexes were obtained from a 1 : 1 mixture of chloroform and acetonitrile (MeCN) in the presence and absence of guest molecules by allowing the solvents to undergo slow evaporation. X-ray diffraction analysis of single crystals obtained from a mixture of **1** and AgBF_4_ revealed a double-stranded helical chain linked by Ag(I) cations that contains both water molecules and BF_4_^−^ anions inside the helix (Fig. 2a–c). This corresponds to Form B in Fig. 1. The Ag(I) center is constrained in a bent T-shaped coordination geometry with two pyridines and a molecule of MeCN serving as the dominant ligands (Supplementary Fig. 18). The Pyr(N)**–**Ag**–**Pyr(N) angle and the torsion angle between the two pyridine moieties are 155.6° and 24.3°, respectively (Supplementary Fig. 18 and Fig. 2d). This torsion angle is thought to be important in terms of permitting formation of a helical structure. The BF_4_^−^ counter anions are not coordinated to the pyrrolic NH protons. As a result, the two pyridine substituents experience more conformational freedom than they would in the cone conformation. Specifically, they can twist to allow formation of a metal-linked spiral structure. This is reflected in the observed torsion angle (Fig. 2d).

Scanning electron microscopy (SEM) measurements provided support for the proposed formation of helical polymeric assemblies in the solid state upon mixing **1** and AgBF_4_ in THF solution, and allowing the mixture to evaporate to dryness (Fig. 2i and Supplementary Fig. 19). Widespread twisted wire-like shapes are seen in the SEM, which may reflect propagation to larger length scales than the monomeric helical structures seen in the single-crystal structure discussed above. The ensembles were found to be 141 ± 30 nm wide. Judging from the crystal structure, the width of each double strand is 1.7 nm. On this basis, it is inferred that the assemblies studied by SEM consist of bundles of ~ 80 polymeric chains.

Insights into the binding ratio came from a Job’s plot (Fig. 2e), which proved consistent with a 1 : 1 binding stoichiometry between **1** and the Ag(I) cation. Nevertheless, the actual absorption features of a 1 : 1 mixture of **1** and AgBF_4_ were found to be concentration dependent. When the total concentration of a 1 : 1 mixture of **1** and AgBF_4_ was increased (from 5.0 × 10^−6^ mol l^−1^ to 7.0 × 10^−5^ mol l^−1^ at 24 °C), a new band was seen to grow in around 300 nm (Supplementary Fig. 20). A plot of the extinction coefficients of the mixture at 260 nm vs. concentration yielded a hyperbolic curve. Assuming an isodesmic binding model, an affinity constant, *K*_ag_, of (4.2 ± 0.5) × 10^6^ l mol^−1^ could be calculated via nonlinear curve fitting^35^ (Fig. 2f). The good fit observed provided additional support for the 1 : 1 empirical binding stoichiometry inferred from the Job’s plot.

The complexation behavior of **1** in solution was also assessed by means of proton nuclear magnetic resonance (^1^H NMR) spectroscopy, dynamic light scattering (DLS), and cold spray ionization mass spectrometry (CSI–MS)^49^. When AgBF_4_ was added to a solution of **1** in MeCN-*d*_3_, the signals corresponding to the pyridine protons were found to undergo minimal shifts (Fig. 2h). Typically, only modest shifts are seen when Ag(I) cations are coordinated by pyridine ligands in MeCN solution^50^. Also found was a negligible change in the NH proton signals of **1**; this is as expected given the non-coordinating nature of the BF_4_^−^ counter anions. DLS measurements of pure THF solutions of **1** provided no evidence of species with a hydrodynamic diameter (*d*) > 1.0 nm. In contrast, when the hydrodynamic diameter (*d*) of an equimolar mixture of **1** and AgBF_4_ in THF was analyzed, concentration-dependent behavior was seen. For instance, at total concentrations of 2.0 × 10^−5^ and 5.0 × 10^−5^ mol l^−1^, *d* values of 11.7 and 37.8 nm, respectively, were recorded (Fig. 2g). Such findings are consistent with a conversion from short oligomers to long oligomers as the concentration increases. These higher order species are abbreviated as [**1**•Ag]_∞_, which corresponds to Form B of Fig. 1.

To complement the DLS studies, a mixture of **1** and AgBF_4_ was made up in a solution of acetonitrile/chloroform (1 : 1, v/v) and subject to CSI–MS analysis. The highest observable peak of appreciable intensity was seen at m/z = 2355.58 (Fig. 2j, Supplementary Fig. 21, and Supplementary Table 1). This corresponds to an oligomeric species with an empirical formula [**1**_3_•(AgBF_4_)_3_•Ag]^+^.

**Fig. 3:**
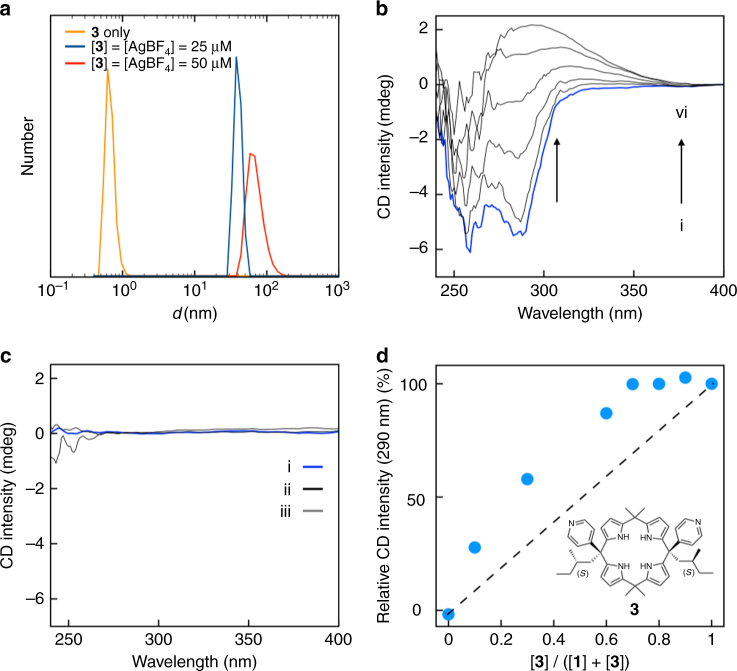
Operating off the racemic equilibrium state. **a** Size comparison between **3** and the helix derived from it, as inferred from dynamic light scattering experiments. **b** CD spectral changes of **3** (2.5 × 10^−4^ mol l^−1^) upon the addition of AgBF_4_. The concentrations of AgBF_4_ are (i–vi) 0.0, 0.5, 1.0, 1.5, 2.0, 2.5 × 10^−4^ mol l^−1^. **c** CD spectral changes of **1** (2.5 × 10^−4^ mol l^−1^) seen upon the addition of AgBF_4_. The concentrations of AgBF_4_ are (i–iii) 0.0, 1.3, 2.5 × 10^−4^ mol l^−1^. **d** Plots of the relative CD intensity measured at 290 nm vs mole fraction of **3**. At each datum point, the concentration of AgBF_4_ was equal to the total concentration of **1** + **3**. The dotted line indicates the expected CD intensity in the absence of chiral amplification. All studies were carried out in THF

Support for the notion that the present helix-forming system can be modified to operate off equilibrium came from DLS and circular dichroism (CD) measurements involving the non-racemic pyridine-containing calix[4]pyrrole **3** (Fig. 3 and Supplementary Figs 7–17). As in the case of **1**, the size of the construct produced from a 1 : 1 mixture of **3** and AgBF_4_ was found to depend on the total concentration of the two species as inferred from DLS measurements carried out in THF (Fig. 3a). In analogy to what is seen for **1**, at high total concentrations of **3** and AgBF_4_, the oligomeric species, [**3**•Ag]_∞_, dominates. This species is characterized by a CD band around 290 nm, which is dependent on the Ag(I) concentration, as would be expected for the formation of a non-racemic helix (Fig. 3b). In contrast, the achiral calix[4]pyrrole **1** fails to produce a discernible CD spectrum when treated with AgBF_4_ (Fig. 3c). On the other hand, adding a relatively small fraction of **3** to **1** led to formation of a CD signal that was greater than that expected on the basis of the concentration of **3** alone. In other words, adding **3** to **1** drives the system away from the racemic mixture that is produced in the absence of **3** (Fig. 3d and Supplementary Fig. 22). It thus operates off equilibrium in accord with the so-called “sergeant and soldiers” principle^48^.

### Helix to capsule interconversions

**Fig. 4:**
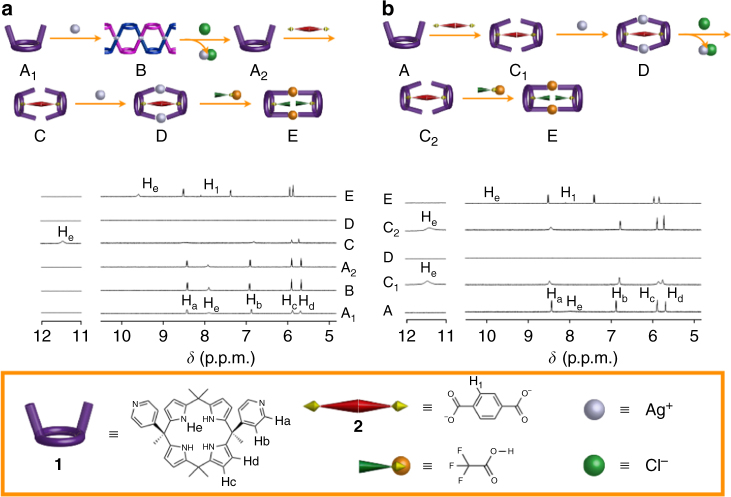
Schematic illustrations of the transformations between states. **a** Route 1 (A_1_ → B → A_2_ → C → D → E). **b** Route 2 (A → C_1_ → D → C_2_ → E). The corresponding ^1^H NMR spectral changes of **1** (2.0 × 10^−3^ mol l^−1^) were recorded in MeCN-*d*_3_

To probe the putative responsive nature of the species that could be accessed from **1**, tetrabutylammonium chloride (TBACl) was added to the solution of **1** and AgBF_4_ (giving B) discussed above (Fig. 4a). The results of this addition were changes in the proton NMR spectra recorded in both MeCN-*d*_3_ and THF-*d*_8_, and were consistent with reversible transformations between the helical (B) and monomeric (A) forms of **1** (Fig. 4a, spectra A_1_ to A_2_, and Supplementary Fig. 23). Concordant results were obtained in both solvents, although an unidentified impurity complicated the analyses in THF-*d*_8_. Changes in the DLS profiles were seen that support this conclusion (Supplementary Fig. 24).

To the solution obtained after TBACl addition (i.e., Form A), terephthalate anion (**2**) was added as its *bis-*tetrabutylammonium (bisTBA) salt. This addition triggered characteristic downfield shifts in the pyrrolic NH resonances (to ca. 11.5 p.p.m.) that are ascribed to hydrogen bonding interactions involving the pyrrolic protons and dianion **2** (Fig. 4a, spectrum C). Upon complexation, the aromatic proton resonances of dianion **2** broaden. This is rationalized in terms of **2** being encapsulated by a pair of calix[4]pyrroles **1** (Fig. 4a, spectrum C, and Supplementary Figs 23, 25–28). A separate UV-vis spectroscopic Job plot analysis in THF provided support for a 2 : 1 receptor : anion binding stoichiometry (Supplementary Fig. 29). This finding is consistent with what has been found previously in studies involving calix[4]pyrroles and ditopic guests^34,35,39,40^. We thus propose that under the conditions of the Job plot analysis, as well as when terephthalate anion **2** is added to a mixture of **1**, AgBF_4_, and TBACl, a molecular capsule is formed wherein guest **2** is bound in a clamshell fashion between two molecules of **1**. This corresponds to structure C in Fig. 1. However, Form C can be collapsed to a 1 : 1 anion-receptor complex via the further addition of **2**. Furthermore, the addition of 1.0 equivalent (as opposed to 0.5 equiv.) of **2** to a solution of **1** afforded 1 : 1 complex (Supplementary Fig. 30)^34^. From a nonlinear curve fitting of the changes in the UV-vis spectrum as **2** is titrated with **1** in THF, the binding constants, *K*_1_ and *K*_2_, corresponding to capsule formation were calculated to be 600 ± 100 and 9,000 ± 1,000  l mol^−1^, respectively (Supplementary Fig. 29).

**Fig. 5:**
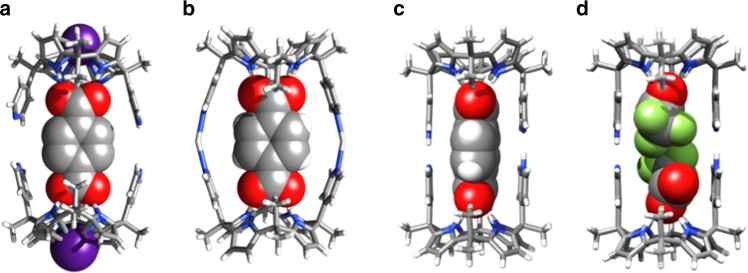
Crystal structures of the molecular capsules. **a** **1**_2_ ⊃ **2**•Cs^**+**^, which lacks a bridging cation (H^+^ or Ag^+^) between the pyridine arms. **b** **1**_2_ ⊃ **2**, wherein the pyridine moieties are bridged by Ag^+^ cations. **c** **1**_2_ ⊃ **2** and **d** **1**_2_ ⊃ TFA_2_, wherein the pyridine arms are connected via a bridging proton as inferred from the metric parameters and charge considerations

Structural evidence consistent with formation of a capsule in the solid state came from an X-ray diffraction analysis of a single crystal obtained from a mixture of **1**, CsOH, and terephthalic acid (Fig. 5a). In contrast to the infinite helical architecture (Form B), a well-defined, discrete molecular capsular structure (corresponding to Form C) is seen that is stabilized by eight hydrogen bonding interactions between the pyrrolic NH protons of **1** and dianion of **2** (Fig. 5a). The cone conformation of **1** within this capsule structure allows complexation of the Cs^+^ cation in the “bowl-like” cavity of the calix[4]pyrrole. This cation recognition may serve to stabilize the structure. The so-called T-shaped or edge-to-face interactions between the aromatic C-Hs of **2** and the π surface of the pyridines subunits present in **1** may also contribute to the stability of the capsule-like complex.

Addition of AgBF_4_ to the mixture of **1**, AgBF_4_, TBACl, and **2** in either MeCN-*d*_3_ or THF-*d*_8_ (Supplementary Fig. 23) leads to precipitation. Alhough not identified, this precipitate was presumed to consist of a mixture of AgCl and a silver-bridged capsule-like complex corresponding to Form D. This precipitation led to an absence of any discernable peaks in the ^1^H NMR spectrum (Fig. 4a, spectrum D). However, when AgBF_4_ was added to a mixture of **1** and **2** in a solution of MeCN and MeOH containing a trace of dimethyl sulfoxide (corresponding to the transformation A → C_1_ in Fig. 4b), diffraction-quality crystals were obtained along with considerable quantities of an amorphous powdery precipitate. Structural analysis of the crystals revealed that another capsule-like complex was formed corresponding to Form D in Figs 1 and 4b (Fig. 5b). In this case, two sets of Pyr(N)-Ag(I)-Pyr(N) bridges between the calix[4]pyrrole subunits were observed. Pyrrole NH-dianion hydrogen bond interactions analogous to those seen in the capsule-like structure (form C_1_) before the addition of AgBF_4_ were also observed, as inferred from the metric parameters.

When the above mixture was treated with 1 molar equivalent of trifluoroacetic acid (TFA) relative to **1**, protons, one per pair of pyridines, take the place of the bridging Ag(I) cations. The silver cations are released and bound in the form of silver terephthalate, which is found outside the capsule, as deduced from NMR spectroscopic studies. The trifluoroacetate anions that result from deprotonation of the TFA are bound by the calix[4]pyrrole NH protons. The net result is formation of a 2 : 2 complex corresponding to Form E in Fig. 1.

Evidence in support of the above stimulus-induced changes in structure came from NMR spectroscopic studies carried out in MeCN-*d*_3_. As can be seen from an inspection of Fig. 4a spectrum E, when TFA is added to a mixture of **1**, AgBF_4_, TBACl, and **2**, the pyrrole NH and pyridine aromatic protons of **1** experience upfield and downfield shifts, respectively. The equilibrium between host (**1**) and guest (**2**) is characterized by an exchange rate that is slow on the NMR time scale, as inferred from the observation of two sets of NH signals at most stoichiometric ratios (Supplementary Figs 32 and 33). In contrast, the equilibrium between **1** and trifluoroacetate anion is characterized by an exchange rate that is fast on the NMR time scale. An average proton chemical shift for the NH signals is thus observed as increasing quantities of TFA are added to **1** or to mixtures of **1** containing other anionic guests (e.g., **2**) (Supplementary Figs 34 and 35). Thus, the disappearance of the NH resonance at 11.5 p.p.m. and the emergence of a new signal around 9.5 p.p.m. when 1 equivalent of TFA per subunit of **1** is added to a mixture of **1**, AgBF_4_, TBACl, and **2** is considered consistent with the trifluoroacetate anion replacing the terephthalate dianion (**2**) within the cavity under these solution phase conditions (Fig. 4a, spectrum E, and Supplementary Figs 36 and 37). The formation of the relatively stable salt, silver terephthalate, probably helps drive the conversion toward the proton-bridged bis-trifluoroacetate capsule complex corresponding to Form E.

In spite of what was inferred from the solution phase studies, an X-ray diffraction analysis of single crystals obtained by slow evaporation of MeCN solutions containing **1**, **2**, and TFA (Fig. 5c,d and Supplementary Fig. 53) revealed the presence of both dianion **2** and trifluoroacetate anion within the proton-bridged capsule structure and in a ratio of ca. 1 : 13 (corresponding to Forms E and F in Fig. 1). We infer that Form F is more stable than Form E in the solid state, whereas the reverse is true in solution. This likely reflects differences in the solubility of the two forms. In contrast to the bis-Cs^+^ and Ag^+^-linked cage structures containing an internally bound dianion (**2**) discussed above (Fig. 5a,b), the metric parameters and orientation of the guest **2** in Form F are consistent with the presence of stabilizing cation (Pyr(N)-H)–π (terephthalate) interactions. In particular, the π surfaces of the pyridine (pyridinium) arms in receptor **1** and the guest **2** are aligned (Fig. 5c).

Presumably because of the competition between the terephthalate dianion and the trifluoroacetate anion, efforts to produce a terephthalate bound proton-bridged capsule structure (i.e., Form F; Fig. 1c) in solution via the direct addition of TFA to mixtures of **1** and **2** proved unsuccessful (Supplementary Figs 36 and 37). However, this form could be obtained by adding aqueous HBF_4_ to a 2 : 1 mixture of **1** and **2** in THF-*d*_8_ (Supplementary Figs 38–42). Here, HBF_4_ was used to protonate the pyridine nitrogen atoms, as the resulting conjugate base (BF_4_^−^) does not bind appreciably to **1** or other calix[4]pyrroles and is thus “benign.” The addition of HBF_4_ gives rise to a clean NMR spectrum corresponding to a species of relatively high symmetry. Downfield shifts in the pyridine signals of **1** were observed, as were shifts in the two β-pyrrolic proton signals (H_c_ and H_d_) consistent with the presence of a bound terephthalate anion **2** (cf. Supplementary Fig. 25), as would be expected in the case of Form F. In contrast to what is observed in THF-*d*_8_, in MeCN-*d*_3_, removal of **2** could be achieved by adding 1.0 equiv. of aqueous HBF_4_ to the solution containing From C (Supplementary Figs 43–46). The free form of anion **2** is presumably better stabilized by this latter, more polar solvent, resulting in its release from within the capsule.

### Capsule-to-capsule interconversions

To explore whether the interconversion between the six limiting forms shown in Fig. 1c could be effected in a different order, 0.5 equivalents of **2** were added to a solution of **1** in MeCN-*d*_3_ (Fig. 4b). This addition led to a downfield shift in the pyrrolic NH signals of **1** to 11.5 p.p.m. A slight upfield shift was seen for the H_b_ resonance. A broadening of the H_a_ signals for **1** was also observed (Fig. 4b, spectrum C_1_). Similar results were found in THF-*d*_8_ (Supplementary Fig. 47). These spectral changes are analogous to what was found upon the sequential addition of AgBF_4_, TBACl, and **2** to a solution of **1** in MeCN-*d*_3_, as noted above (Fig. 4a, spectrum C). This correspondence leads us to propose that an analogous capsule complex, namely Form C (referred to as C_1_ in the context of the present sequence) is being stabilized.

The above mixture in MeCN-*d*_3_ was then treated with AgBF_4_. As in the first series of experiments, precipitation occurred and no discernable resonances from **1** or **2** could be observed. The precipitate is believed to correspond to the Ag(I)-linked capsule complex of **2** (Form D). Addition of chloride anion (as TBACl) served to reverse the equilibrium and regenerate Form C (referred to as C_2_ in the context of the present sequence). As expected, the ^1^H NMR spectrum exactly matches that for Form C obtained via the first addition sequence (cf. Figure 4a, spectrum **C** and 4b, spectra **C**_**1**_ and **C**_**2**_).

Treatment of this terephthalate-containing capsule species (Form C) with TFA in MeCN-*d*_3_ produced the proton-linked, trifluoroacetate-bearing molecular capsular structure (Form E), as inferred from its spectral correspondence with what was produced originally (Fig. 4b, spectrum E).

To provide support for the modular and pathway-independent nature of the present six-state system, efforts were made to obtain the various forms directly. For example, it was found that Forms D and E could be accessed without passing through Form C via sequences involving either A → B → D or A → B → E (Supplementary Fig. 48). This leads us to suggest that the present multistate system can operate to produce its various forms under conditions that are interchangeable, orthogonal, and chemically programmable.

## Discussion

As detailed above, we have developed a system that relies in part on weak supramolecular interactions and which allows six different discrete states to be accessed with directional control both in solution and in the solid state. Each state can be obtained via the addition of simple chemical species that control the underlying equilibria. The six states of this study can be readily distinguished from one another by a variety of experimental techniques, including solid-state single crystal X-ray diffraction analysis, DLS, and ^1^H NMR spectroscopic techniques. Depending on the nature of the added guests, the same basic calix[4]pyrrole receptor **1** can exist in the form of an open double-stranded, metal-bridged helical chain and closed anion-bridged molecular capsules with different linkages, namely a proton or a silver(I) cation. Of particular note is that the system presented here displays “state-function” behavior in that each form can be obtained via more than one sequence as long as the final conditions are appropriately defined. However, it can also be made to operate off equilibrium via the addition of sub-stoichiometric quantities of the non-racemic analogue **3** to THF solutions of **1** and AgBF_4_. The present study is expected to set the stage for the creation of new information-rich supramolecular ensembles that do not require elaborate synthesis or recourse to external energy sources to manifest their basic switching functions.

## Methods

### Characterization

The synthesis and characterization of all compounds  are described in the Supplementary Information, which further contain the ^1^H NMR, ^13^C NMR, correlation spectroscopy, nuclear Överhauser effect spectroscopy, heteronuclear single quantum correlation spectroscopy, high-resolution electrospray ionization MS, and CSI–MS of the compounds discussed in this report.

### Determination of binding stoichiometry

A Job plot was constructed to provide support for the host–guest ratios underlying complexes **1**•AgBF_4_ and **1**•**2** (**2** as its bisTBA salt) in THF. Here, a series of solutions containing the two monomers in question were made up such that the sum total of the monomer concentrations remained constant (1.0 × 10^−4^ mol l^−1^). The mole fraction of **1** was varied from 0.0 to 1.0. The absorbance changes were recorded at 260 nm for **1**•AgBF_4_ and 258 nm for **1**•**2**.

### Determination of an affinity constant corresponding to the interaction between **1** and AgBF_4_

A hyperbolic curve was obtained by plotting the total concentrations of a 1 : 1 mixture of **1** and AgBF_4_ versus the extinction coefficients (*ε*). Curve fitting was carried out using the Origin program based on an isodesmic binding model. The fitting functions are given by equation (1), where *K*, *C*, *ε*1, and *ε*a denote the affinity constant, the total concentration of the compounds, the extinction coefficient of the monomers, and the extinction coefficient of the aggregate species, respectively.

1 $$\varepsilon (C) = \frac{{KC + 1-\sqrt {2KC + 1} }}{{K^2C^2}}(\varepsilon 1-\varepsilon a) + \varepsilon a$$

### Determination of association constants for 1 and 2

A titration was performed wherein the concentration of a THF solution of the bisTBA salt of **2** (4.0 × 10^−5^ mol l^−1^) was fixed, while the concentration of **1** was varied. During the course of the titration, UV-vis absorption spectral changes were recorded from 800 to 200 nm. The resulting spectra were analyzed by HypSpec program based on a 1 : 2 host–guest binding model.

### SEM measurements

A stock solution of a mixture of **1** (1.0 × 10^−5^ mol l^−1^) and AgBF_4_ (1.0 × 10^−5^ mol l^−1^) in THF was prepared. The stock solution was drop-cast on a silicon wafer. The films were dried under reduced pressure overnight. SEM measurements were carried out using a HITACHI HI-7700 system.

### CSI–MS measurements

An acetonitrile/chloroform (1:1, v/v) solution of a 1 : 1 mixture of **1** and AgBF_4_ was prepared. The solution was stirred at room temperature for 1 day. CSI–MS measurement was performed using a Fourier transform ion cyclotron resonance mass spectrometer (Apex-Qe 9.4 T, Bruker Daltonics, Inc., Billerica, MA).

### DLS measurements

High-performance liquid chromatography (HPLC)-grade THF solutions of **1**, **2**, **3**, and AgBF_4_ were prepared. The solutions were mixed as described above and placed in 10 mm cuvettes. DLS measurements were carried out using Malvern 4700 submicrometer particle analyzer system.

### Circular dichroism studies

HPLC grade THF solutions of **1**, **3**, a mixture of **1** and AgBF_4_, and a mixture of **3** and AgBF_4_ were prepared. Solutions (3 ml) containing **1** and **3** were placed in 10 mm cuvettes. Small aliquots (2–30 μl) a mixture of **1** and AgBF_4_, and a mixture of **3** and AgBF_4_ were added to the solutions of **1** and **3**, respectively. CD measurements were carried out using a Jasco J-815 spectropolarimeter.

### Data availability

The authors declare that the all data supporting the findings of this study are available within this article and Supplementary Information files, or are available from the authors upon reasonable request. X-ray crystallographic coordinates for structures reported in this study have been deposited at the Cambridge Crystallographic Data Centre (CCDC), under deposition numbers 1058558-1058561, 1558007, and 1583699. These data can be obtained free of charge from The Cambridge Crystallographic Data Centre via www.ccdc.cam.ac.uk/data_request/cif.

## Electronic supplementary material

Supplementary Information

Peer Review File

## References

[1] Fujita M (1998). Metal-directed self-assembly of two- and three-dimensional synthetic receptors. Chem. Soc. Rev..

[2] Caulder DL, Raymond KN (1999). Supermolecules by design. Acc. Chem. Res..

[3] Leininger S, Olenyuk B, Stang PJ (2000). Self-assembly of discrete cyclic nanostructures mediated by transition metals. Chem. Rev..

[4] Smulders MMJ, Riddell IA, Browne C, Nitschke JR (2013). Building on architectural principles for three-dimensional metallosupramolecular construction. Chem. Soc. Rev..

[5] Cook TR, Stang PJ (2015). Recent developments in the preparation and chemistry of metallacycles and metallacages via coordination. Chem. Rev..

[6] Fujita D (2016). Self-assembly of tetravalent Goldberg polyhedra from 144 small components. Nature.

[7] McConnell AJ, Wood CS, Neelakandan PP, Nitschke JR (2015). Stimuli-responsive metal–ligand assemblies. Chem. Rev..

[8] Riddell IA (2013). Five discrete multinuclear metal-organic assemblies from one ligand: deciphering the effects of different templates. J. Am. Chem. Soc..

[9] Riddell IA (2014). Cation- and anion-exchanges induce multiple distinct rearrangements within metallosupramolecular architectures. J. Am. Chem. Soc..

[10] Yan X (2014). Photoinduced transformations of stiff-stilbene-based discrete metallacycles to metallosupramolecular polymers. Proc. Natl Acad. Sci. USA.

[11] Zhu R, Lübben J, Dittrich B, Clever GH (2015). Stepwise halide-triggered double and triple catenation of self-assembled coordination cages. Angew. Chem. Int. Ed..

[12] Burke MJ, Nichol GS, Lusby PJ (2016). Orthogonal selection and fixing of coordination self-assembly pathways for robust metallo-oraganic ensemble construction. J. Am. Chem. Soc..

[13] Holub J, Vantomme G, Lehn JM (2016). Training a constitutional dynamic network for effector recognition: storage, recall, and erasing of information. J. Am. Chem. Soc..

[14] Xie TZ (2016). Controlled interconversion of superposed-bistriangle, octahedron, and cuboctahedron cages constructed using a single, terpyridinyl-based polyligand and Zn^2+^. J. Am. Chem. Soc..

[15] Rizzuto F, Nitschke JR (2017). Stereochemical plasticity modulates cooperative binding in a Co^II^_12_L_6_ cuboctahedron. Nat. Chem..

[16] Browne WR, Feringa BL (2006). Making molecular machines work. Nat. Nanotechnol..

[17] Kay ER, Leigh DA, Zerbetto F (2007). Synthetic molecular motors and mechanical machines. Angew. Chem. Int. Ed..

[18] Niess F, Duplan V, Sauvage JP (2014). Molecular muscle: from species in solution to materials and devices. Chem. Lett..

[19] Bruns CJ, Stoddart JF (2014). Rotaxane-based molecular muscles. Acc. Chem. Res..

[20] Erbas-Cakmak S, Leigh DA, McTernan CT, Nussbaumer AL (2015). Artificial molecular machines. Chem. Rev..

[21] Kassem S (2017). Artificial molecular motors. Chem. Soc. Rev..

[22] Muraoka T, Kinbara K, Kobayashi Y, Aida T (2003). Light-driven open–close motion of chiral molecular scissors. J. Am. Chem. Soc..

[23] Aoki D, Aibara G, Uchida S, Takata T (2017). A rational entry to cyclic polymers via selective cyclization by self-assembly and topology transformation of linear polymers. J. Am. Chem. Soc..

[24] Ube H, Yasuda Y, Sato H, Shionoya M (2017). Metal-centered azaphosphatriptycene gear with a photo- and thermally driven mechanical switching function based on coordination isomerism. Nat. Commun..

[25] Zhao D, van Leeuwen T, Cheng J, Feringa BL (2017). Dynamic control of chirality and self-assembly of double-stranded helicates with light. Nat. Chem..

[26] Doistau B (2017). Six states switching of redox-active molecular tweezers by three orthogonal stimuli. J. Am. Chem. Soc..

[27] Gale PA, Sessler JL, Král V, Lynch V (1996). Calix[4]pyrroles: old yet new anion-binding agents. J. Am. Chem. Soc..

[28] Kim SK, Sessler JL (2014). Calix[4]pyrrole-based ion pair receptor. Acc. Chem. Res..

[29] Ko SK (2014). Synthetic ion transporters can induce apoptosis by facilitating chloride anion transport into cells. Nat. Chem..

[30] Kim DS, Sessler JL (2015). Calix[4]pyrroles: versatile molecular containers with ion transport, recognition, and molecular switching functions. Chem. Soc. Rev..

[31] Nielsen KA (2004). Tetra-TTF calix[4]pyrrole: a rationally designed receptor for electron-deficient neutral guests. J. Am. Chem. Soc..

[32] Palacios MA, Nishiyabu R, Marquez M, Anzenbacher P (2007). Supramolecular chemistry approach to the design of a high-resolution sensor array for multianion detection in water. J. Am. Chem. Soc..

[33] Cuesta L (2007). Design and synthesis of polymetallic complexes based on *meso*-calix[4]pyrrole: platforms for multielectron chemistry. J. Am. Chem. Soc..

[34] Cafeo G, Kohnke FH, Valenti L, White AJP (2008). pH-controlled molecular switches and the substrate-directed self-assembly of molecular capsules with a calix[4]pyrrole derivative. Chem. Eur. J..

[35] Park JS (2011). Chemoresponsive alternating supramolecular copolymers created from heterocomplementary calix[4]pyrroles. Proc. Natl Acad. Sci. USA.

[36] Valderrey V, Escudero-Adán EC, Ballester P (2012). Polyatomic anion assistance in the assembly of [2]pseudorotaxanes. J. Am. Chem. Soc..

[37] Kim SK (2012). KF and CsF recognition and extraction by a calix[4]crown-5 strapped calix[4]pyrrole multitopic receptor. J. Am. Chem. Soc..

[38] Valderrey V, Escudero-Adán EC, Ballester P (2013). Highly cooperative binding of ion-pair dimers and ion quartets by a bis(calix[4]pyrrole) macrotricyclic receptor. Angew. Chem. Int. Ed..

[39] Kim. DS, Lynch VM, Park JS, Sessler JL (2013). Three distinct equilibrium states via self-assembly: simple access to a supramolecular ion-controlled NAND logic gate. J. Am. Chem. Soc..

[40] Osorio-Plames L, Espelt M, Pericàs MA, Ballester P (2014). Reversible photocontrolled disintegration of a dimeric tetraurea-calix[4]pyrrole capsule with all-trans appended azobenzene units. Chem. Sci..

[41] Kim DS (2015). Redox- and pH-responsive orthogonal supramolecular self-assembly: an ensemble displaying molecular switching. J. Am. Chem. Soc..

[42] Mulugeta E (2017). Recognition, sensing, and trapping of bicarbonate anions with a dicationic *meso-*bis(benzimidazolium) calix[4]pyrrole. Chem.

[43] Sokkalingam P, Kim DS, Hwang H, Sessler JL, Lee CH (2012). A dicationic calix[4]pyrrole derivative and its use for the selective recognition and displacement-based sensing of pyrophosphate. Chem. Sci..

[44] Kaur S, Hwang H, Lee JT, Lee CH (2013). Displacement-based, chromogenic calix[4]pyrrole–indicator complex for selective sensing of pyrophosphate anion. Tetrahedron Lett..

[45] Adriaenssens L (2014). Thermodynamic characterization of halide–π interactions in solution using “two-wall” aryl extended calix[4]pyrroles as model system. J. Am. Chem. Soc..

[46] Saha I, Lee JH, Kim TS, Lee CH (2015). Remarkably selective, non-linear allosteric regulation of anion binding by a tetracationic calix[4]pyrrole homodimer. Chem. Commun..

[47] Kiriyama N, Ebihara M, Udagawa T, Miyaji H (2016). Self-organization of dipyridylcalix[4]pyrrole into a supramolecular cage for dicarboxylates. RSC Adv..

[48] Green MM (1989). Macromolecular stereochemistry: the out-of-proportion influence of optically zctive comonomers on the conformational characteristics of polyisocyanates. The sergeants and soldiers experiment. J. Am. Chem. Soc..

[49] Yamaguchi K (2003). Cold-spray ionization mass spectrum: principle and applications. J. Mass Spectrom..

[50] Fukino T (2014). Manipulation of discrete nanostructures by selective modulation of noncovalent forces. Science.

