# Supplementary material for: Control over multiple molecular states with directional changes driven by molecular recognition
Source: Nat Commun. 2018 Feb 26;9:823. doi: 10.1038/s41467-018-03220-0 (PMC5827562; doi:10.1038/s41467-018-03220-0)
Supplement: Supplementary file 1 — Supplementary Information [file 41467_2018_3220_MOESM1_ESM.pdf]

Supplementary Methods: All reagents and solvents were commercial reagent grade and were used without further purification. ^{1}H and ^{13}C NMR spectra were recorded on either a Varian Mercury-400 spectrometer or a 600 MHz Varian DirectDrive spectrometer, and chemical shifts were reported on the delta scale in ppm relative to residual chloroform (δ = 7.26 and 77.0 for ^{1}H and ^{13}C, respectively), acetonitrile (MeCN) (δ = 1.94 and 1.32 for ^{1}H and ^{13}C, respectively) and tetrahydrofuran (THF) (δ = 3.58 and 67.2 for ^{1}H and ^{13}C, respectively). High Resolution ESI mass spectrometry was performed using an Ion Spec Fourier Transform mass spectrometer (9.4 T). UV-vis absorption spectra were recorded on a Shimadzu UV-2550 spectrometer at room temperature. Infrared spectra were recorded on a Perkin-Elmer 1600 spectrometer. Optical rotations were recorded on an Atago AP-300 automatic polarimeter at the sodium line (589.3 nm) in chloroform. CD spectra were measured using a Jasco J-815 spectropolarimeter. Dynamic light scattering (DLS) measurements were carried out using 200-mW polarized laser source Nd: YAG (λ = 532 nm). The polarized scattered light data was collected at 90° in a self-beating mode with a Hamamatsu R942/02 photomultiplier. The signals were analyzed using a Malvern 4700 submicrometer particle analyzer system. X-ray crystallographic analyses were carried out on either Rigaku AFC12 diffractometer equipped with Saturn 724+ CCD or Agilent Technologies Super Nova Dual Source diffractometer equipped with a μ-focused Cu Kα radiation source (λ = 1.5418 Å) with collimating mirror monochromators. CSI-MS measurements were performed using a Fourier transform ion cyclotron resonance mass spectrometer (FT-ICR MS; Apex-Qe 9.4 T, Bruker Daltonics, Inc. Billerica, MA). The heater of the desolvation assembly was turned off in the electrospray ionization source and the temperature was kept around at 310 K. General measurement conditions were as follows: Positive ion mode, capillary voltage, 4.5 kV; dry gas flow rate, 5.0 L/min; nebulizer gas flow rate, 1.0 L/min; and sample flow rate, 120 μL/h. Isotope patterns were calculated by using "Simulate Pattern" packaged in Bruker Daltonics software (Data Analysis, ver. 4.0). Preparative separations were performed by silica gel gravity column chromatography (Sorbent Technologies, Silica Gel 60, particle size 40-63 μm, pH range 6.0-8.0). Compound **1** was synthesized according to a reported method[1,2] (Supplementary Fig. 1). Although compound **8** was commercially available, it was synthesized from 4-acetylpyridine and (*S*)-1-chloro-2-methylbutane according to a reported method[3] (Supplementary Fig. 7).

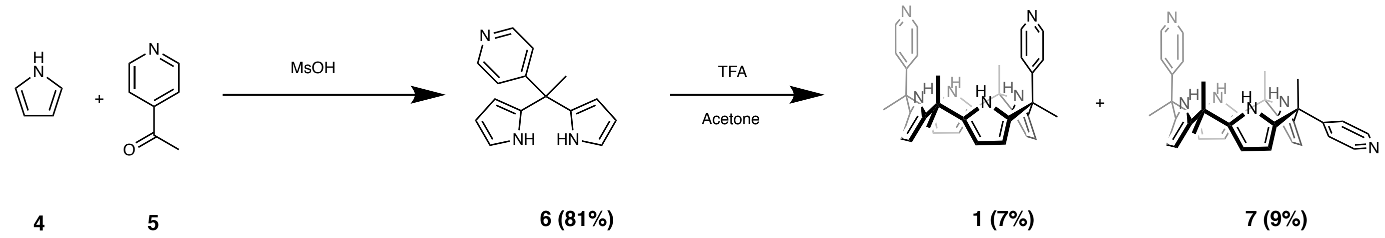

Supplementary Figure 1. Synthesis of **1**. Ms = methanesulfonyl; TFA = trifluoroacetic acid.

Supplementary Note 1. Compound data for **1.**

Compound **1**: M.p. 220 °C (decomp.); ^{1}H NMR (600 MHz, THF-d_8): δ 8.36 (dd, J_1 = 1.8 Hz, J_2 = 4.6 Hz, 4H), 8.05 (br, 4H), 6.86 (dd, J_1 = 1.8 Hz, J_2 = 4.6 Hz, 4H), 5.86 (m, 4H), 5.52 (m, 4H), 1.87 (s, 6H), 1.62 (s, 6H), 1.51 (s, 6H) ppm; ^{13}C NMR (125 MHz, THF-d_8): δ 158.0, 149.9, 139.6, 136.0, 123.1, 106.3, 103.5, 45.4, 35.8, 31.1, 27.0, 26.8 ppm; FTIR (neat): ν 2973, 1710, 1596, 1493, 1409, 1359, 1328, 1276, 1253, 1220, 1070, 1041, 1007, 825, 773, 710 cm^{-1}; HRMS (ESI$^+$) calcd. for $C_{36}H_{38}N_6$ *m/z* 555.3231 [M+H]$^+$, found *m/z* 555.3231.

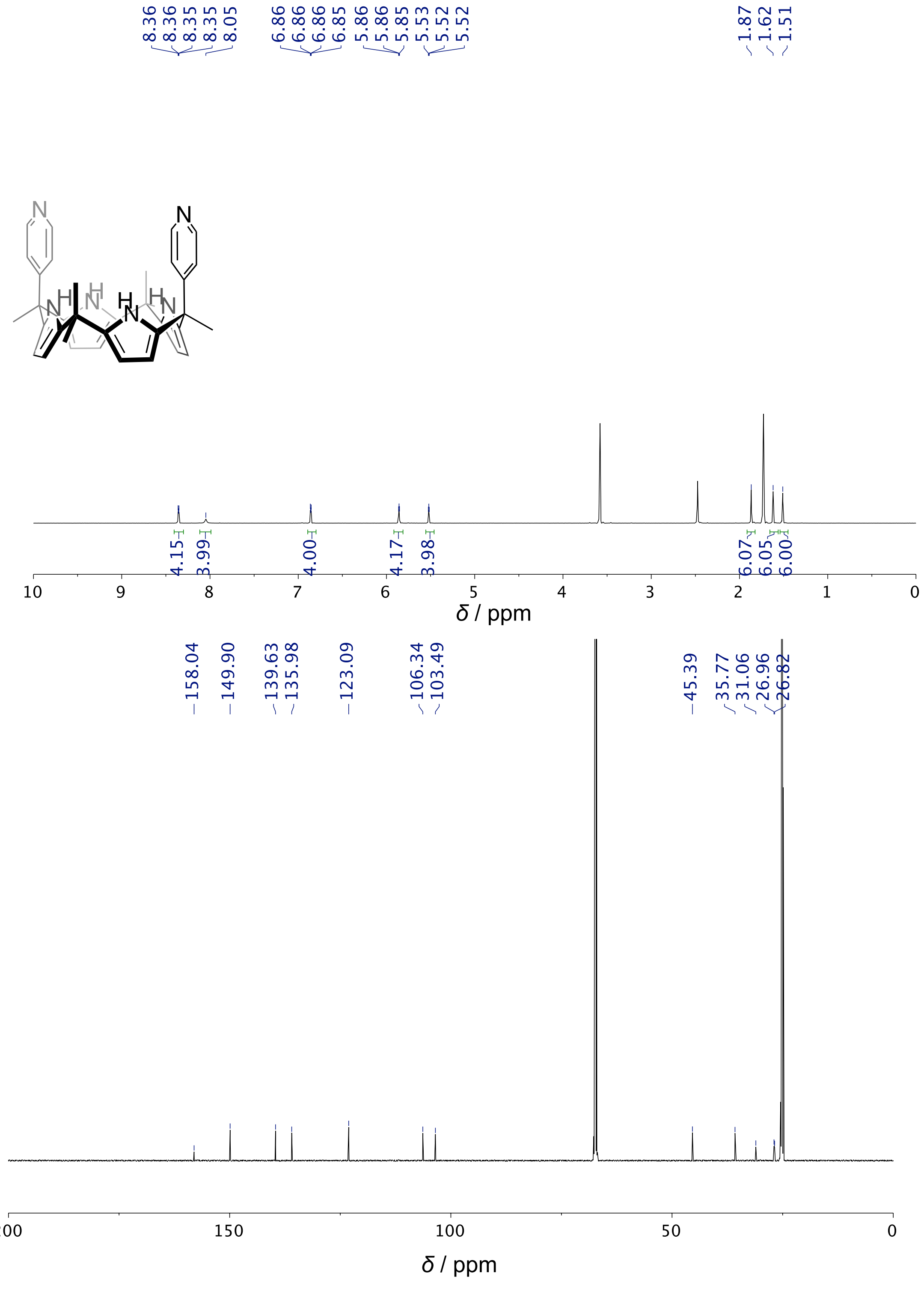

Supplementary Figure 2. ^{1}H and ^{13}C NMR spectra of **1** in THF-d_8.

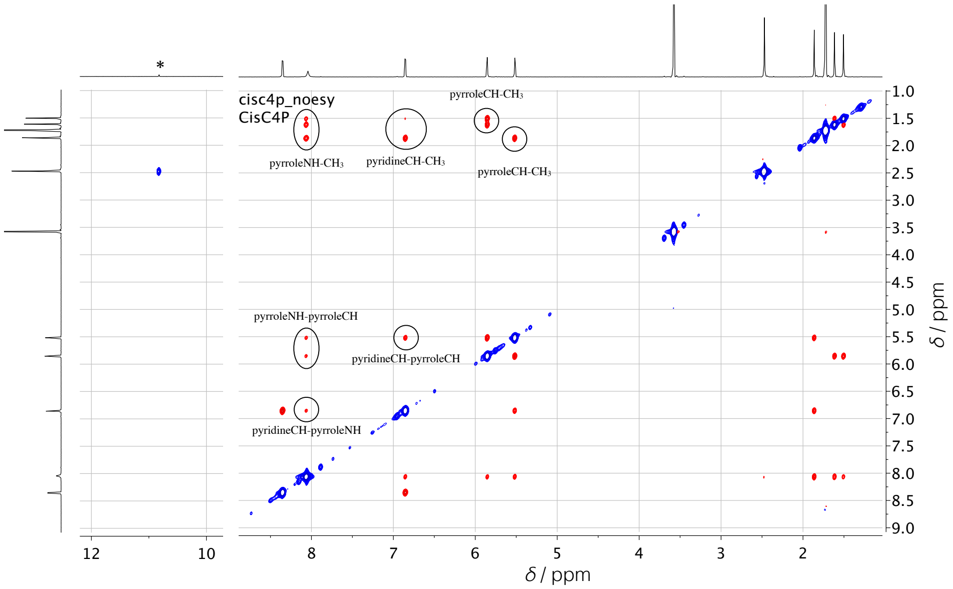

Supplementary Figure 3. NOESY spectrum of **1** in THF-d_8. *Indicates solvent impurity.

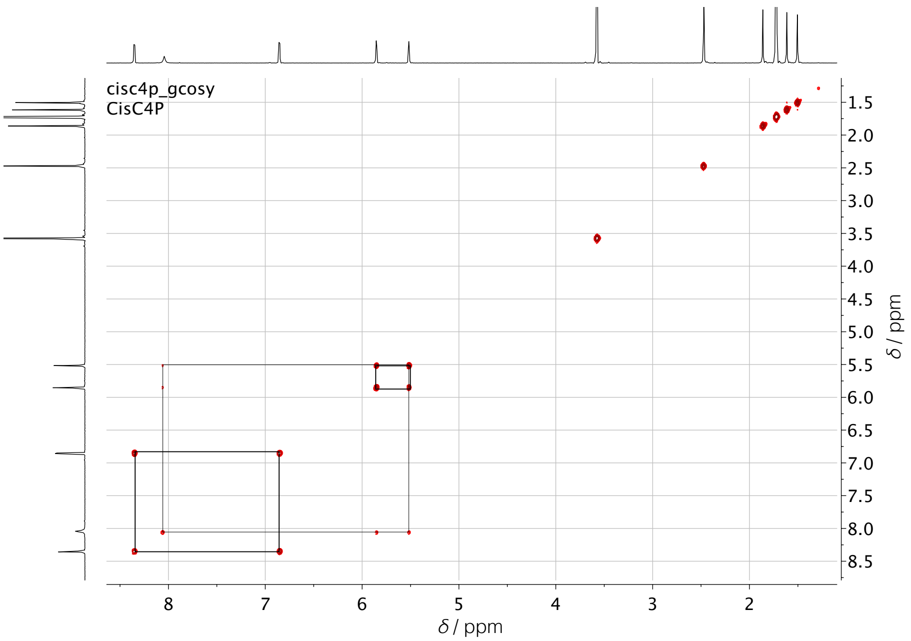

Supplementary Figure 4. COSY spectrum of **1** in THF-d_8.

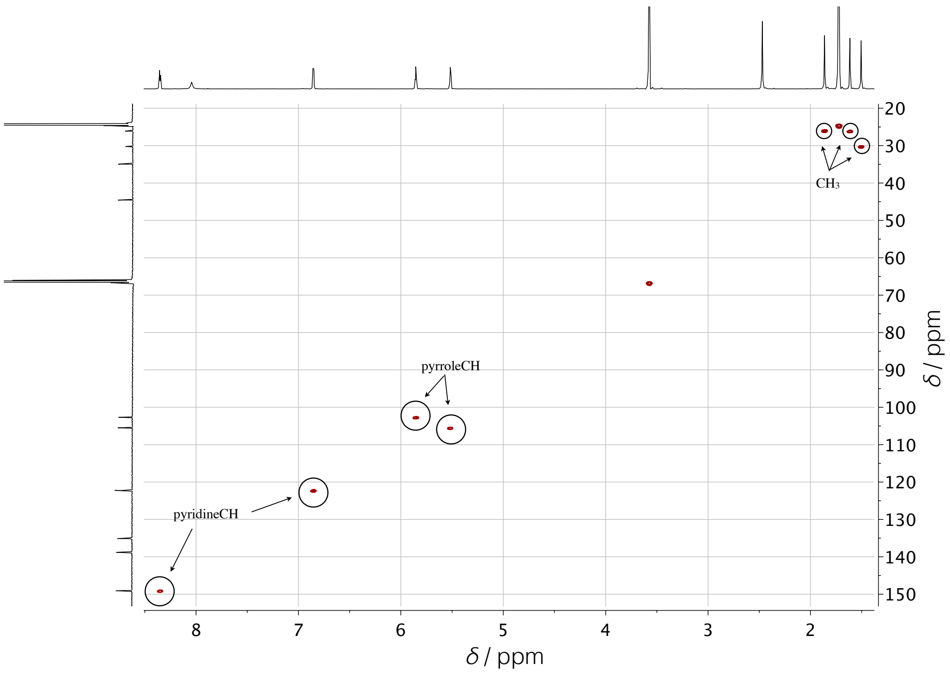

Supplementary Figure 5. HSQC spectrum of **1** in THF-d_8. CH_3 and CH carbons are phased up (red), and CH_2 carbons are phased down (blue).

Target Compound Screening Report

Results Acquired by The University of Texas at Austin Mass Spectrometry Facility

**Data File**	MSF18-0042(cisC4P)_hrESIpos1.d	**Sample Name**	0042(cisC4P)	**Comment**	0042(cisC4P)
**Position**	P1-F7	**Instrument Name**	Instrument 1	**User Name**	
**Acq Method**	pos.m	**Acquired Time**	1/17/2018 2:04:34 PM	**DA Method**	KS.m

MS Zoomed Spectrum

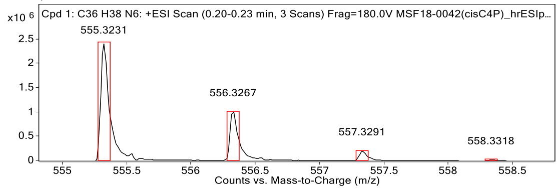

MS Spectrum Peak List

Obs. m/z	Calc. m/z	Charge	Abundance	Formula	Ion Species	Tgt Mass Error (ppm)
278.1646			2958315			
555.3231	555.3231	1	2418255	C36H38N6	(M+H)+	-0.03
556.3267	556.3261	1	1016417	C36H38N6	(M+H)+	-0.99
557.3291	557.3292	1	212113	C36H38N6	(M+H)+	0.05
558.3318	558.3322	1	26953	C36H38N6	(M+H)+	0.76
559.3325	559.3352	1	3944	C36H38N6	(M+H)+	4.92

--- End Of Report ---

Agilent Technologies Page 1 of 1 Printed at: 2:10 PM on:1/17/2018

Supplementary Figure 6. HRMS (ESI^+) spectrum of **1**.

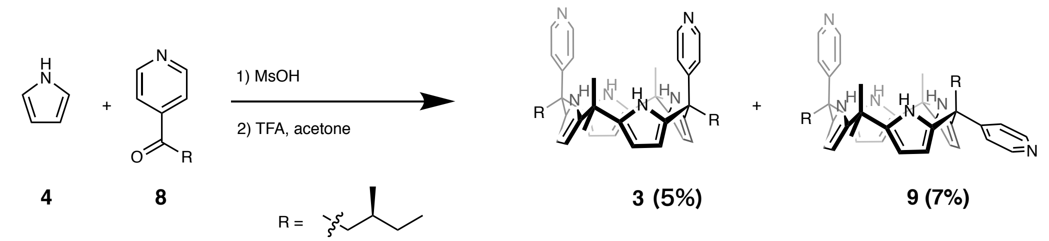

Supplementary Figure 7. Synthesis of **3**.

Supplementary Note 2. Synthetic procedure of **3** and **9**.

Synthesis of 5,10,15,20,22,24-Hexahydro-5,5,15,15-tetramethyl-10$^{\alpha}$,20$^{\alpha}$-di-(4-pyridinyl)-10$^{\beta}$,20$^{\beta}$-bis[{2-(*S*)-(–)-methyl}butyl]-21*H*,23*H*-porphine (**3**) and its configurational isomer, 5,10,15,20,22,24-hexahydro-5,5,15,15-tetramethyl-10$^{\alpha}$,20$^{\beta}$-di-(4-pyridinyl)-10$^{\beta}$,20$^{\alpha}$-bis[{2-(*S*)-(–)-methyl}butyl]-21*H*,23*H*-porphine (**9**): To a solution of **8** (3.88 g, 21.9 mmol) in 45 mL of pyrrole (the solvent) was added methanesulfonic acid (5.7 mL, 87 mmol) dropwise at 0°C. The mixture was allowed to warm to room temperature and then stirred for 8 h under a nitrogen atmosphere. Triethylamine (4.3 mL) was added to the reaction vessel and the resulting mixture was extracted with CH_2Cl_2. The organic layer was washed with saturated aqueous NaCl, dried over Na_2SO_4, and concentrated *in vacuo*. The resulting oil was dissolved in acetone (75 mL) and trifluoroacetic acid (1.6 mL, 21 mmol) was added at room temperature. The reaction mixture stirred for 8 hours and quenched with triethylamine (4.0 mL). The resulting mixture was extracted with CH_2Cl_2. The organic layer was washed with saturated aqueous NaCl, dried over Na_2SO_4, and concentrated *in vacuo*. Column chromatography on silica gel (0%–30% ethyl acetate in *n*-hexane, eluent) gave the desired product **3** (5%, for 2 steps) and its isomer **9** (7%, for 2 steps).

Supplementary Note 3. conpound data for **3** and **9**

Compound **3**: M.p. 200 °C (decomp.); $[\alpha]^{33}_{D}$ = –35.7 $cm^3g^{-1}dm^{-1}$; ^{1}H NMR (400 MHz, $CDCl_3$): δ 8.47 (dd, J_1 = 1.6 Hz, J_2 = 4.4 Hz, 4H), 7.21 (br, 2H), 7.16 (dd, J_1 = 1.6 Hz, J_2 = 4.4 Hz, 4H), 7.13 (br, 2H), 5.83–5.95 (m, 8H), 2.00–2.24 (m, 4H), 1.54 (s, 6H), 1.44 (s, 6H), 1.42 (m, overlapping, 2H), 0.93–1.13 (m, 4H), 0.73 (t, J = 7.4 Hz, 6H), 0.61 (d, J = 6.7 Hz, 6H) ppm; ^{13}C NMR (100 MHz, $CDCl_3$): δ 153.8, 149.3, 139.0, 138.8, 133.9, 133.6, 123.4, 107.1, 106.5, 103.2, 103.1, 48.9, 46.8, 35.1, 31.1, 30.8, 29.4, 28.7, 20.5, 11.3 ppm; FTIR (neat): ν 2959, 1719, 1596, 1458, 1415, 1378, 1362, 1206, 1073, 1044, 1003, 767, 709 cm^{-1}; HRMS (ESI$^+$) calcd. for $C_{44}H_{54}N_6Na$ *m/z* 689.4302 $[M+Na]^+$, found *m/z* 689.4305.

Compound **9**: M.p. 220 °C (decomp.); $[\alpha]^{33}_{D}$ = –12.0 $cm^3g^{-1}dm^{-1}$; ^{1}H NMR (400 MHz, $CDCl_3$): δ 8.47 (dd, J_1 = 1.5 Hz, J_2 = 4.7 Hz, 4H), 7.24 (dd, J_1 = 1.5 Hz, J_2 = 4.7 Hz, 4H), 7.17 (br, 2H), 7.14 (br, 2H), 5.90–5.95 (m, 8H), 2.05–2.26 (m, 4H), 1.46 (s, 6H), 1.44 (s, 6H), 1.42 (m, overlapping, 2H), 0.90–1.10 (m, 4H), 0.72 (t, J = 7.6 Hz, 6H), 0.61 (d, J = 6.6 Hz, 6H) ppm; ^{13}C NMR (100 MHz, $CDCl_3$): δ 153.8, 149.4, 139.0, 133.8, 133.6, 123.2, 106.6, 106.5, 103.2, 103.2, 48.8, 46.7, 35.2, 35.1, 31.0, 30.8, 29.3, 29.2, 20.4, 11.3 ppm; FTIR (neat): ν 2971, 1739, 1601, 1457, 1412, 1380, 1361, 1202, 1074, 1045, 992, 769, 712 cm^{-1}; HRMS (ESI^+) calcd. for $C_{44}H_{54}N_6Na$ *m/z* 689.4302 $[M+Na]^+$, found *m/z* 689.4312.

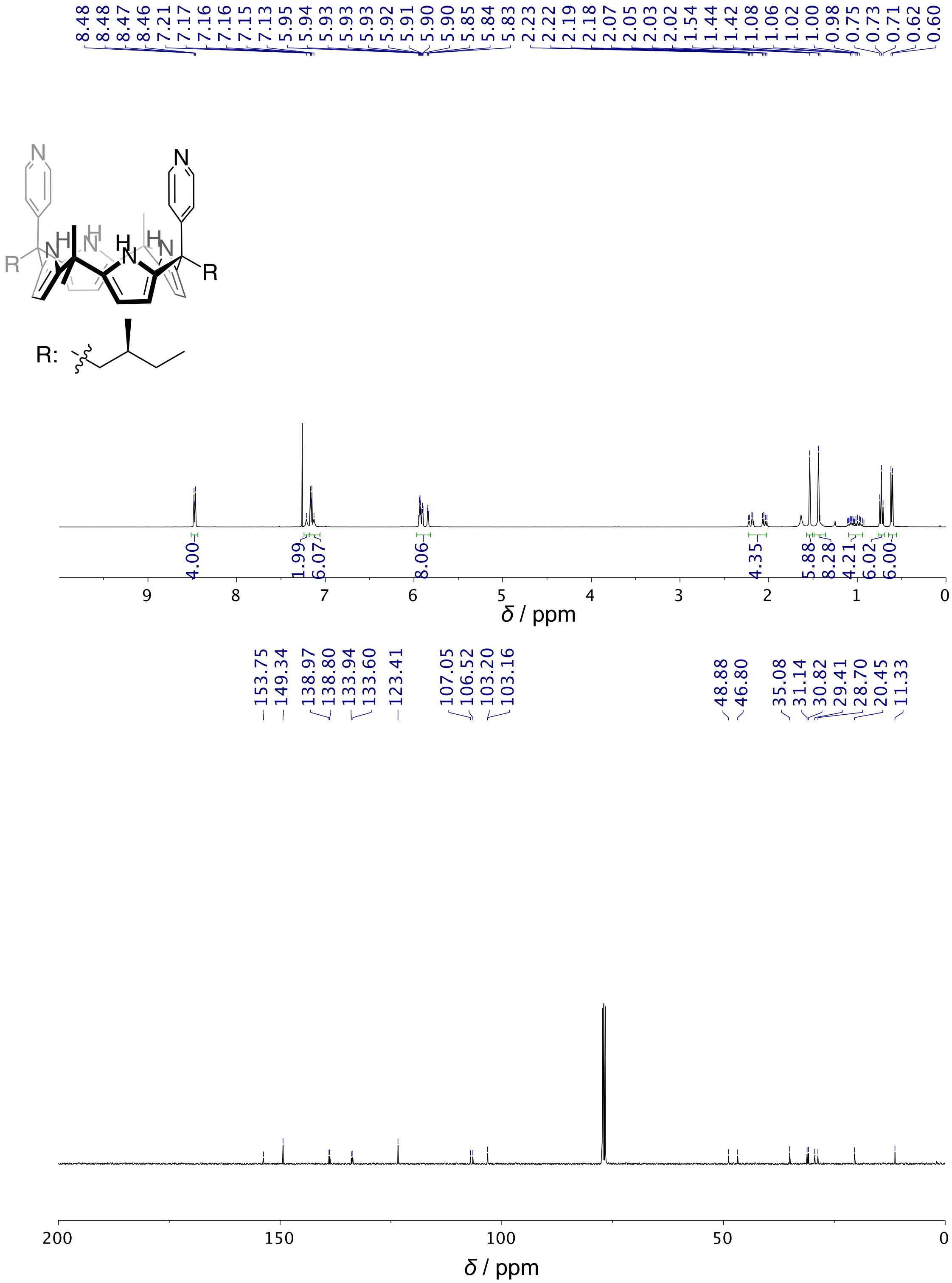

Supplementary Figure 8. ^{1}H and ^{13}C NMR spectra of **3** in chloroform-*d*.

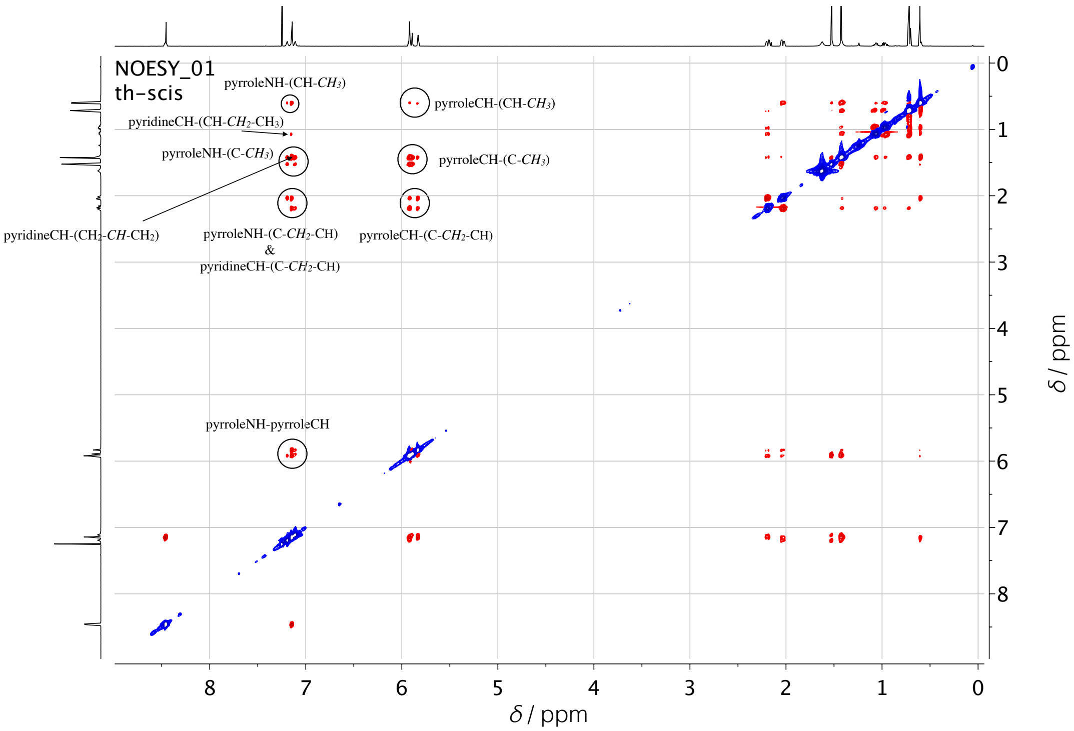

Supplementary Figure 9. NOESY spectrum of **3** in chloroform-*d*.

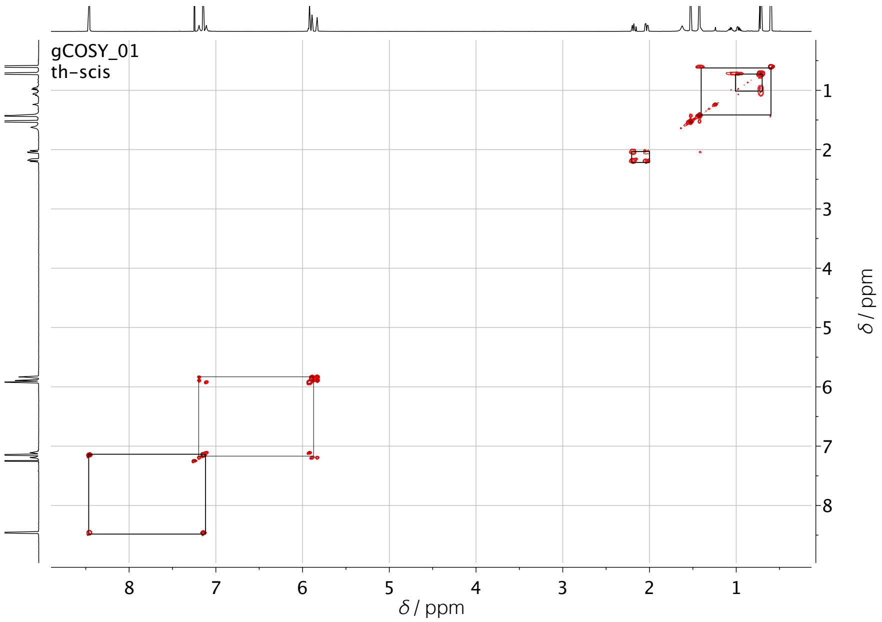

Supplementary Figure 10. COSY spectrum of **3** in chloroform-*d*.

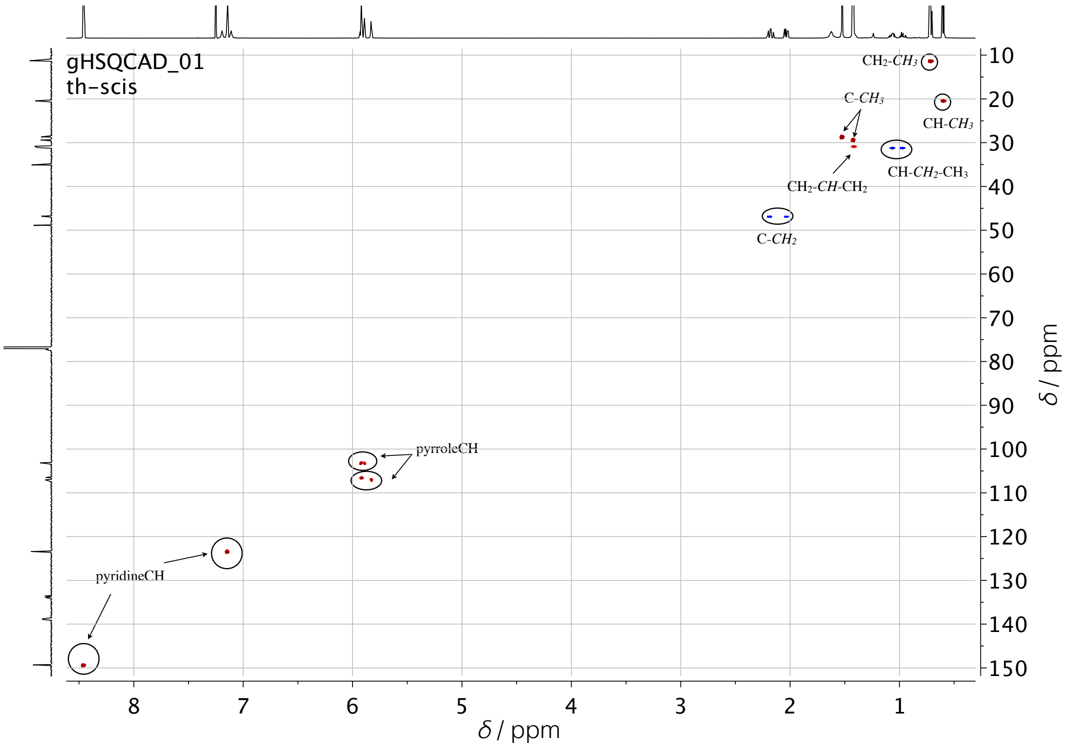

Supplementary Figure 11. HSQC spectrum of **3** in chloroform-*d*. CH_3 and CH carbons are phased up (red), and CH_2 carbons are phased down (blue).

Target Compound Screening Report

**Data File**	MSF16-3454(thscis)_hrESIpos1.d	**Sample Name**	3454(thscis)	**Comment**	3454(thscis)
**Position**	P1-A9	**Instrument Name**	Instrument 1	**User Name**	
**Acq Method**	pos.m	**Acquired Time**	9/29/2016 2:34:52 PM	**DA Method**	KS.m

MS Zoomed Spectrum

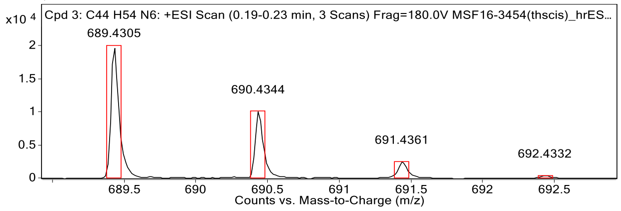

MS Spectrum Peak List

Obs. m/z	Calc. m/z	Charge	Abund	Formula	Ion/Isotope	Tgt Mass Error (ppm)
334.22930			1024778.29			
689.43050	689.43020	1	19778.29	C44H54N6	(M+Na)+	-0.39
690.43440	690.43330	1	10217.56	C44H54N6	(M+Na)+	-1.58
691.43610	691.43640	1	2596.54	C44H54N6	(M+Na)+	0.53
692.43320	692.43950	1	510.63	C44H54N6	(M+Na)+	9.23

--- End Of Report ---

Agilent Technologies Page 1 of 1 Printed at: 2:59 PM on:9/29/2016

Supplementary Figure 12. HRMS (ESI^+) spectrum of **3**.

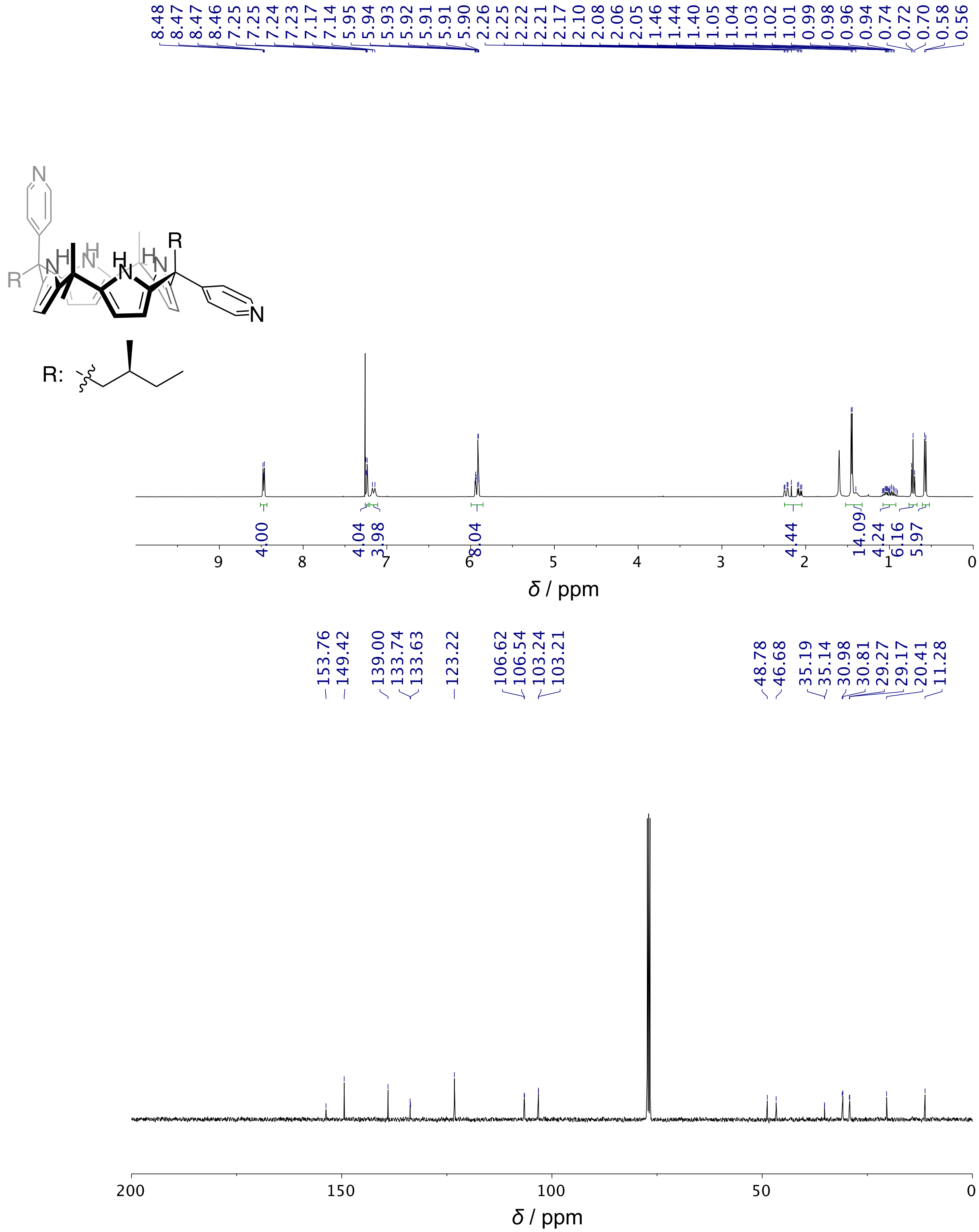

Supplementary Figure 13. ^{1}H and ^{13}C NMR spectra of **9** in chloroform-*d*.

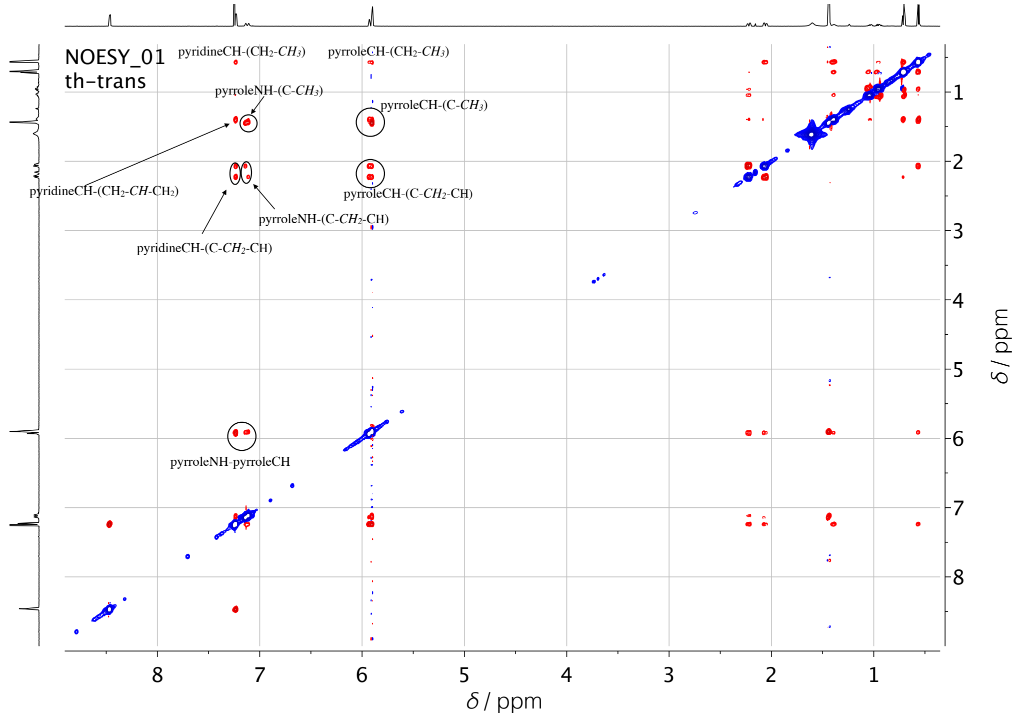

Supplementary Figure 14. NOESY spectrum of **9** in chloroform-*d*.

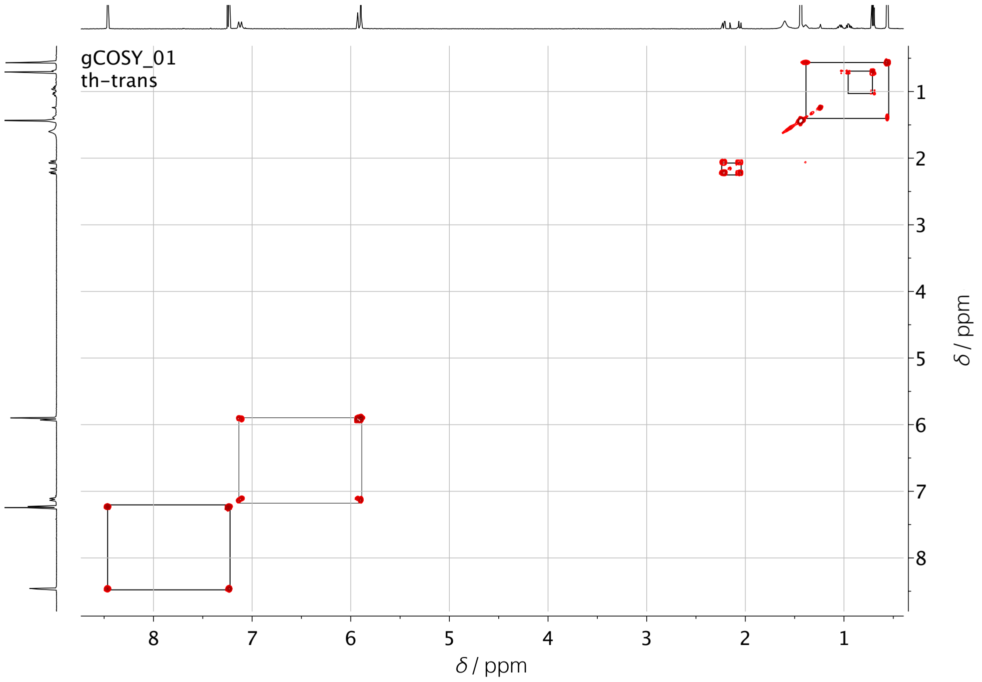

Supplementary Figure 15. COSY spectrum of **9** in chloroform-*d*.

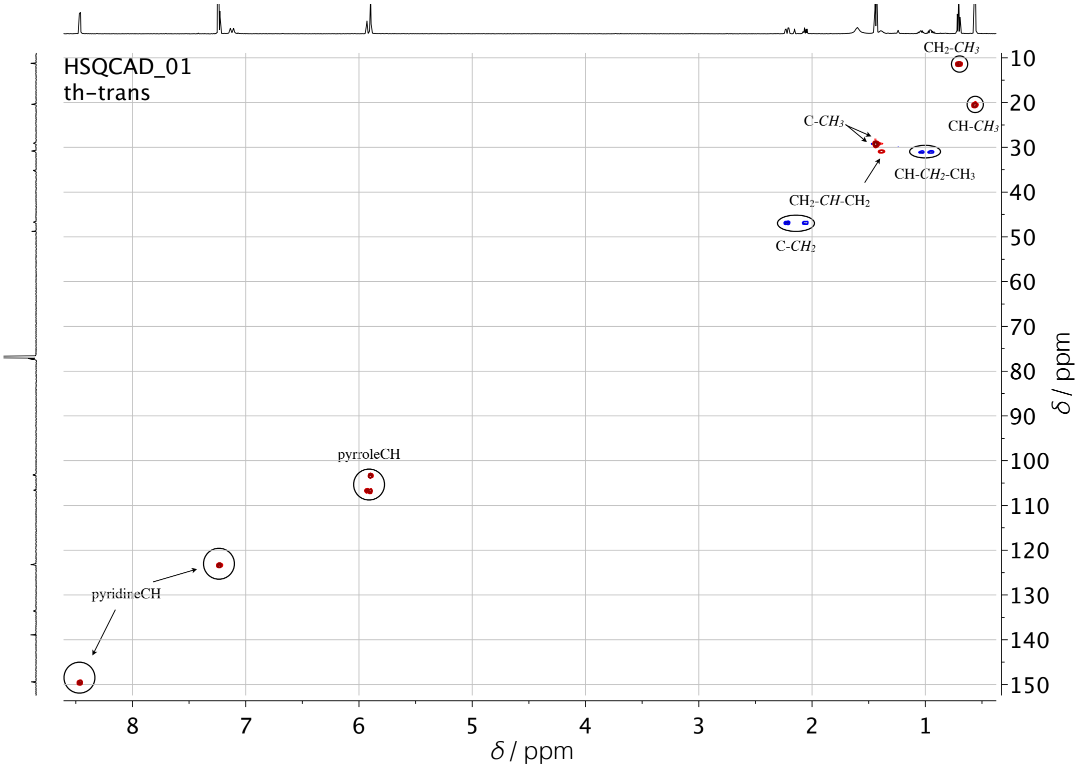

Supplementary Figure 16. HSQC spectrum of **9** in chloroform-*d*. CH_3 and CH carbons are phased up (red), and CH_2 carbons are phased down (blue).

Target Compound Screening Report

**Data File**	MSF16-3453(thstrans)_hrESIpos1.d	**Sample Name**	3453(thstrans)	**Comment**	3453(thstrans)
**Position**	P1-B2	**Instrument Name**	Instrument 1	**User Name**	
**Acq Method**	pos.m	**Acquired Time**	9/29/2016 2:37:42 PM	**DA Method**	KS.m

MS Zoomed Spectrum

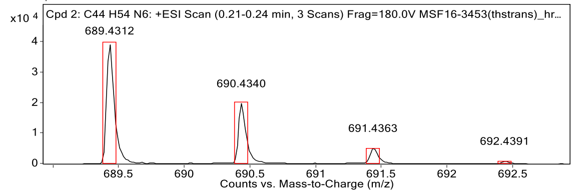

MS Spectrum Peak List

Obs. m/z	Calc. m/z	Charge	Abund	Formula	Ion/Isotope	Tgt Mass Error (ppm)
334.22920			1151939.02			
689.43120	689.43020	1	39172.95	C44H54N6	(M+Na)+	-1.49
690.43400	690.43330	1	19902.27	C44H54N6	(M+Na)+	-0.97
691.43630	691.43640	1	5233.54	C44H54N6	(M+Na)+	0.14
692.43910	692.43950	1	1001.93	C44H54N6	(M+Na)+	0.58
693.37950	693.44260	1	246.25	C44H54N6	(M+Na)+	91.1

--- End Of Report ---

Agilent Technologies Page 1 of 1 Printed at: 3:02 PM on:9/29/2016

Supplementary Figure 17. HRMS (ESI^+) spectrum of **9**.

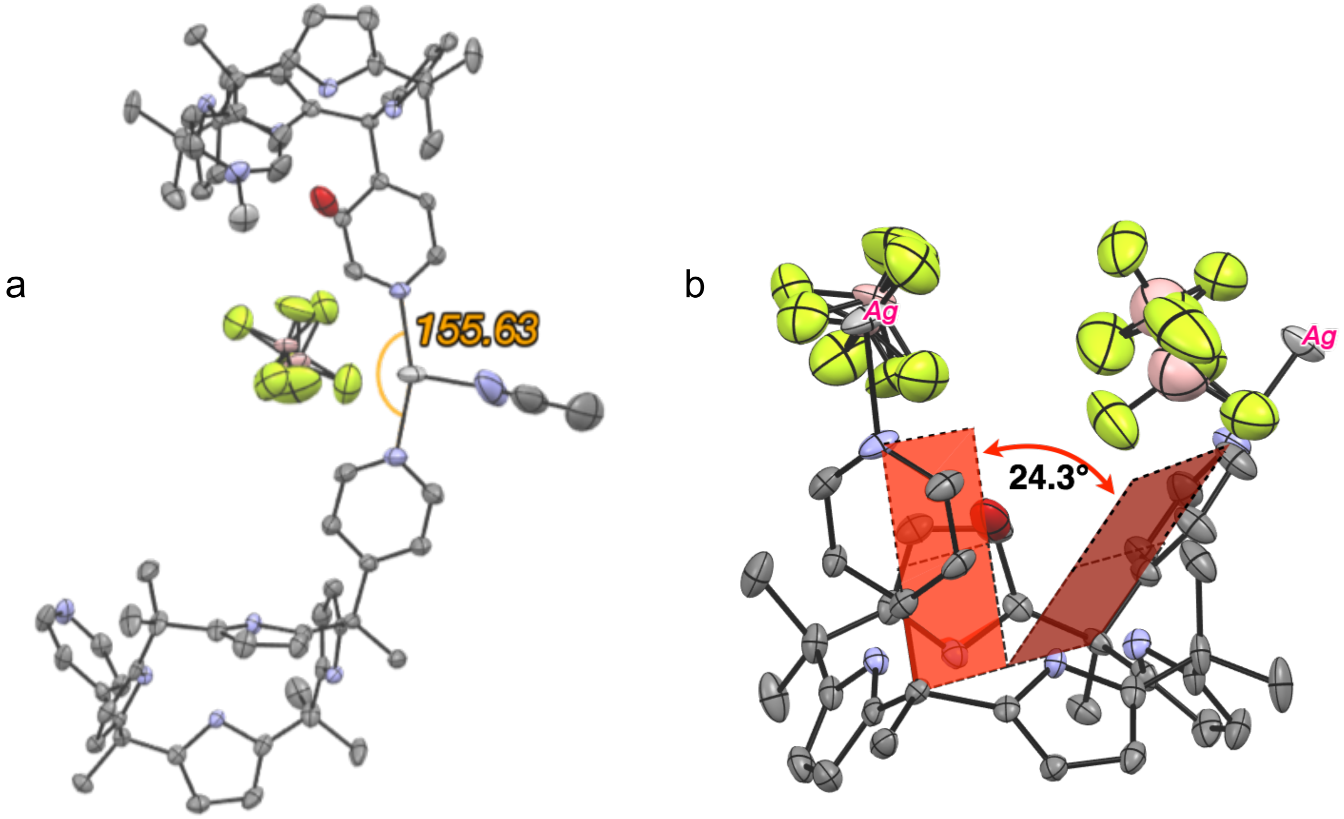

Supplementary Figure 18. Expanded single crystal structure of the helix formed from **1** and $AgBF_4$ highlighting (a) the angle of Pyr(N)-Ag-Pyr(N), the coordination geometry around the Ag(I) center, and (b) the torsion angle between two pyridine moieties.

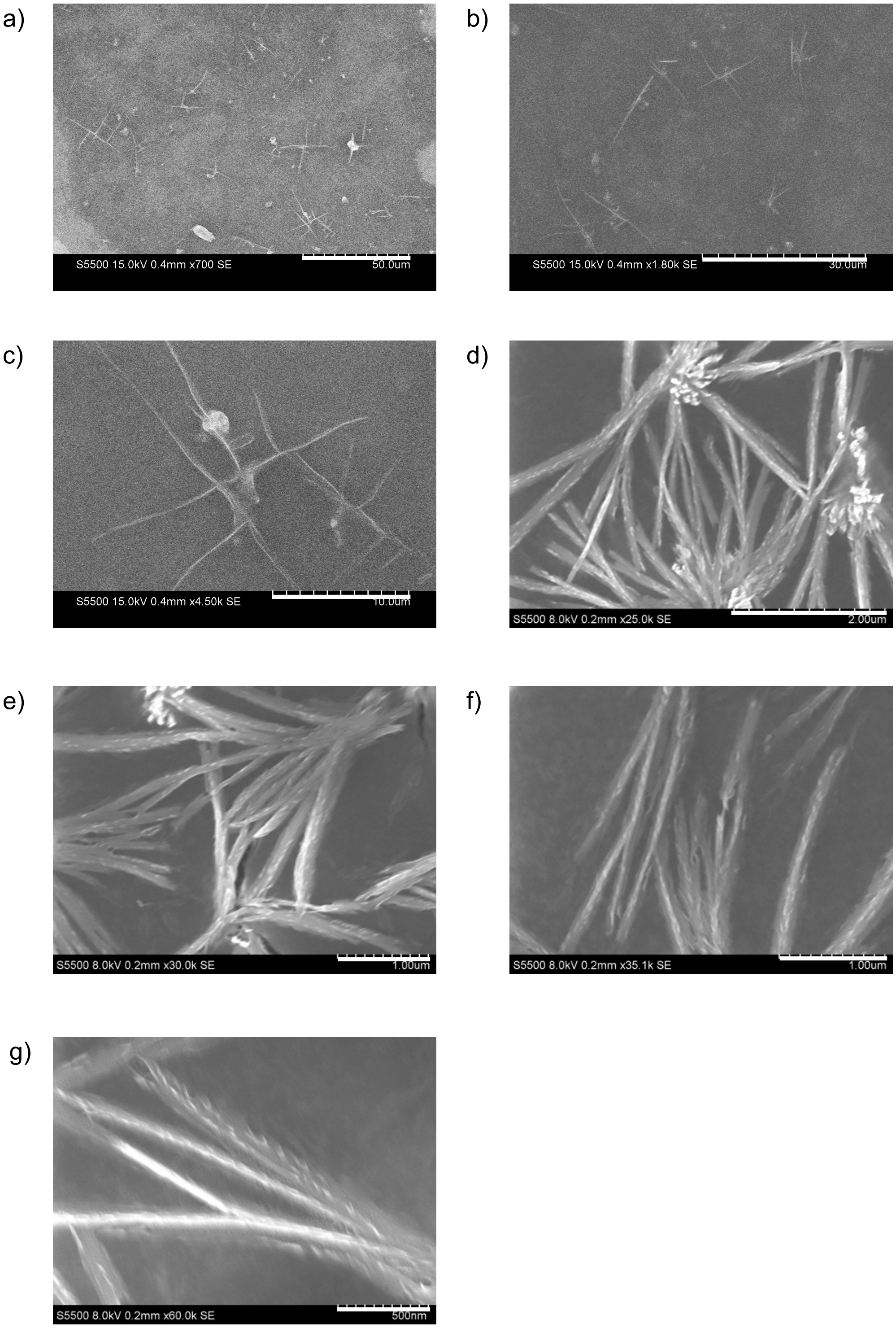

Supplementary Figure 19. SEM images of [**1**•Ag]$_\infty$. The scale bars denote 50 μm in (a), 30 μm in (b), 10 μm in (c), 2.0 μm in (d), 1.0 μm in (e), (f), and 0.50 μm in (g).

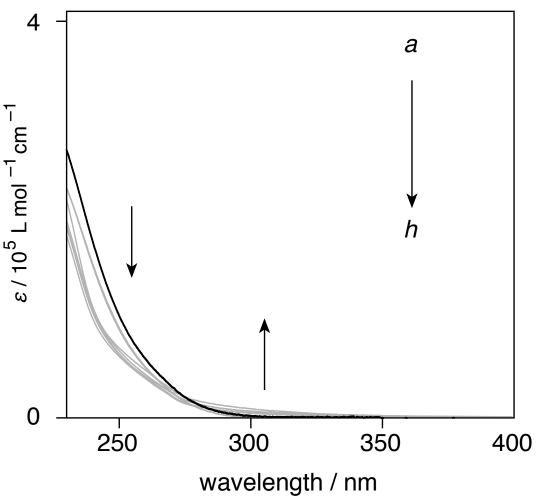

Supplementary Figure 20. UV-vis spectral absorption changes seen for a 1:1 mixture of **1** and $AgBF_4$ in THF. $AgBF_4$ Total concentrations are (a-h) 0.5, 0.8, 1.0, 2.0, 3.0, 5.0, 6.0, 7.0 x 10^{-5} mol L^{-1}.

m/*z* obs	*m*/*z* calc	Formula (charge state)	Error /ppm
661.2194	661.2203	Ag**1** (1+)	−1.5
1411.4397	1411.4458	$Ag_2\mathbf{1}_2BF_4$ (1+)	−4.3
1607.3489	1607.3521	$Ag_3\mathbf{1}_2(BF_4)_2$ (1+)	−2.0
2355.5820	2355.5805	$Ag_4\mathbf{1}_3(BF_4)_3$ (1+)	0.6

Supplementary Table 1. Cold spray ionization mass spectrometry (CSI-MS) data obtained from a mixture of **1** and $AgBF_4$ in chloroform/acetonitrile (1:1, v/v).

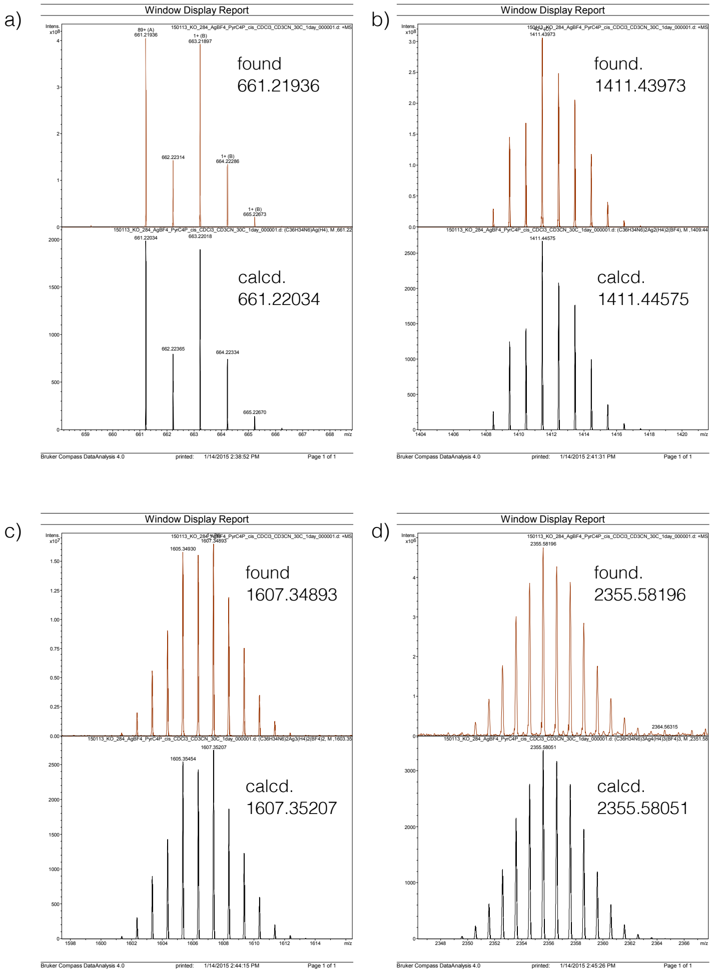

Supplementary Figure 21. CSI-MS spectra of **1** and $AgBF_4$ complexes: Observed elemental compositions are (a) $[\mathbf{1}\bullet Ag]^+$, (B) $[\mathbf{1}_2\bullet(AgBF_4)\bullet Ag]$+, (c) $[\mathbf{1}_2\bullet(AgBF_4)_2\bullet Ag]^+$and (d) $[\mathbf{1}_3\bullet(AgBF_4)_3\bullet Ag]^+$.

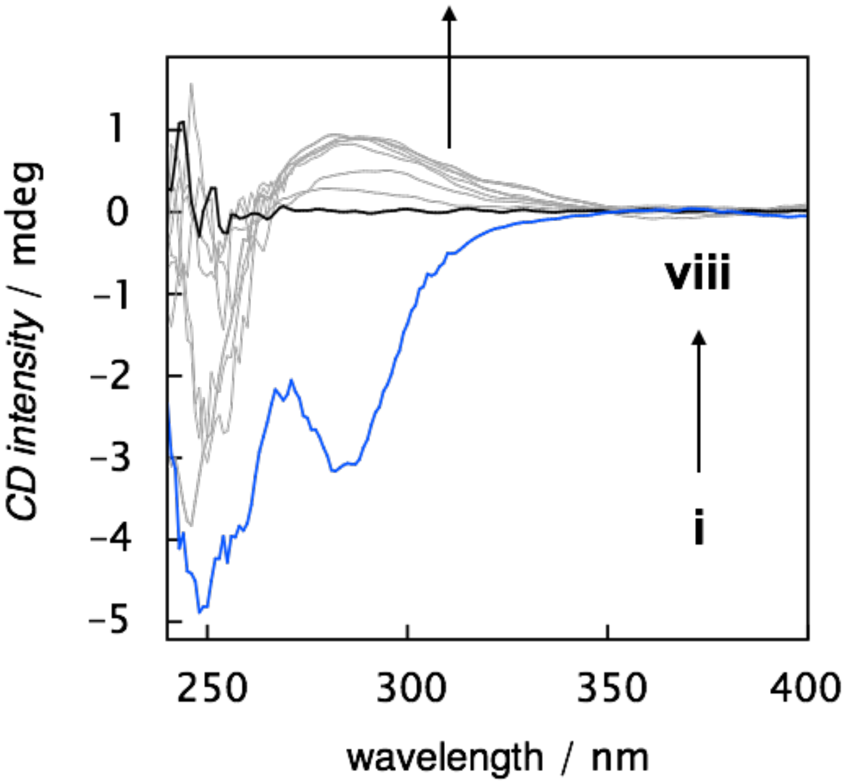

Supplementary Figure 22. CD spectral changes seen for mixtures of **1**, **3**, and $AgBF_4$ in THF at 25 °C. The ratios corresponding to the individual spectra are ([**1**] : [**3**] : [$AgBF_4$]) for traces i-viii are, respectively: 10:0:10, 9:1:10, 7:3:10, 4:6:10, 3:7:10, 2:8:10, 1:9:10, 0:10:10. The total concentrations of calix[4]pyrroles ([**1**] + [**3**]) and $AgBF_4$ are 1.3 x 10^{-4} mol L^{-1}. The blue line indicates the CD spectrum of **3** in the absence of $AgBF_4$ recorded at a concentration of 1.3 x 10^{-4} mol L^{-1}.

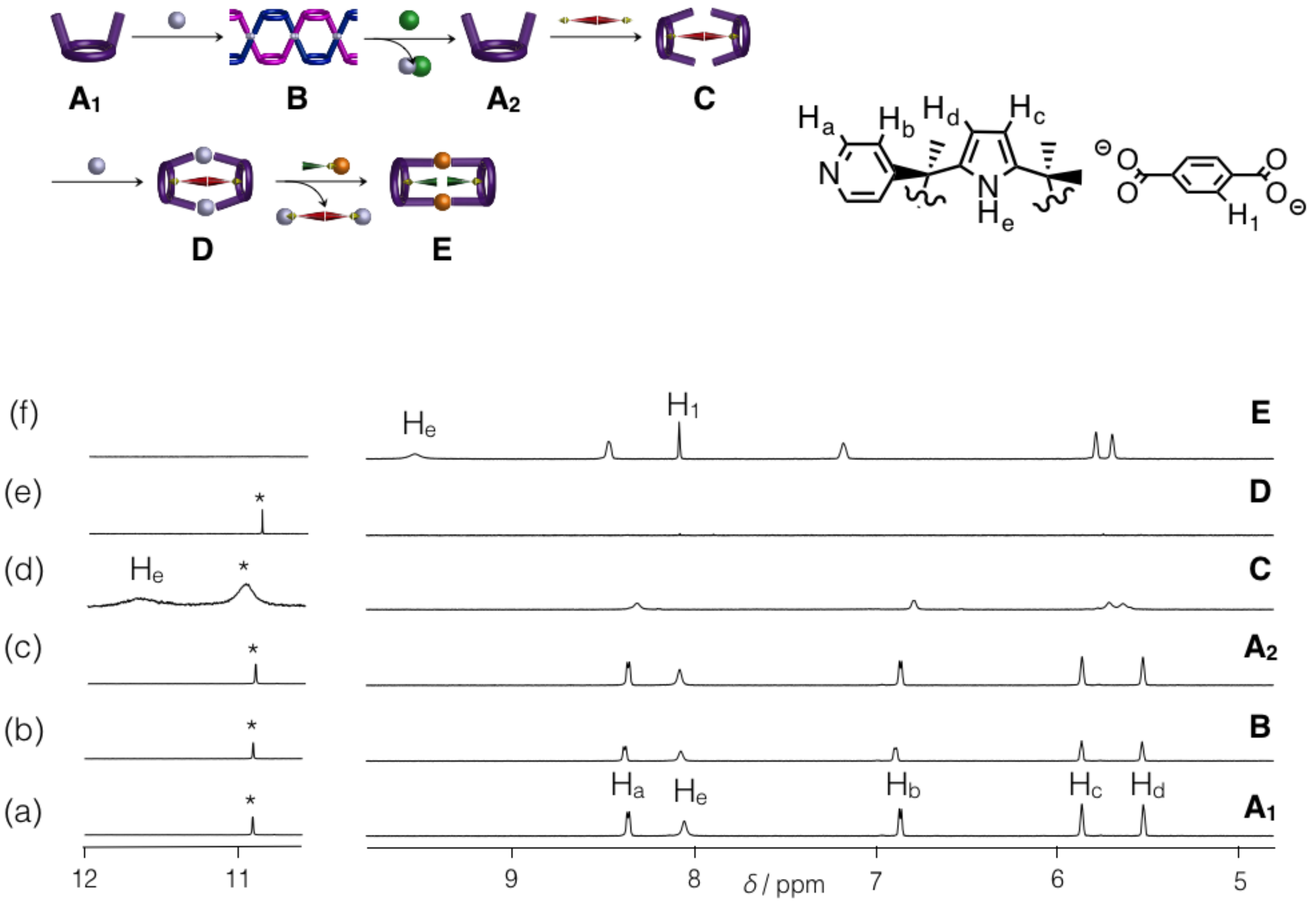

Supplementary Figure 23. Changes in the ^{1}H NMR spectrum of (a) **1** (8.0×10^{-4} mol L^{-1}) seen upon the sequential addition of (b) $AgBF_4$ (1.0 equiv.), (c) tetrabutylammonium chloride (TBACl) (1.0 equiv.), (d) the bisTBA salt of **2** (0.5 equiv.), (e) $AgBF_4$ (1.0 equiv.), and (f) TFA (1.0 equiv.). The spectra were recorded in THF-d_8. *Indicates solvent impurity.

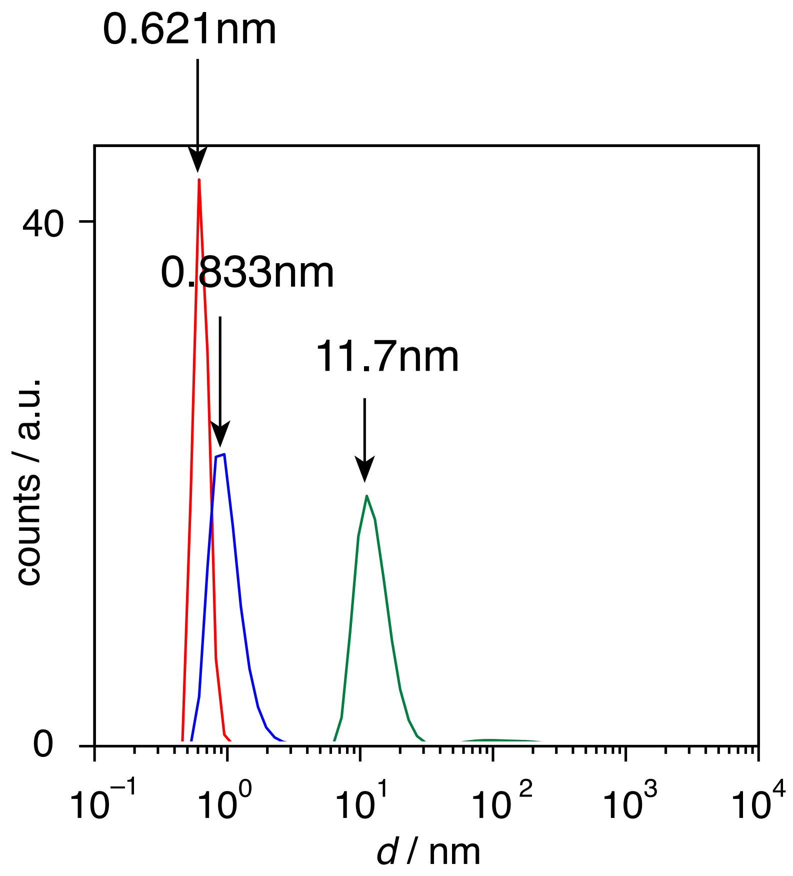

Supplementary Figure 24. DLS profiles of **1** and its complexes: Changes in the DLS profile seen upon the sequential addition of $AgBF_4$ and TBACl to the THF solution of **1** at 25°C, highlighting the reversible nature of transformations between monomeric **1** and the helical structure [**1**•Ag]$_\infty$. The spectra are: (red) **1** (1.0 x 10^{-5} $molL^{-1}$) only; (green) **1** (1.0 x 10^{-5} $molL^{-1}$) + $AgBF_4$ (1.0 x 10^{-5} $molL^{-1}$); (blue) **1** (1.0 x 10^{-5} $molL^{-1}$) + $AgBF_4$ (1.0 x 10^{-5} $molL^{-1}$) + TBACl (1.0 x 10^{-5} $molL^{-1}$).

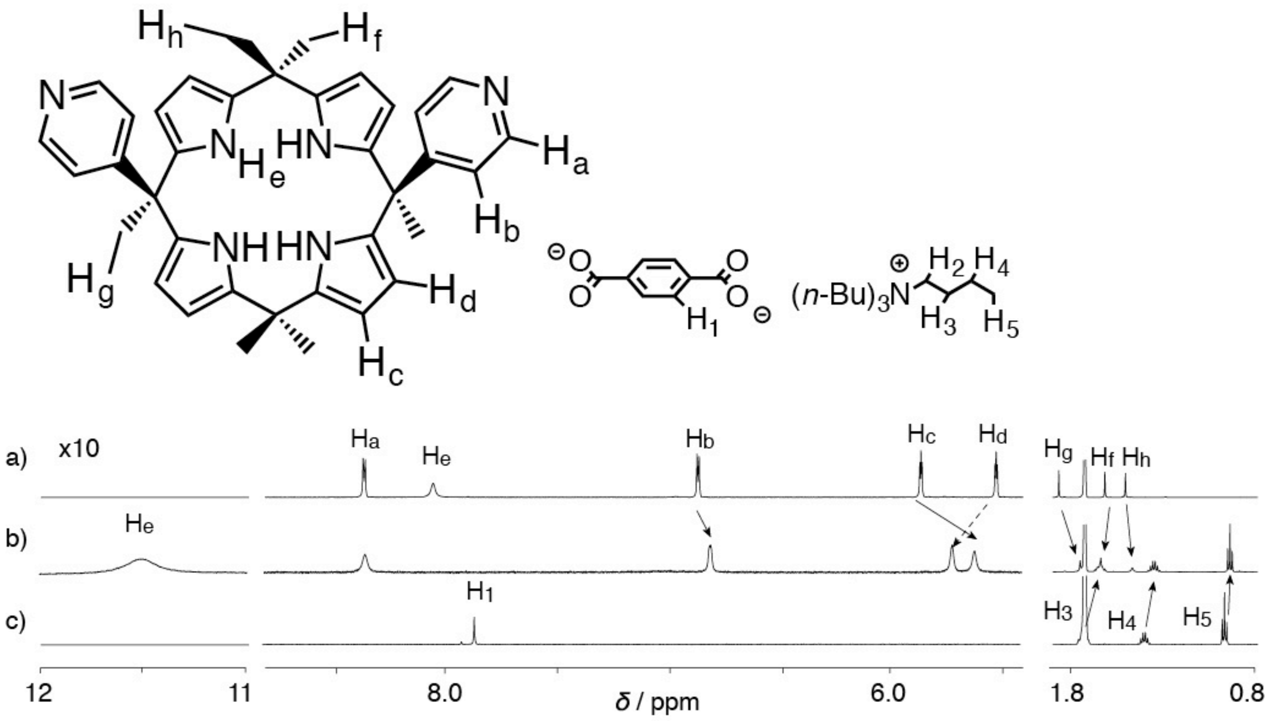

Supplementary Figure 25. ^{1}H NMR spectrum of (a) **1** (5.0 x 10^{-3} mol L^{-1}), (b) a 2:1 mixture of **1** (5.0 x 10^{-3} mol L^{-1}) and the bisTBA salt of **2** (2.5 x 10^{-3} mol L^{-1}), and (c) the bisTBA salt of pure **2** (2.5 x 10^{-3} mol L^{-1}) as recorded in THF-d_8.

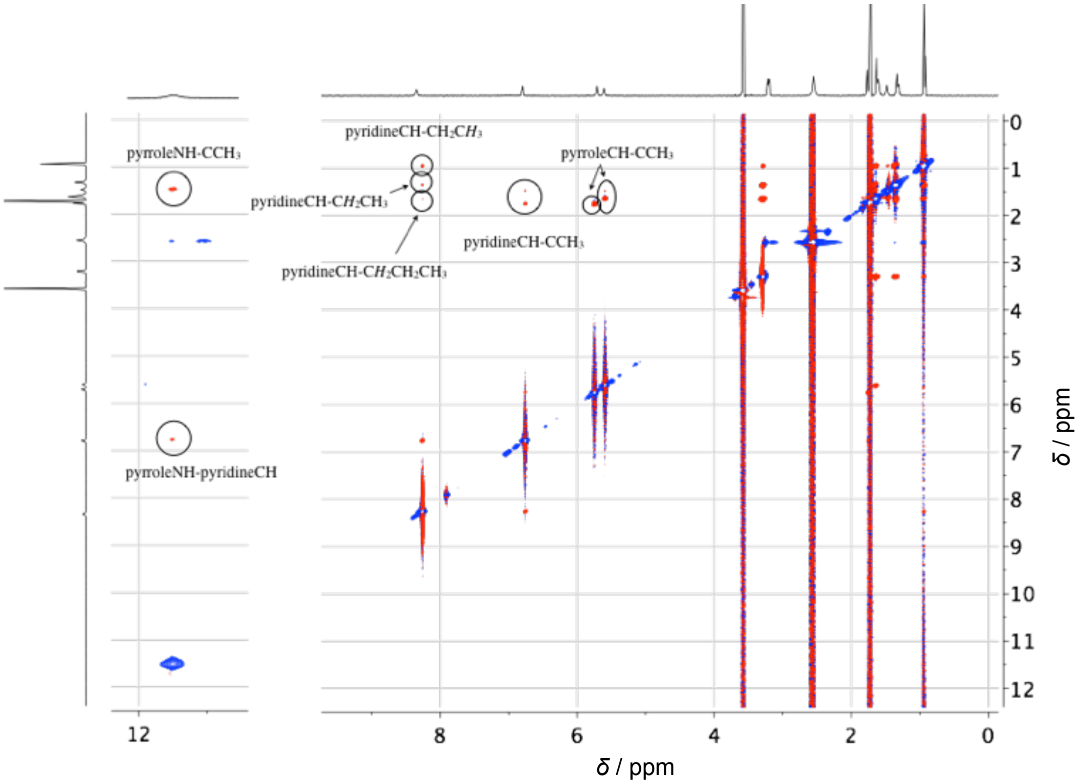

Supplementary Figure 26. NOESY spectrum of a 2:1 mixture of **1** and the bisTBA salt of **2** in THF-d_8.

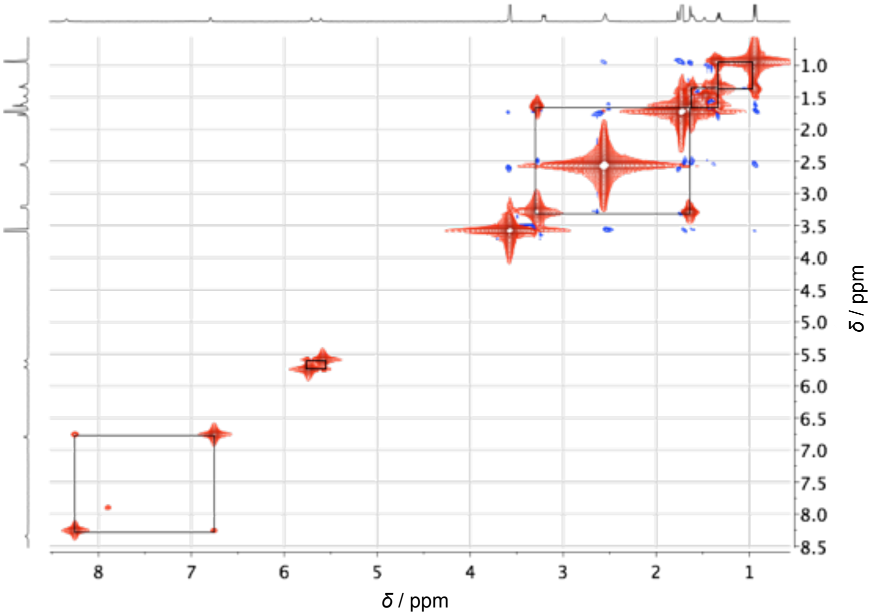

Supplementary Figure 27. COSY spectrum of a 2:1 mixture of **1** and the bisTBA salt of **2** recorded in THF-d_8.

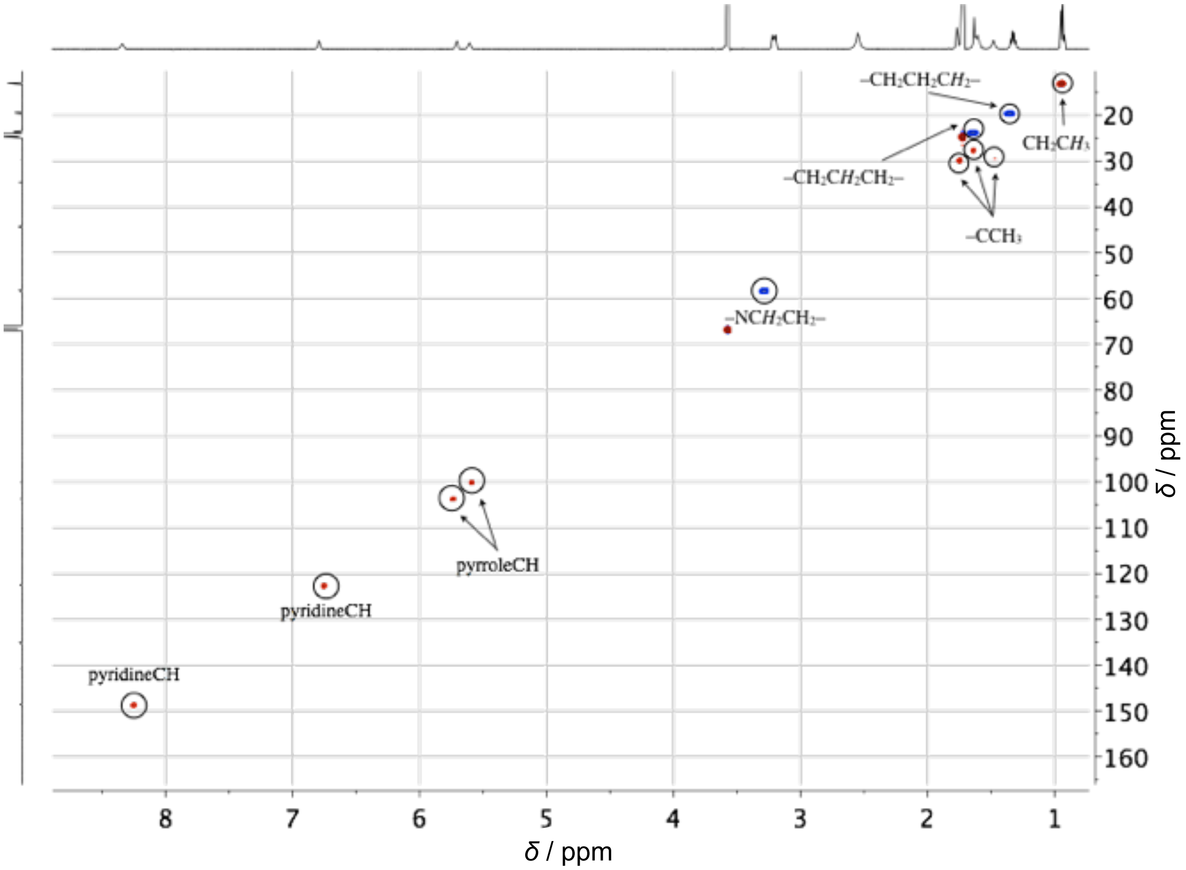

Supplementary Figure 28. HSQC spectrum of a 2:1 mixture of **1** and the bisTBA salt of **2** in THF-d_8. CH_3 and CH carbons are phased up (red), and CH_2 carbons are phased down (blue).

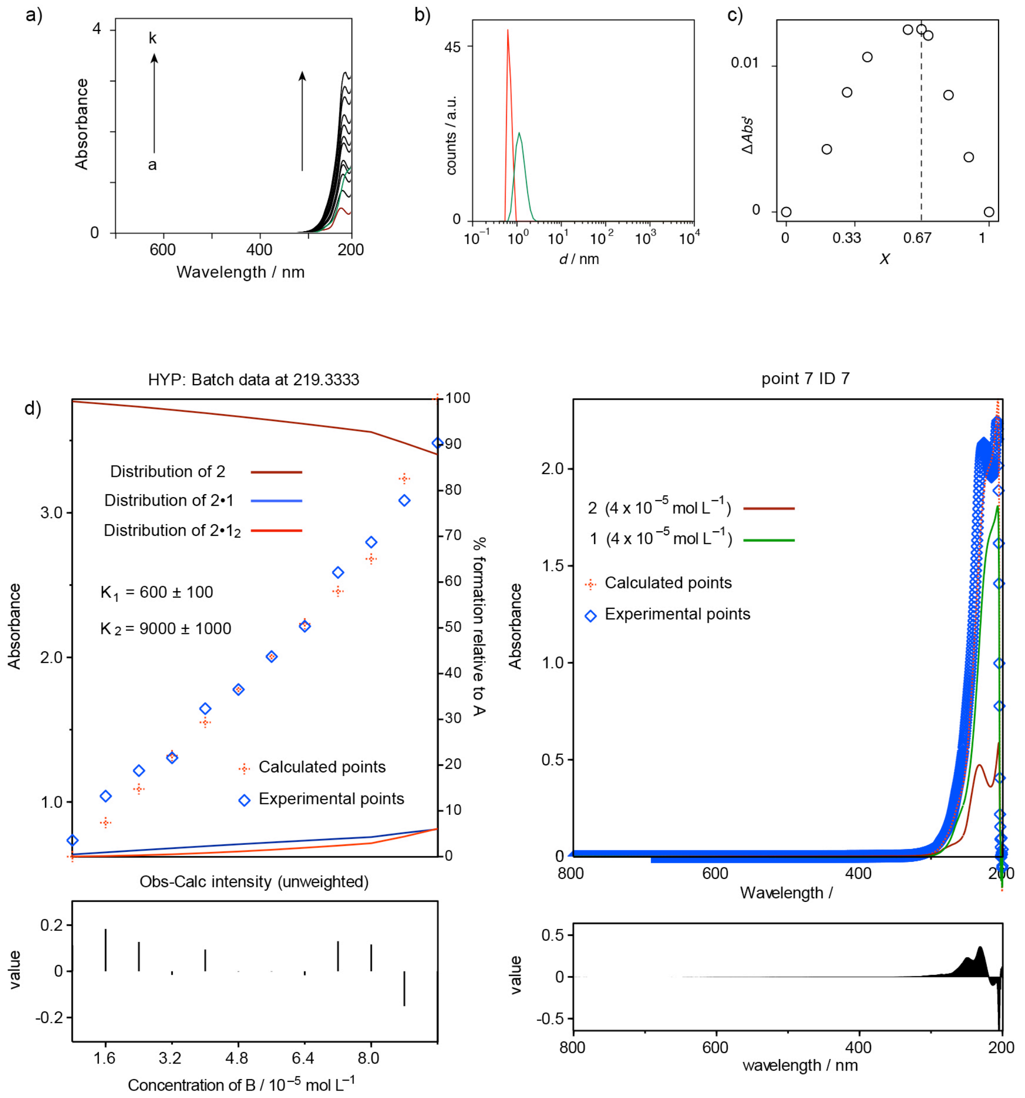

Supplementary Figure 29. (a) Changes in the UV-vis absorption spectrum of the bisTBA salt of **2** (4.0 x 10^{-5} mol L^{-1}) seen upon the addition of **1**. Concentrations of **1** are (a-k): 0.8, 1.6, 2.4, 3.2, 4.0, 4.8, 5.6, 6.4, 7.2, 8.0, 12 x 10^{-5} mol L^{-1}. The brown and green lines shown in (a) correspond to the UV-vis absorption spectra of the bisTBA salt of **2** (4.0 x 10^{-5} mol L^{-1}) and **1** (4.0 x 10^{-5} mol L^{-1}), respectively. The spectra were recorded in THF. (b) DLS profiles of (red) **1** (1.0 x 10^{-5} mol L^{-1}) and (green) a 2:1 mixture of **1** (1.0 x 10^{-5} mol L^{-1}) and the bisTBA salt of **2** (0.50 x 10^{-5} mol L^{-1} in THF at 25 °C. (c) Job plot for a mixture of **1** and the bisTBA salt of **2** ([**1**] + [**2**] = 1.0 x 10^{-4} mol L^{-1}) in THF. X indicates the mole fraction of **1**. ΔAbs' indicates $|Abs - Abs_1 X - Abs_2(1-X)|$. (d) Images exported from HypSpec Graphs[4] showing the fitting over the whole spectrum.

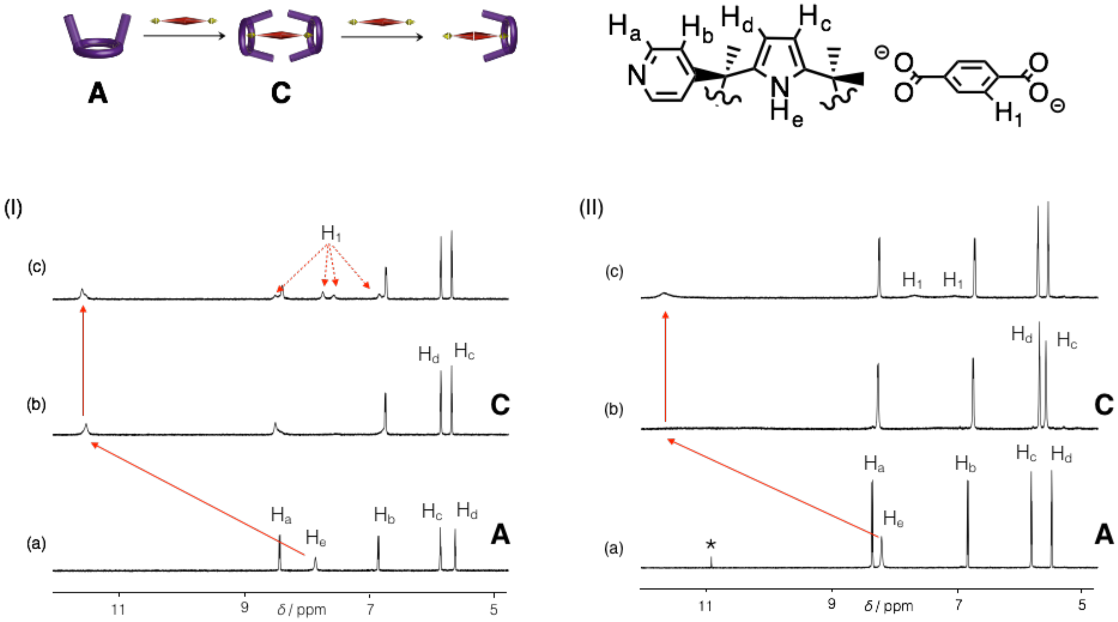

Supplementary Figure 30. (I) Changes in the ^{1}H NMR spectra of (a) **1** (2.0 x 10^{-3} molL^{-1}) seen upon the sequential addition of (b) the bisTBA salt of **2** (0.5 equiv.) and (c) additional 0.5 equiv. of **2** in MeCN-d_3. (II) Changes seen from the same experiments in THF-d_8.

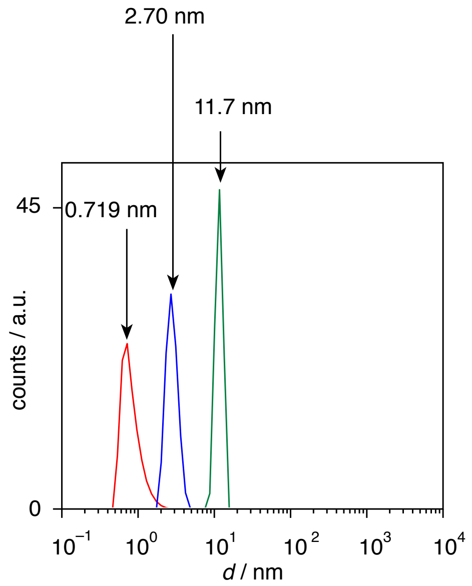

Supplementary Figure 31. Changes in the DLS profile seen upon the sequential addition of $AgBF_4$ and the bisTBA salt of **2** to the THF solution of **1** at 25°C. The spectra are: (red) **1** (1.0 x 10^{-5} mol L^{-1}) only; (green) **1** (1.0 x 10^{-5} mol L^{-1}) + $AgBF_4$ (1.0 x 10^{-5} mol L^{-1}); (blue) **1** (1.0 x 10^{-5} mol L^{-1}) + $AgBF_4$ (1.0 x 10^{-5} mol L^{-1}) + **2** (0.5 x 10^{-5} mol L^{-1}).

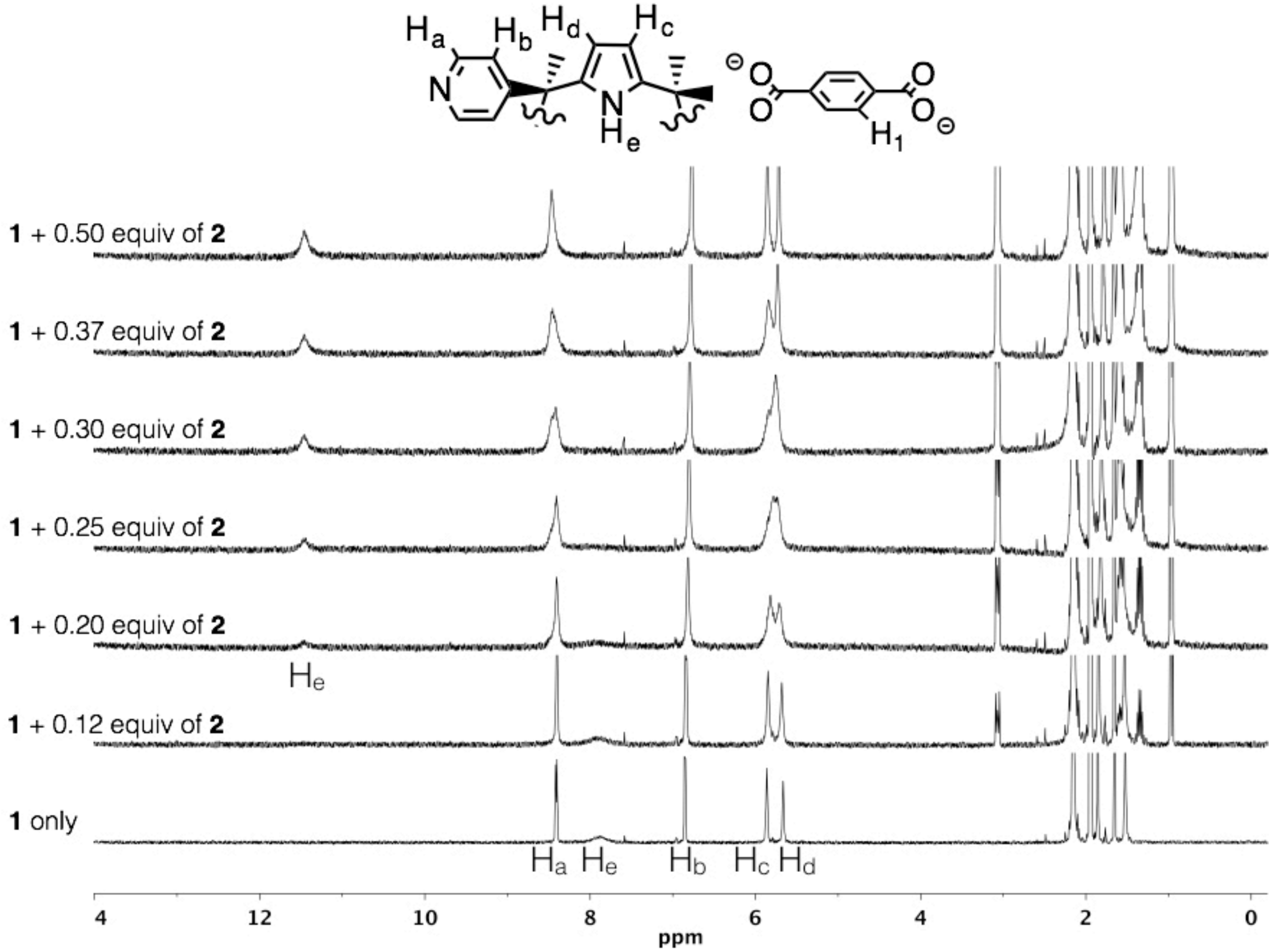

Supplementary Figure 32. Changes in the ^{1}H NMR spectrum of **1** (2.0×10^{-3} mol L^{-1}) seen upon the sequential addition of the bisTBA salt of **2**. The spectra were recorded in MeCN-d_3.

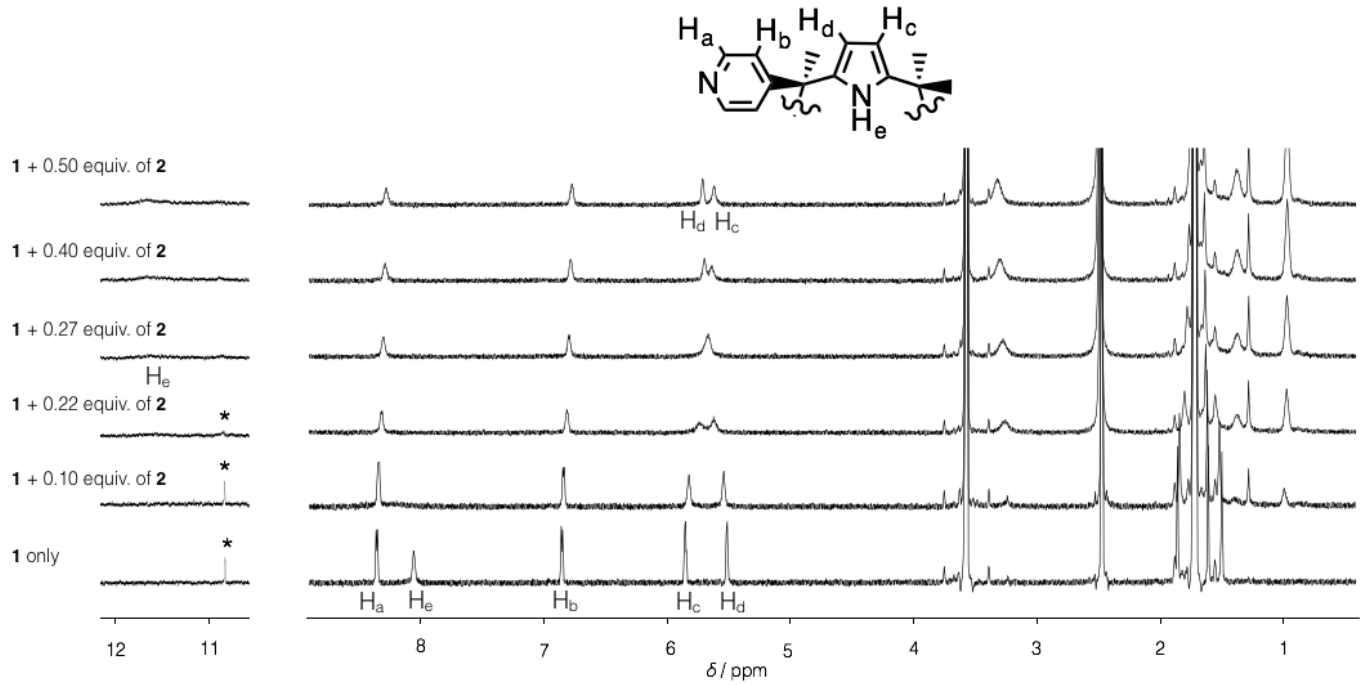

Supplementary Figure 33. Changes in the ^{1}H NMR spectrum of **1** (1.0×10^{-3} mol L^{-1}) seen upon the sequential addition of the bisTBA salt of **2**. The spectra were recorded in THF-d_8. *Indicates solvent impurity.

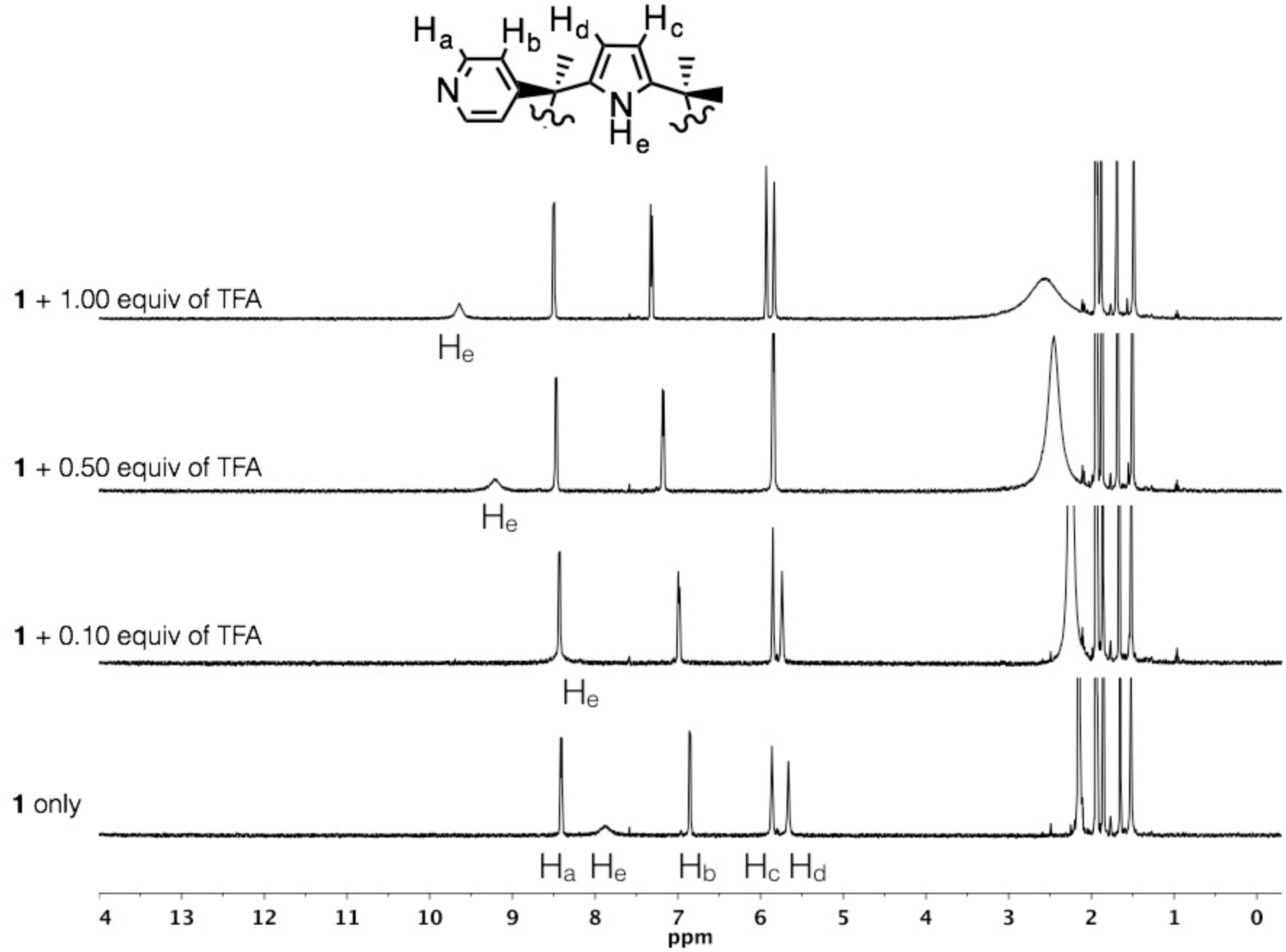

Supplementary Figure 34. Changes in the ^{1}H NMR spectrum of **1** (2.0×10^{-3} mol L^{-1}) seen upon the sequential addition of TFA. The spectra were recorded in MeCN-d_3.

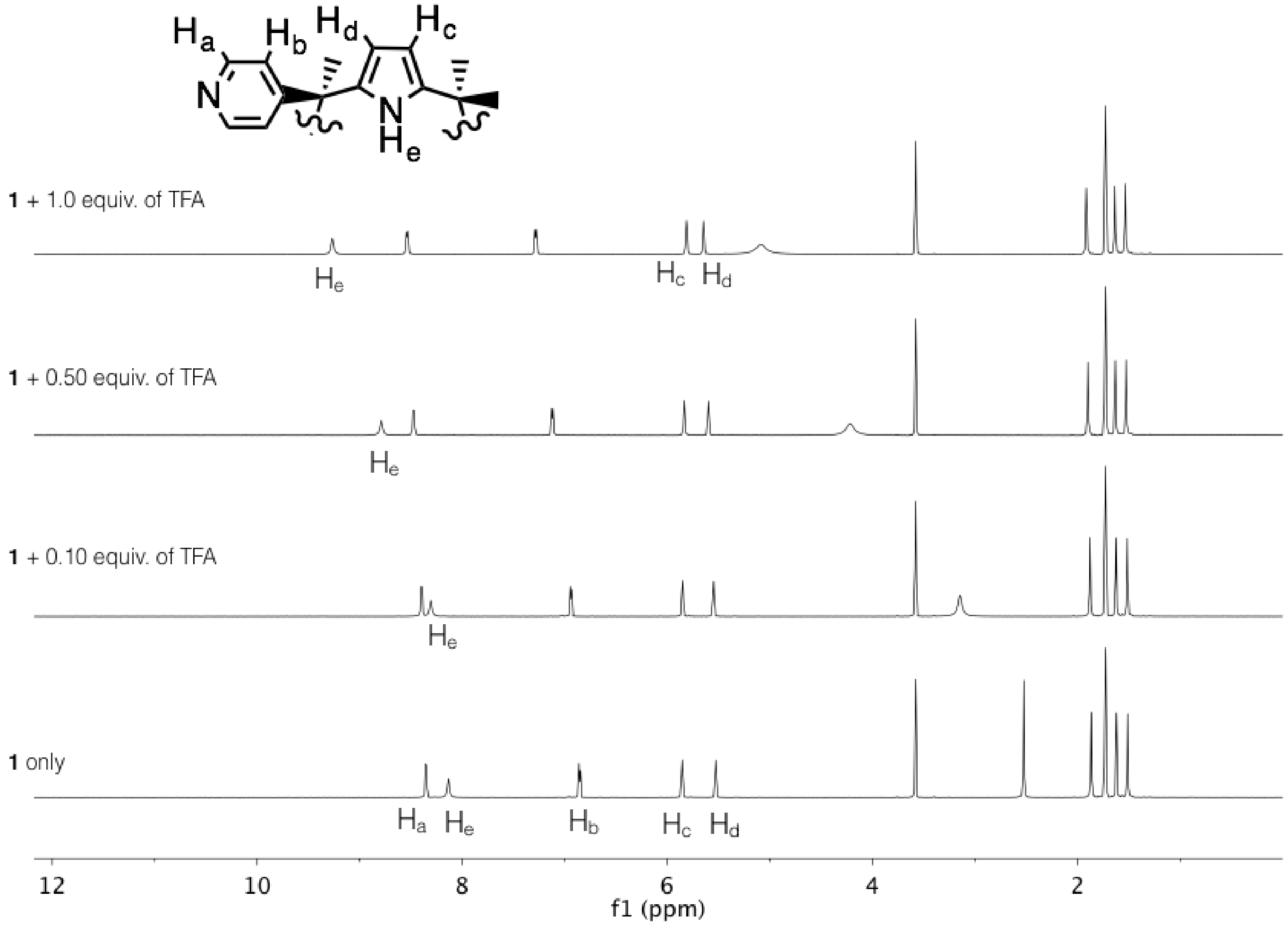

Supplementary Figure 35. Changes in the ^{1}H NMR spectrum of **1** (10 x 10^{-3} mol L^{-1}) seen upon the sequential addition of TFA. The spectra were recorded in THF-d_8.

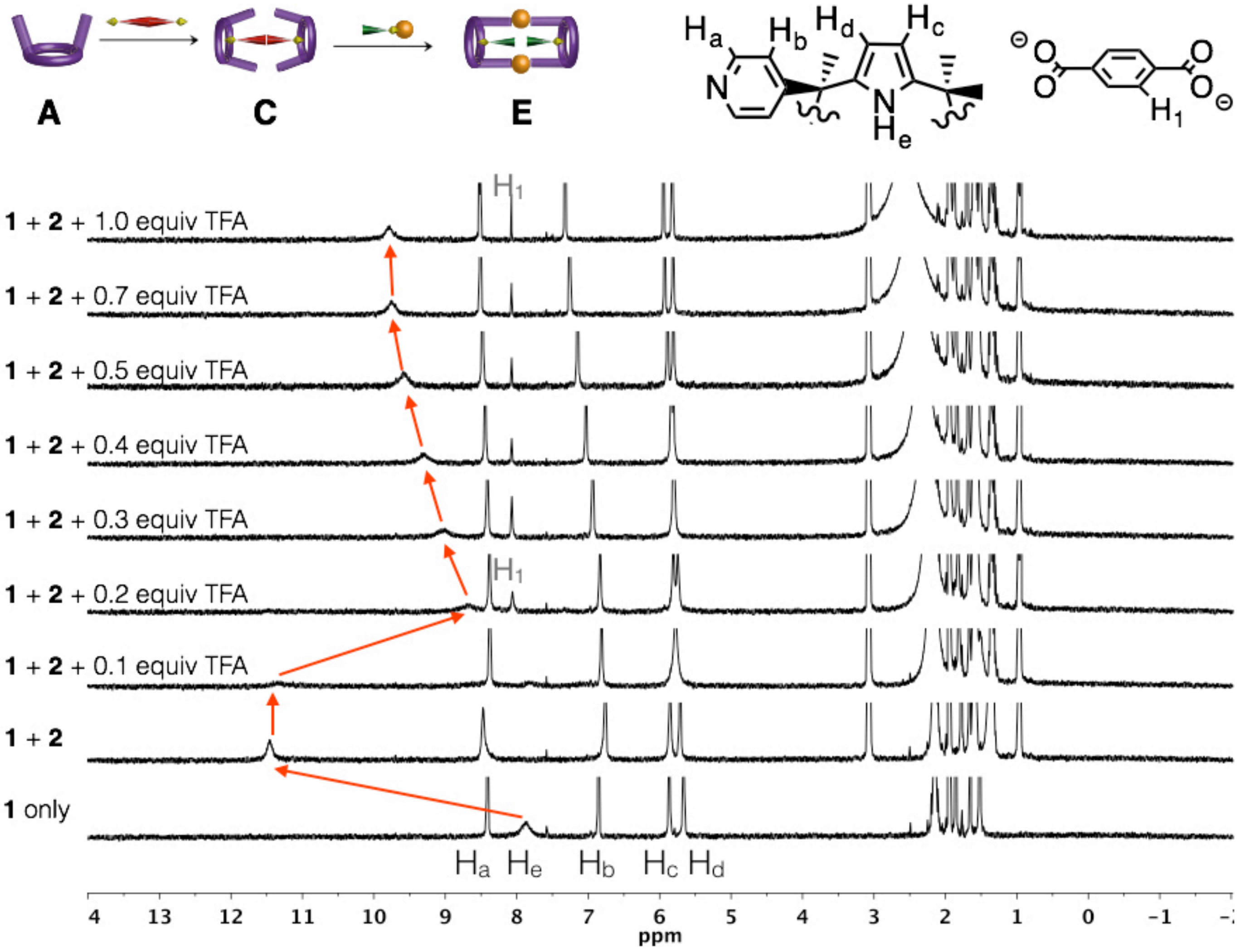

Supplementary Figure 36. Changes in the ^{1}H NMR spectrum of a 2:1 mixture of **1** (2.0 x 10^{-3} mol L^{-1}) and the bisTBA salt of **2** (1.0 x 10^{-3} mol L^{-1}) seen upon the sequential addition of TFA. The spectra were recorded in MeCN-d_3.

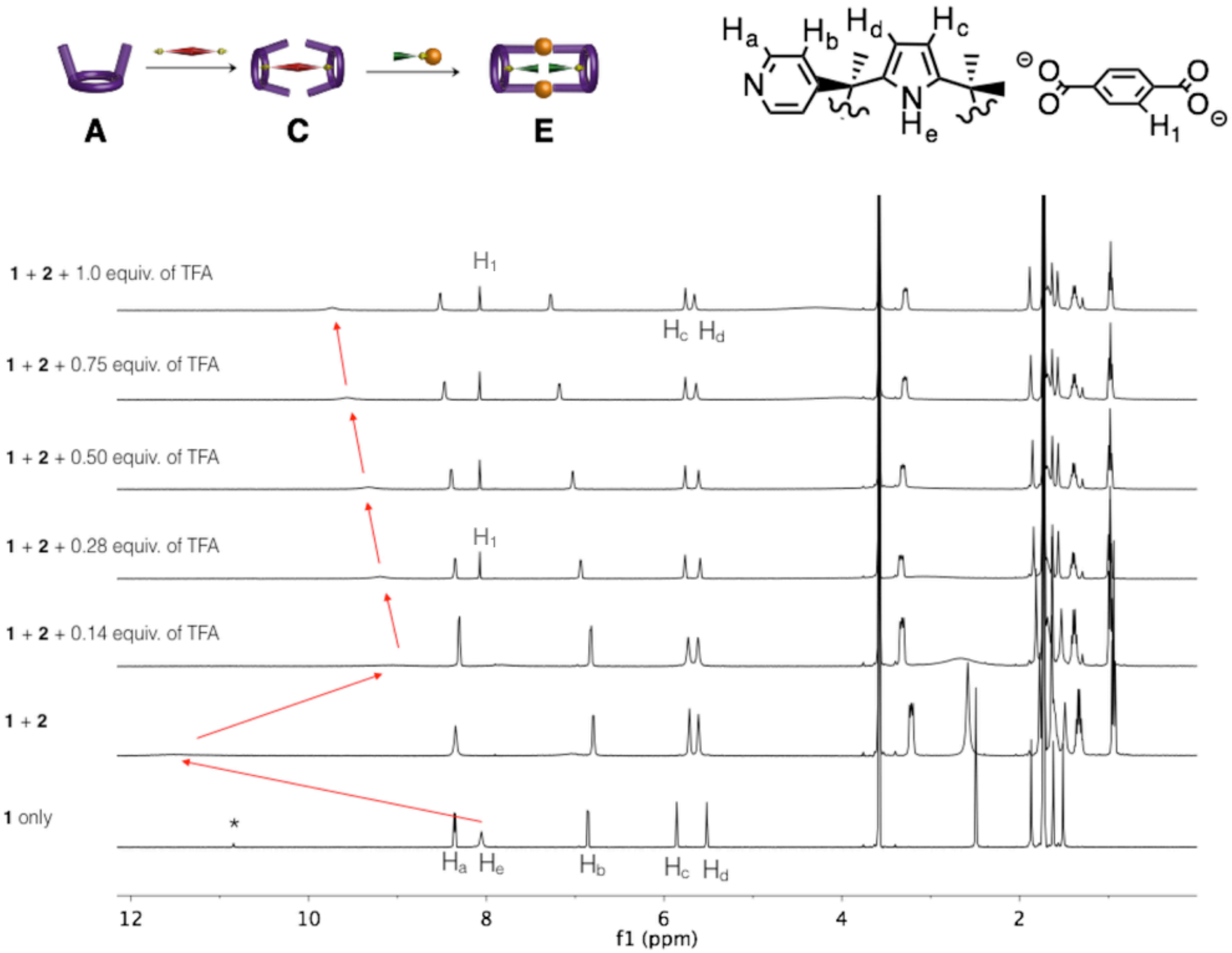

Supplementary Figure 37. Changes in the ^{1}H NMR spectrum of a 2:1 mixture of **1** (5.0×10^{-3} mol L^{-1}) and bisTBA salt of **2** (2.5×10^{-3} mol L^{-1}) seen upon the sequential addition of TFA. The spectra were recorded in THF-d_8. *Indicates solvent impurity.

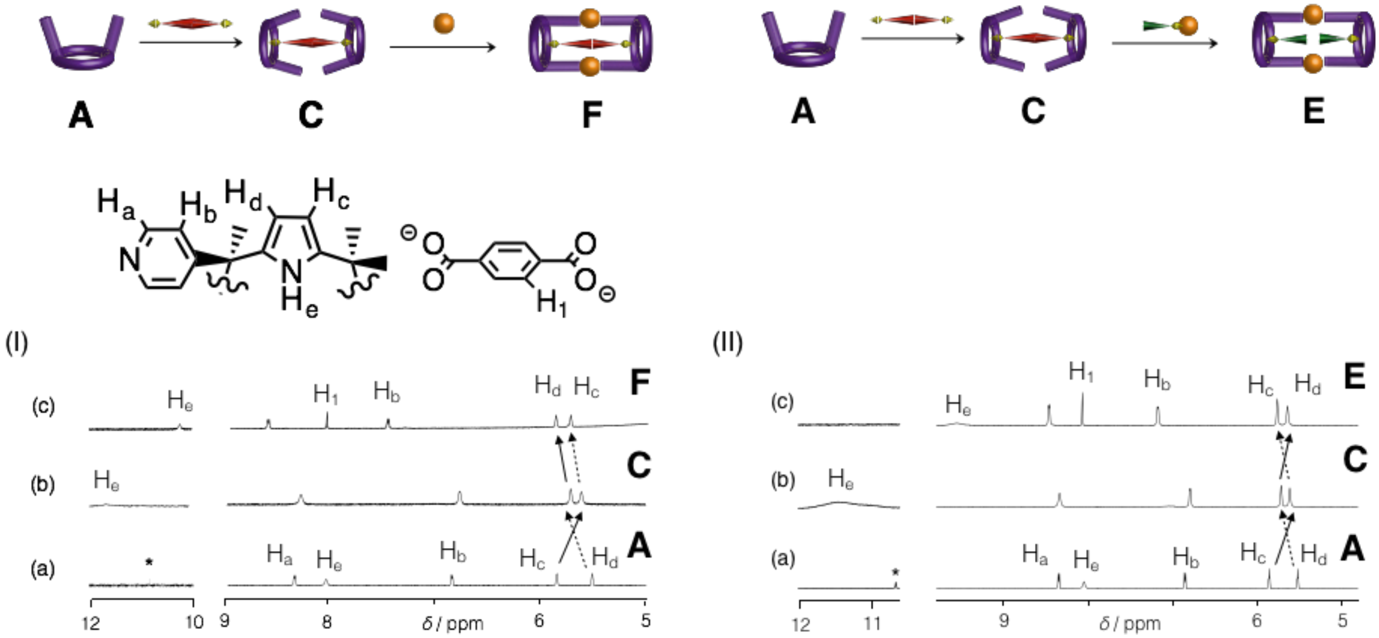

Supplementary Figure 38. (I) Changes in the ^1H NMR spectra of (a) **1** (2.0 x 10^{-3} mol L^{-1}) in THF-d_8 seen upon the sequential addition of (b) the bisTBA salt of **2** (0.5 equiv.), (c) 50% aqueous HBF_4 (1.0 equiv.). (II) Changes in the ^1H NMR spectra of (a) **1** (2.0 x 10^{-3} mol L^{-1}) in THF-d_8 seen upon the sequential addition of (b) the bisTBA salt of **2** (0.5 equiv.), and (c) TFA (1.0 equiv.). *Indicates solvent impurity.

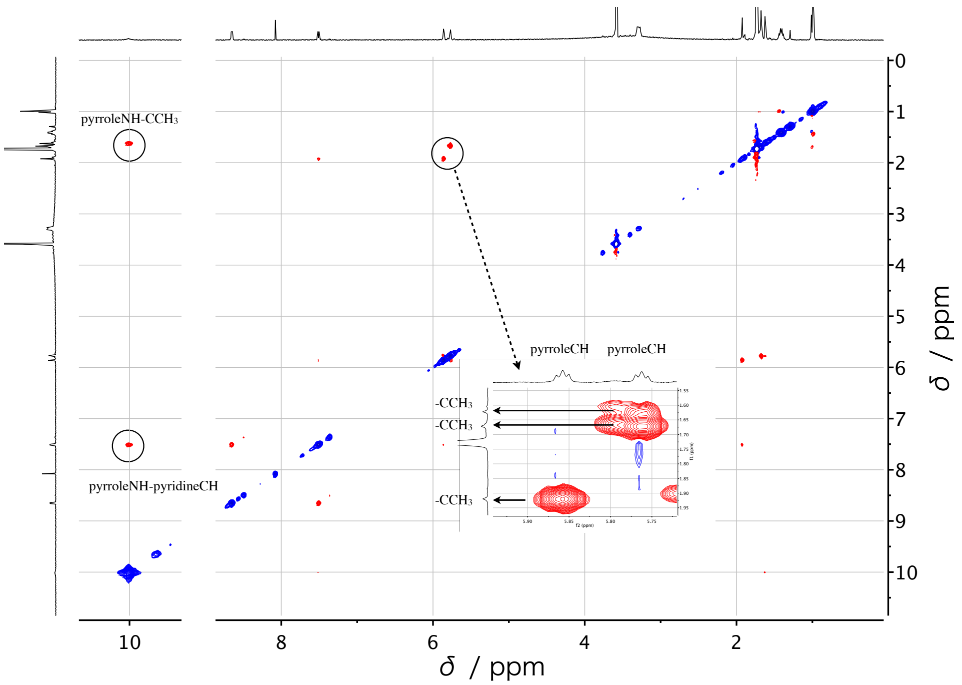

Supplementary Figure 39. NOESY spectrum of a 2:1:2 mixture of **1** (2.0 x 10^{-3} mol L^{-1}), the bisTBA salt of **2** (1.0 x 10^{-3} mol L^{-1}) and 50% aqueous HBF_4 (2.0 x 10^{-3} mol L^{-1}) in THF-d_8.

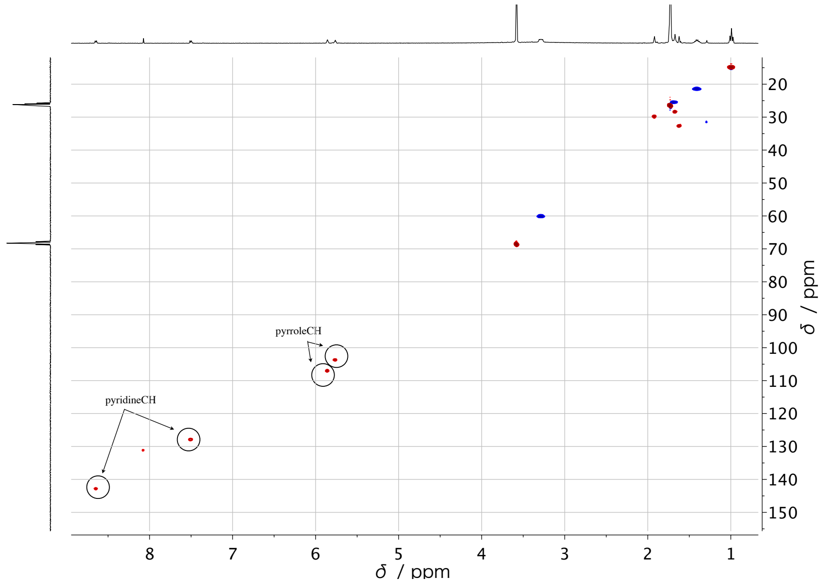

Supplementary Figure 40. HSQC spectrum of a 2:1:2 mixture of **1** (2.0 x 10^{-3} mol L^{-1}), the bisTBA salt of **2** (1.0 x 10^{-3} mol L^{-1}) and 50% aqueous HBF_4 (2.0 x 10^{-3} mol L^{-1}) in THF-d_8. CH_3 and CH carbons are phased up (red), and CH_2 carbons are phased down (blue).

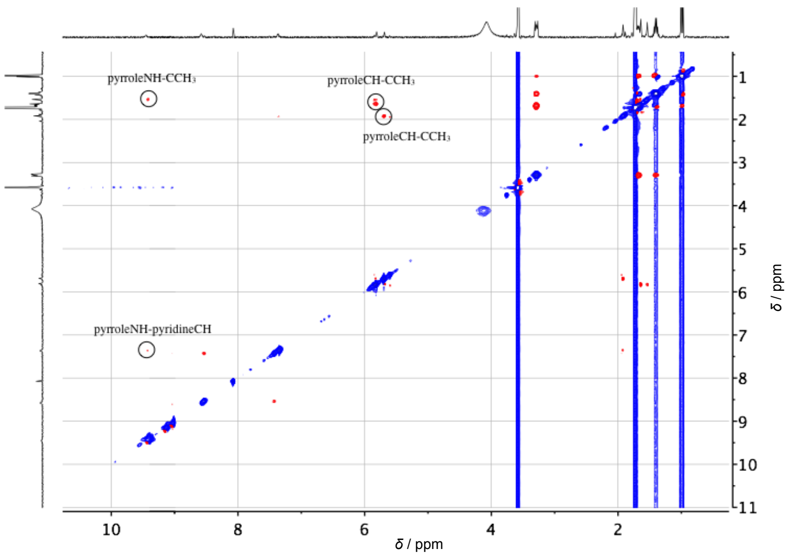

Supplementary Figure 41. NOESY spectrum of a 2:1:2 mixture of **1** (2.0×10^{-3} mol L^{-1}), the bisTBA salt of **2** (1.0×10^{-3} mol L^{-1}) and TFA (2.0×10^{-3} mol L^{-1}) in THF-d_8.

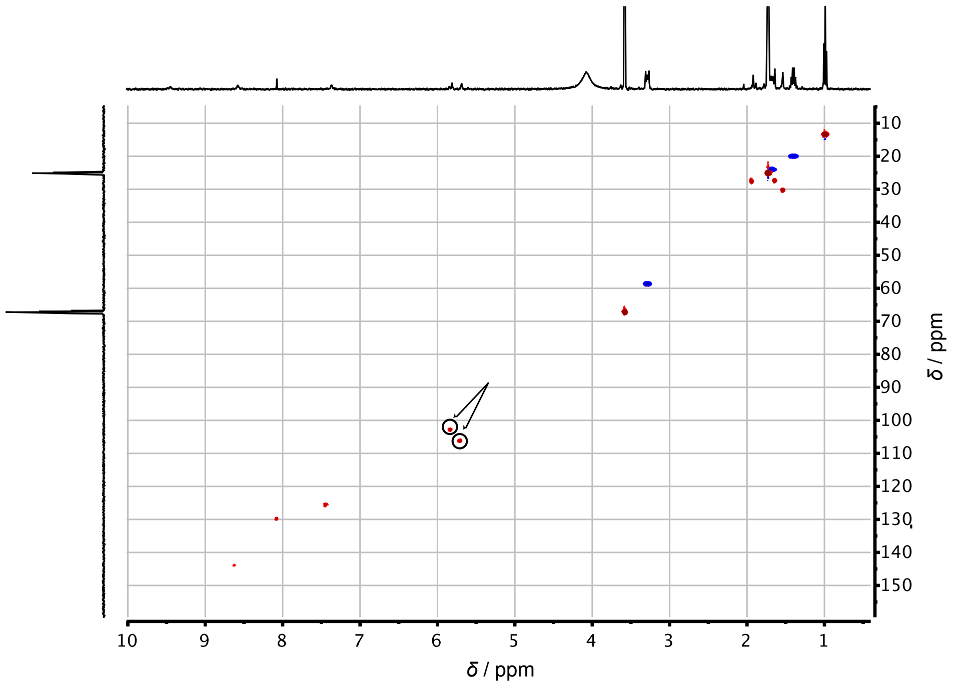

Supplementary Figure 42. HSQC spectrum of a 2:1:2 mixture of **1** (2.0×10^{-3} mol L^{-1}), the bisTBA salt of **2** (1.0×10^{-3} mol L^{-1}) and TFA (2.0×10^{-3} mol L^{-1}) in THF-d_8. CH_3 and CH carbons are phased up (red), and CH_2 carbons are phased down (blue).

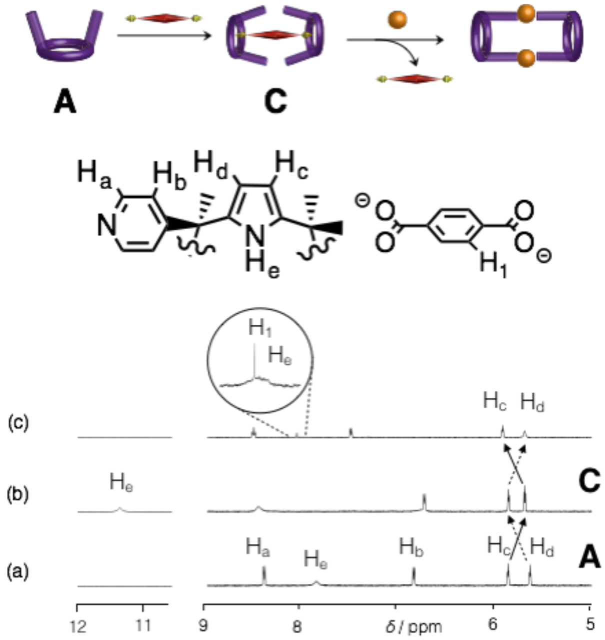

Supplementary Figure 43. Changes in the ^{1}H NMR spectra of (a) **1** (2.0 x 10^{-3} mol L^{-1}) in MeCN-d_3 seen upon the sequential addition of (b) the bisTBA salt of **2** (0.5 equiv.), (c) 50% aqueous HBF_4 (1.0 equiv.).

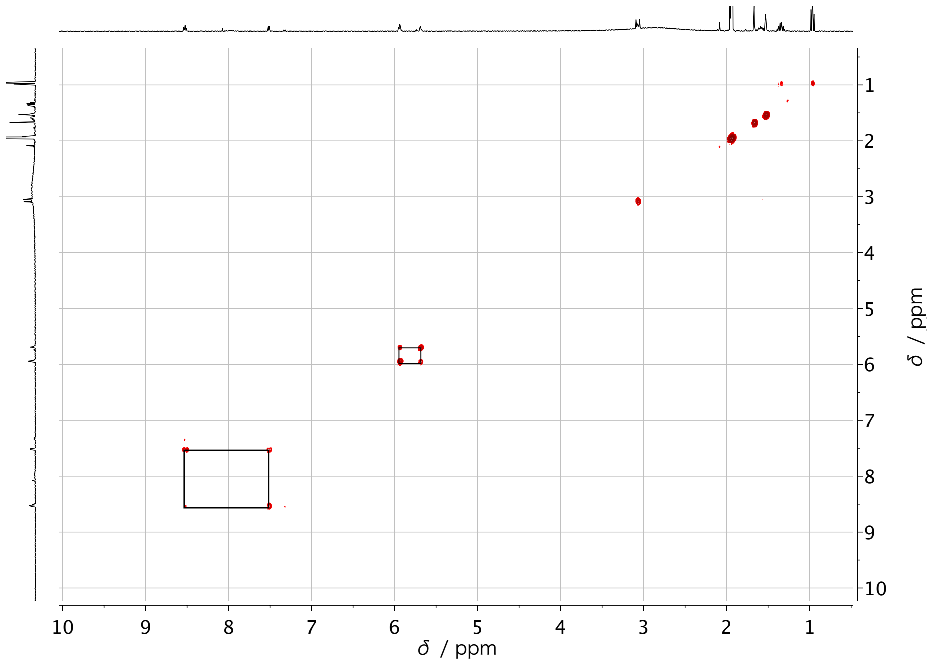

Supplementary Figure 44. COSY spectrum of a 2:1:2 mixture of **1** (2.0 x 10^{-3} mol L^{-1}), the bisTBA salt of **2** (1.0 x 10^{-3} mol L^{-1}), and 50% aqueous HBF_4 (2.0 x 10^{-3} mol L^{-1}) in MeCN-d_3.

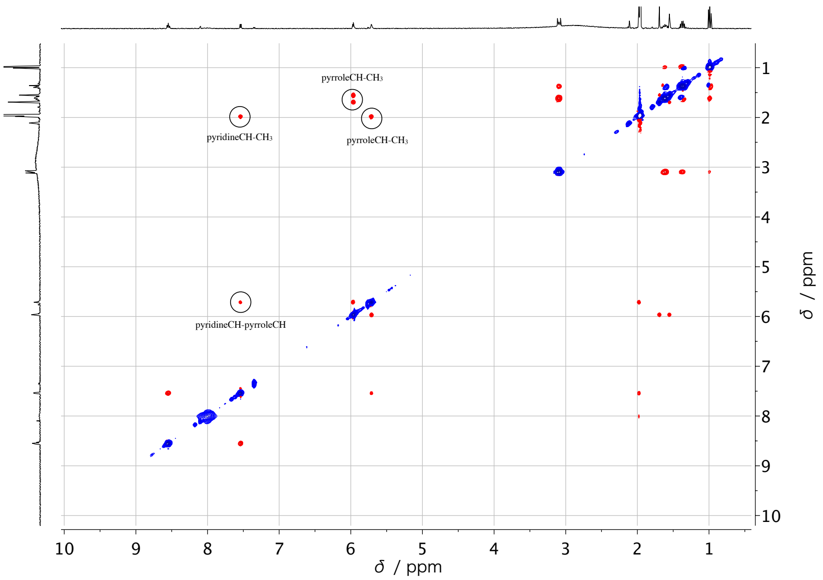

Supplementary Figure 45. NOESY spectrum of a 2:1:2 mixture of **1** (2.0×10^{-3} mol L^{-1}), the bisTBA salt of **2** (1.0×10^{-3} mol L^{-1}) and 50% aqueous HBF_4 (2.0×10^{-3} mol L^{-1}) in MeCN-d_3.

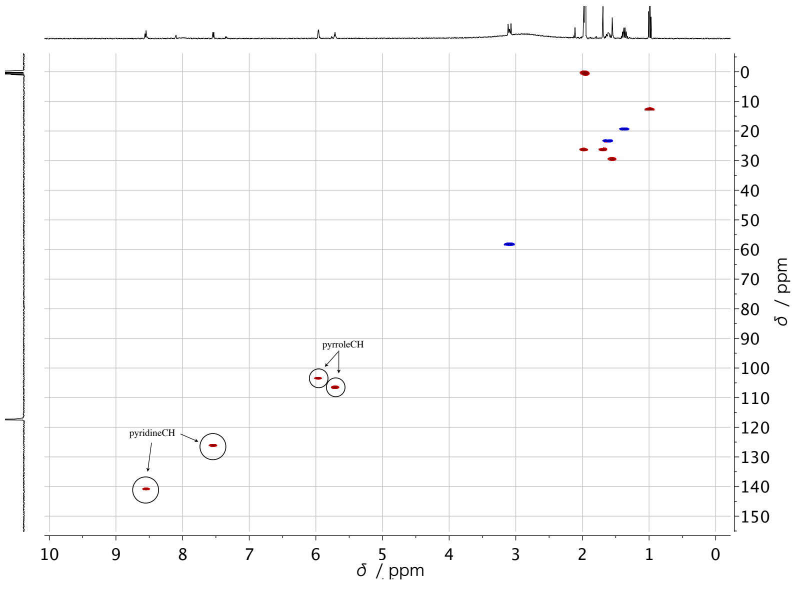

Supplementary Figure 46. HSQC spectrum of a 2:1:2 mixture of **1** (2.0×10^{-3} mol L^{-1}), the bisTBA salt of **2** (1.0×10^{-3} mol L^{-1}) and TFA (2.0×10^{-3} mol L^{-1}) in THF-d_8. CH_3 and CH carbons are phased up (red), and CH_2 carbons are phased down (blue).

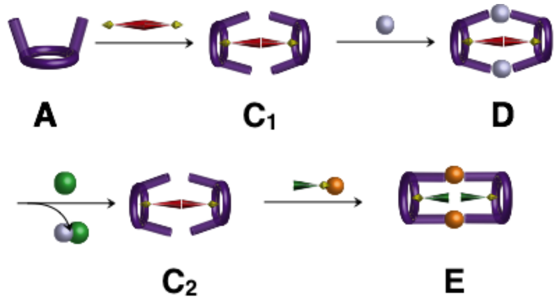

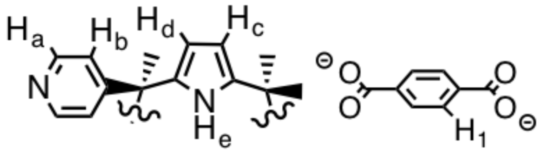

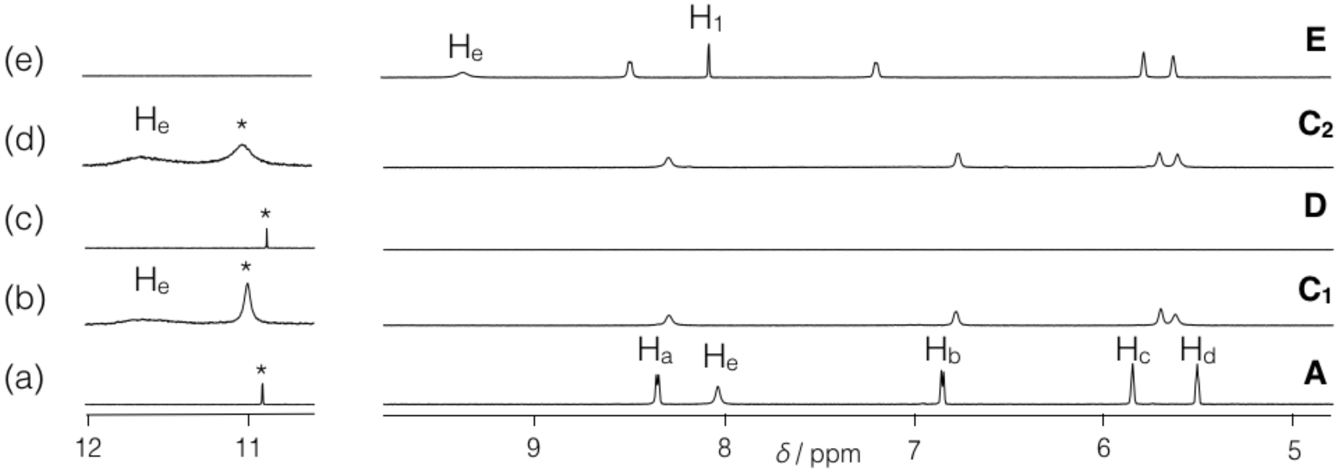

Supplementary Figure 47. Changes in the ^{1}H NMR spectrum of (a) **1** (8.0×10^{-4} mol L^{-1}) seen upon the sequential addition of (b) the bisTBA salt of **2** (0.5 equiv.), (c) $AgBF_4$ (1.0 equiv.), (d) TBACl (1.0 equiv.), and (e) TFA (1.0 equiv.). The spectra were recorded in THF-d_8. *Indicates solvent impurity.

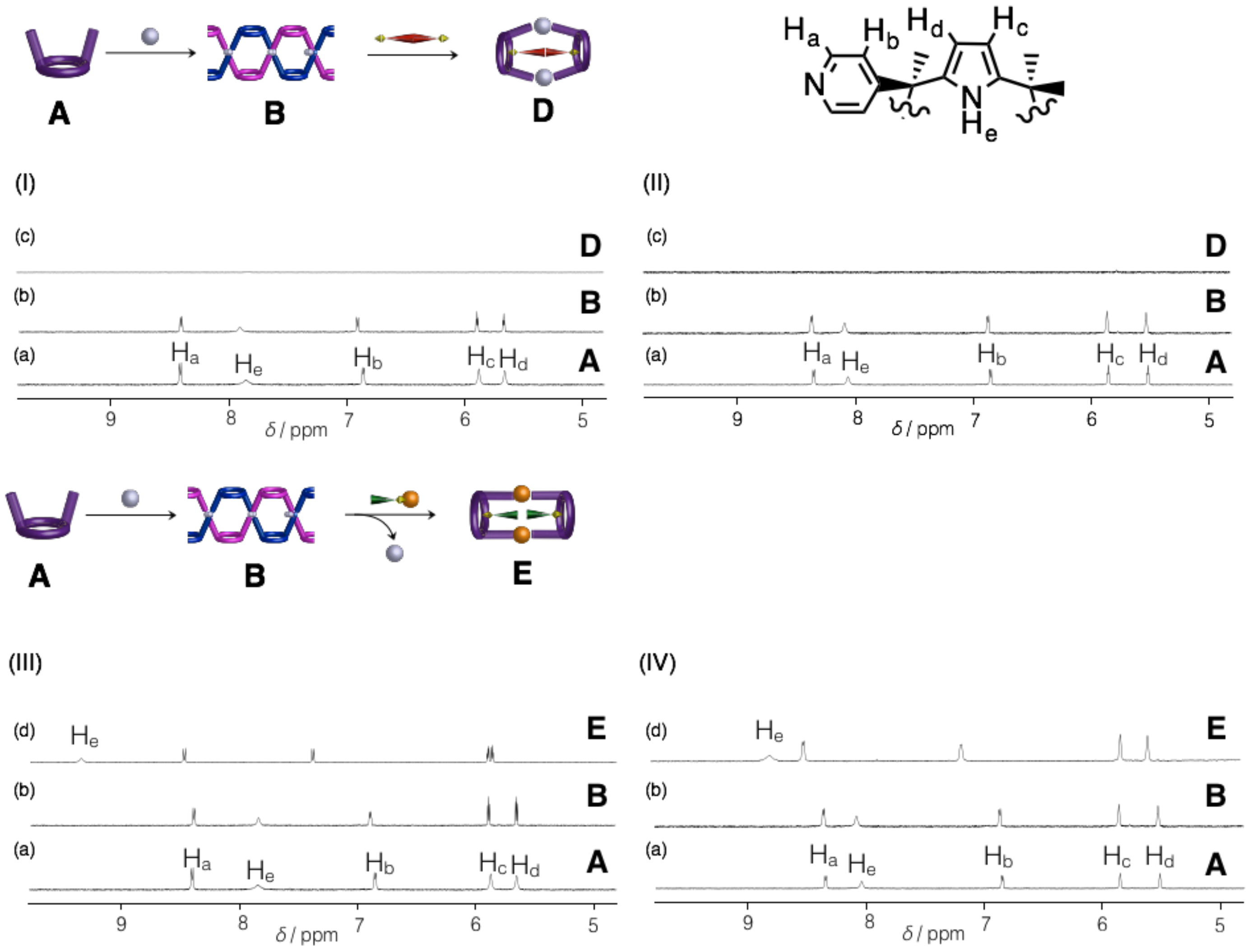

Supplementary Figure 48. Changes in the ^{1}H NMR spectra of (I-a, III-a) **1** (2.0×10^{-3} mol L^{-1}) in MeCN-d_3 and (II-a, IV-a) **1** (8.0×10^{-4} mol L^{-1}) in THF-d_8 seen upon the sequential addition of (I-b, II-b, III-b, IV-b) $AgBF_4$ (1.0 equiv.), (I-c, II-c) the bisTBA salt of **2** (0.5 equiv.), and (III-d, IV-d) TFA (1.0 equiv.).

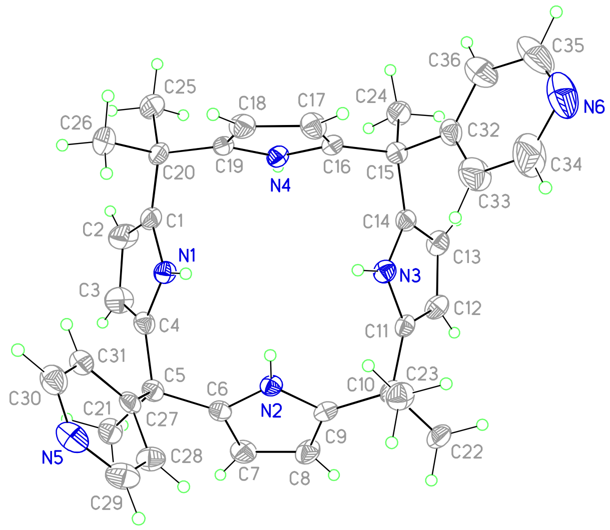

Supplementary Figure 49. View of **1** showing the atom labeling scheme. Displacement ellipsoids are scaled to the 50% probability level.

Supplementary Note 4.

X-ray Experimental for $C_{36}H_{38}N_6$ – 3/2 CH_3CN – H_2O: Crystals grew as clusters of colorless prisms by slow evaporation from a 1:1 mixture of chloroform and acetonitrile. The data crystal was cut from a larger crystal and had approximate dimensions; 0.29 x 0.14 x 0.10 mm. The data were collected on a Rigaku SCX-Mini diffractometer with a Mercury 2 CCD using a graphite monochromator with MoKα radiation (λ = 0.71075 Å). A total of 1080 frames of data were collected using ω-scans with a scan range of 0.5° and a counting time of 26 seconds per frame. The data were collected at 163 K using a Rigaku XStream low temperature device. Details of crystal data, data collection and structure refinement are listed in Supplementary Table 2. Data reduction were performed using the Rigaku Americas Corporation's Crystal Clear version 1.40.[5] The structure was solved by direct methods using SIR97[6] and refined by full-matrix least-squares on F^2 with anisotropic displacement parameters for the non-H atoms using SHELXL-97.[7] Structure analysis was aided by use of the programs PLATON98[8] and WinGX.[9] The hydrogen atoms on carbon were calculated in ideal positions with isotropic displacement parameters set to 1.2xUeq of the attached atom (1.5xUeq for methyl hydrogen atoms). The hydrogen atoms on the water molecule were observed in a ΔF map and refined with isotropic displacement parameters.

One of the acetonitrile molecules was disordered about a crystallographic two-fold rotation axis at 1, 1, ¾. The function, $\Sigma w(|F_o|^2 - |F_c|^2)^2$, was minimized, where $w = 1/[(\sigma(F_o))^2 + (0.0356*P)^2 + (4.1109*P)]$ and $P = (|F_o|^2 + 2|F_c|^2)/3$. $R_w(F^2)$ refined to 0.132, with R(F) equal to 0.0656 and a goodness of fit, S, = 1.15. Definitions used for calculating R(F), $R_w(F^2)$ and the goodness of fit, S, are given as $R_W(F^2) = \{\Sigma w(|F_O|^2 - |F_C|^2)^2/\Sigma w(|F_O|)^4\}^{1/2}$ where w is the weight given each

reflection, $R(F) = \Sigma(|F_o| - |F_c|)/\Sigma|F_o|\}$ for reflections with $F_o > 4(\sigma(F_o))$, and $\Sigma = [\Sigma w(|F_o|^2 - |F_c|^2)^2/(n - p)]^{1/2}$, where n is the number of reflections and p is the number of refined parameters. The data were checked for secondary extinction but no correction was necessary. Neutral atom scattering factors and values used to calculate the linear absorption coefficient are from the International Tables for X-ray Crystallography (1992).[10] All figures were generated using SHELXTL/PC.[11]

Empirical formula	C39 H44.50 N7.50 O	
Formula weight	634.32	
Temperature	163(2) K	
Wavelength	0.71075 Å	
Crystal system	Monoclinic	
Space group	C2/c	
Unit cell dimensions	a = 31.768(6) Å	α = 90°.
	b = 10.7312(19) Å	β = 103.448(5)°.
	c = 20.889(4) Å	γ = 90°.
Volume	6926(2) $Å^3$	
Z	8	
Density (calculated)	1.217 Mg/m3	
Absorption coefficient	0.076 mm-1	
F(000)	2712	
Crystal size	0.29 x 0.14 x 0.10 mm	
Theta range for data collection	3.07 to 27.48°.	
Index ranges	-41<=h<=39, -13<=k<=13, -27<=l<=27	
Reflections collected	34373	
Independent reflections	7926 [R(int) = 0.0857]	
Completeness to theta = 27.48°	99.8 %	
Absorption correction	Semi-empirical from equivalents	
Max. and min. transmission	1.00 and 0.841	
Refinement method	Full-matrix least-squares on F2	
Data / restraints / parameters	7926 / 21 / 474	
Goodness-of-fit on F2	1.146	
Final R indices [I>2sigma(I)]	R1 = 0.0656, wR2 = 0.1137	
R indices (all data)	R1 = 0.1224, wR2 = 0.1320	
Largest diff. peak and hole	0.226 and -0.242 e.Å-3	
CCDC #	1058558	

Supplementary Table 2. Crystal data and structure refinement for **1**.

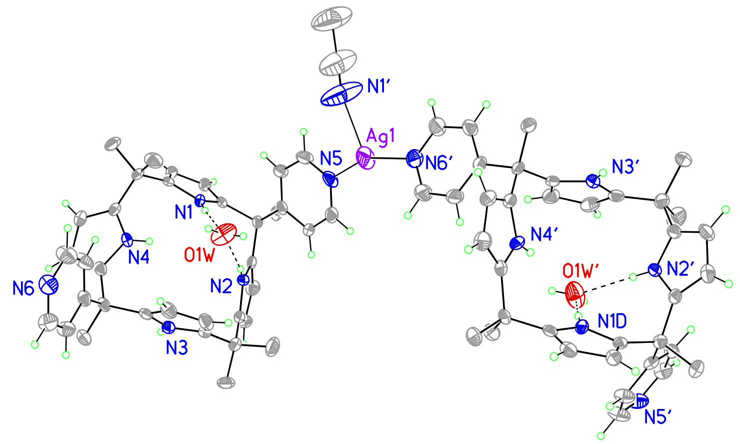

Supplementary Figure 50. View of [**1**•Ag]$_\infty$ showing the heteroatom labeling scheme. Displacement ellipsoids are scaled to the 50% probability level.

Supplementary Note 5.

X-ray Experimental for $(C_{36}H_{38}N_6)Ag(BF_4)$ – H_2O – $3CH_3CN$: Crystals grew as yellow plates and prisms by slow evaporation from acetonitrile. The data crystal was cut from a larger crystal and had approximate dimensions; 0.34 x 0.22 x 0.09 mm. The data were collected at -140 °C on a Nonius Kappa CCD diffractometer using a Bruker AXS Apex II detector and a graphite monochromator with MoKα radiation (λ = 0.71073 Å). Reduced temperatures were maintained by use of an Oxford Cryosystems 600 low-temperature device. A total of 1836 frames of data were collected using ω and ϕ-scans with a scan range of 0.5° and a counting time of 30 seconds per frame. Details of crystal data, data collection and structure refinement are listed in Supplementary Table 3. Data reduction were performed using SAINT V8.27B.[12] The structure was solved by direct methods using SUPERFLIP[13] and refined by full-matrix least-squares on F^2 with anisotropic displacement parameters for the non-H atoms using SHELXL-2013.[7] Structure analysis was aided by use of the programs PLATON98[8] and WinGX.[9] The hydrogen atoms were calculated in idealized positions. The tetrafluoroborate anion was disordered across a crystallographic two-fold rotation axis along ½, y, ¾. Additionally, a molecule of acetonitrile was also disordered about two orientations of approximately 50% occupancy.

The function, $\Sigma w(|F_o|^2 - |F_c|^2)^2$, was minimized, where $w = 1/[(\sigma(F_o))^2 + (0.0607*P)^2 + (9.9 *P)]$ and $P = (|F_o|^2 + 2|F_c|^2)/3$. $R_w(F^2)$ refined to 0.151, with R(F) equal to 0.0565 and a goodness of fit, S, = 1.10. Definitions used for calculating R(F), $R_w(F^2)$ and the goodness of fit, S, are given as $R_W(F^2) = \{\Sigma w(|F_O|^2 - |F_C|^2)^2/\Sigma w(|F_O|)^4\}^{1/2}$ where w is the weight given each reflection, $R(F) = \Sigma(|F_O| - |F_C|)/\Sigma|F_O|\}$ for reflections with $F_O > 4(\sigma(F_O))$, and $\Sigma = [\Sigma w(|F_O|^2 - |F_C|^2)^2/(n - p)]^{1/2}$, where n is

the number of reflections and p is the number of refined parameters.

The data were checked for secondary extinction but no correction was necessary. Neutral atom scattering factors and values used to calculate the linear absorption coefficient are from the International Tables for X-ray Crystallography (1992).[10] All figures were generated using SHELXTL/PC.[11]

Empirical formula	C42 H49 Ag B F4 N9 O	
Formula weight	890.58	
Temperature	133(2) K	
Wavelength	0.71073 Å	
Crystal system	monoclinic	
Space group	P 2/c	
Unit cell dimensions	a = 19.692(2) Å	α = 90°.
	b = 10.7710(12) Å	β = 107.186(4)°.
	c = 20.408(3) Å	γ = 90°.
Volume	4135.3(9) $Å^3$	
Z	4	
Density (calculated)	1.430 Mg/m^3	
Absorption coefficient	0.551 mm^{-1}	
F(000)	1840	
Crystal size	0.340 x 0.220 x 0.090 mm	
Theta range for data collection	2.049 to 25.072°.	
Index ranges	-23<=h<=23, -12<=k<=12, -24<=l<=24	
Reflections collected	67745	
Independent reflections	7341 [R(int) = 0.0761]	
Completeness to theta = 25.242°	98.0 %	
Absorption correction	Semi-empirical from equivalents	
Max. and min. transmission	1.00 and 0.840	
Refinement method	Full-matrix least-squares on F^2	
Data / restraints / parameters	7341 / 154 / 574	
Goodness-of-fit on F^2	1.079	
Final R indices [I>2sigma(I)]	R1 = 0.0565, wR2 = 0.1370	
R indices (all data)	R1 = 0.0793, wR2 = 0.1510	
Extinction coefficient	n/a	
Largest diff. peak and hole	0.829 and -0.810 e.$Å^{-3}$	
CCDC #	1058559	

Supplementary Table 3. Crystal data and structure refinement for [**1**•Ag]$_\infty$.

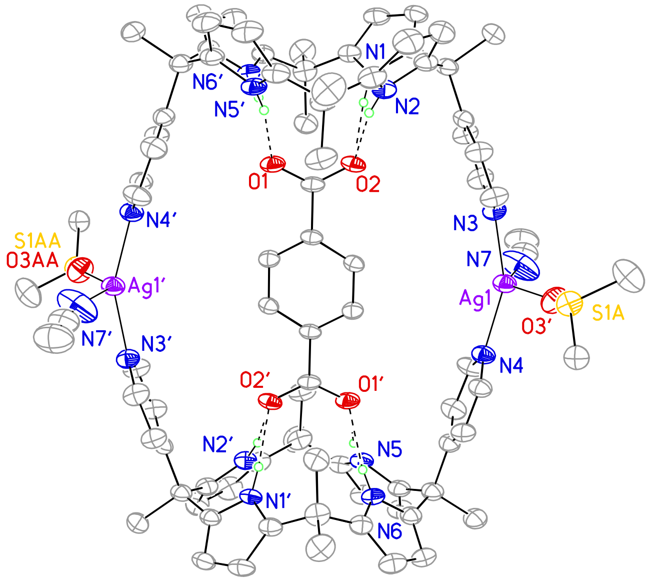

Supplementary Figure 51. View of [Ag_2•$\mathbf{1}_2$•terephthalate] showing the heteroatom labeling scheme. Displacement ellipsoids are scaled to the 40% probability level. The complex resides around a crystallographic inversion center at ½, ½, ½. Atoms with labels appended by a ' are related by 1-x, 1-y, 1-z.

Supplementary Note 6.

X-ray Experimental for [$(C_{36}H_{38}N_4)_2(C_8H_4O_4)^{2-}$ $2Ag^{1+}$] - 4 C_2H_6SO – 2 C_2H_3N: Crystals grew as clusters of hexagonal shaped colorless prisms by slow evaporation from methanol, acetonitrile and DMSO. The data crystal had approximate dimensions; 0.20 x 0.12 x 0.09 mm. The data were collected on an Agilent Technologies SuperNova Dual Source diffractometer using a μ-focus Cu Kα radiation source (λ = 1.5418 Å) with collimating mirror monochromators. A total of 742 frames of data were collected using ω-scans with a scan range of 1° and a counting time of 22.5 seconds per frame with a detector offset at +/- 41° and a counting time of 55 seconds for frames collected with a detector offset of +/- 108°. The data were collected at 133 K using an Oxford Cryostream low temperature device. Details of crystal data, data collection and structure refinement are listed in Supplementary Table 4. Data collection, unit cell refinement and data reduction were performed using Agilent Technologies CrysAlisPro V 1.171.37.31.[14] The structure was solved by direct methods using SIR97[5] and refined by full-matrix least-squares on F^2 with anisotropic displacement parameters for the non-H atoms using SHELXL-2013.[7] Structure analysis was aided by use of the programs PLATON98[8] and WinGX.[9] The hydrogen atoms on carbon were calculated in ideal positions with isotropic displacement parameters set to 1.2xUeq of the attached atom (1.5xUeq for methyl hydrogen atoms). The hydrogen atoms bound to nitrogen were located in a ΔF map and refined with isotropic displacement parameters.

The complex resides around a crystallographic inversion center at 1/2, 1/2, 1/2. Both DMSO molecules were disordered. The disorder was modeled in the same manner for both molecules. For one DMSO molecule, the variable x was assigned to the site occupancy factors for the atoms in one component of the disorder. The variable (1-x) was assigned to the site occupancy factors for the alternate component. A common isotropic displacement parameter was refined while refining the variable x. The geometry of the DMSO molecules was restrained to be equivalent throughout the refinement process. In this way, the major component for one DMSO molecule refined to 55(2)%, while the major component for the second DMSO molecule refined to 73(2)%.

The function, $\Sigma w(|F_o|^2 - |F_c|^2)^2$, was minimized, where $w = 1/[(\sigma(F_o))^2 + (0.0857*P)^2 + (12.6415*P)]$ and $P = (|F_o|^2 + 2|F_c|^2)/3$. $R_w(F^2)$ refined to 0.198, with R(F) equal to 0.0692 and a goodness of fit, S, = 1.16. Definitions used for calculating R(F), $R_w(F^2)$ and the goodness of fit, S, are given as $R_W(F^2) = \{\Sigma w(|F_O|^2 - |F_C|^2)^2/\Sigma w(|F_O|)^4\}^{1/2}$ where w is the weight given each reflection, $R(F) = \Sigma(|F_O| - |F_C|)/\Sigma|F_O|\}$ for reflections with $F_O > 4(\sigma(F_O))$, and $\Sigma = [\Sigma w(|F_O|^2 - |F_C|^2)^2/(n - p)]^{1/2}$, where n is the number of reflections and p is the number of refined parameters. The data were checked for secondary extinction effects but no correction was necessary. Neutral atom scattering factors and values used to calculate the linear absorption coefficient are from the International Tables for X-ray Crystallography (1992).[10] All figures were generated using SHELXTL/PC.[11]

Empirical formula	C92 H110 Ag2 N14 O8 S4	
Formula weight	1883.91	
Temperature	133(2) K	
Wavelength	1.54184 Å	
Crystal system	monoclinic	
Space group	P 21/n	
Unit cell dimensions	a = 15.6125(9) Å	α = 90°.
	b = 14.9625(10) Å	β = 110.917(6)°.
	c = 20.6736(9) Å	γ = 90°.
Volume	4511.1(5) $Å^3$	
Z	2	
Density (calculated)	1.387 Mg/m^3	
Absorption coefficient	4.854 mm^{-1}	
F(000)	1964	
Crystal size	0.200 x 0.120 x 0.090 mm	
Theta range for data collection	3.737 to 69.985°.	
Index ranges	-18<=h<=19, -15<=k<=18, -25<=l<=17	
Reflections collected	17684	
Independent reflections	8509 [R(int) = 0.0377]	
Completeness to theta = 67.684°	99.9 %	
Absorption correction	Semi-empirical from equivalents	
Max. and min. transmission	1.00 and 0.566	
Refinement method	Full-matrix least-squares on F^2	
Data / restraints / parameters	8509 / 249 / 634	
Goodness-of-fit on F^2	1.133	
Final R indices [I>2sigma(I)]	R1 = 0.0692, wR2 = 0.1809	
R indices (all data)	R1 = 0.0840, wR2 = 0.1982	
Extinction coefficient	n/a	
Largest diff. peak and hole	1.490 and -1.028 e.$Å^{-3}$	
CCDC #	1058560	

Supplementary Table 4. Crystal data and structure refinement for [Ag_2•$\mathbf{1}_2$•terephthalate].

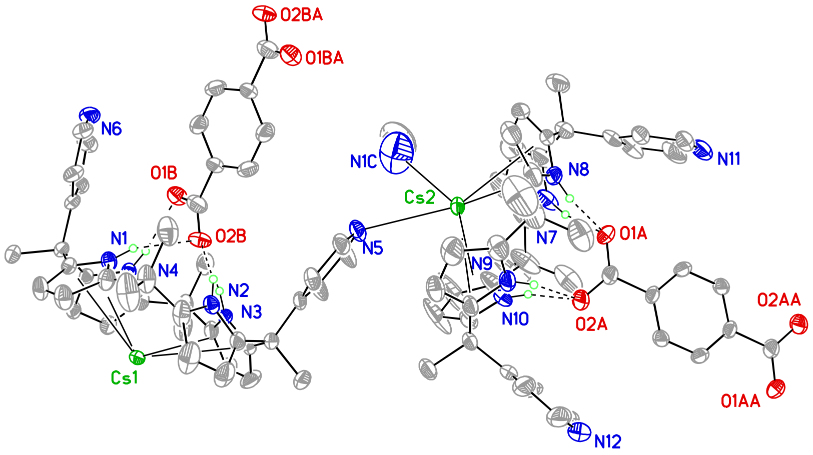

Supplementary Figure 52. View of [Cs_2•$\mathbf{1}_2$•terephthalate] showing a partial atom labeling scheme. Displacement ellipsoids are scaled to the 50% probability level.

Supplementary Note 7.

X-ray Experimental for [($C_{36}H_{38}N_6$)Cs]$_2$ $C_8H_4O_4$ - 3CH_3N: Crystals grew as colorless prisms by slow evaporation from a 1:1 mixture of chloroform and acetonitrile. The data crystal had approximate dimensions; 0.17 x 0.13 x 0.04 mm. The data were collected on an Agilent Technologies SuperNova Dual Source diffractometer using a μ-focus Cu Kα radiation source (λ = 1.5418 Å) with collimating mirror monochromators. A total of 2284 frames of data were collected using w-scans with a scan range of 2° and a counting time of 30 seconds per frame with a detector offset of +/- 40.5° and 90 seconds per frame with a detector offset of +/- 109.9°. The data were collected at 100 K using an Oxford Cryostream low temperature device. Details of crystal data, data collection and structure refinement are listed in Supplementary Table 5. Data collection, unit cell refinement and data reduction were performed using Agilent Technologies CrysAlisPro V 1.171.37.31. The structure was solved by direct methods using SIR97[6] and refined by full-matrix least-squares on F^2 with anisotropic displacement parameters for the non-H atoms using SHELXL-2013.[7] Structure analysis was aided by use of the programs PLATON98[8] and WinGX.[9] The hydrogen atoms were calculated in ideal positions with isotropic displacement parameters set to 1.2xUeq of the attached atom (1.5xUeq for methyl hydrogen atoms). The hydrogen atoms bound to nitrogen were located in a ΔF map and refined with isotropic displacement parameters.

A molecule of acetonitrile was badly disordered about a crystallographic inversion center at 0, ½, 1. Attempts to model the disorder were unsatisfactory. The contributions to the scattering factors due to the solvent molecule were removed by use of the utility SQUEEZE[15] in PLATON98.

The function, $\Sigma w(|F_o|^2 - |F_c|^2)^2$, was minimized, where $w = 1/[(\sigma(F_o))^2 + (0.0496*P)^2 + (11.5937*P)]$ and $P = (|F_o|^2 + 2|F_c|^2)/3$. $R_w(F^2)$ refined to 0.118, with R(F) equal to 0.0462 and a goodness of fit, S, = 1.02. Definitions used for calculating R(F), $R_w(F^2)$ and the goodness of fit, S, are given below.[9] The data were checked for secondary extinction effects but no correction was necessary. Neutral atom scattering factors and values used to calculate the linear absorption coefficient are from the International Tables for X-ray Crystallography (1992).[10] All figures were generated using SHELXTL/PC.[11]

Empirical formula	C86 H89 Cs2 N15 O4	
Formula weight	1662.54	
Temperature	100(2) K	
Wavelength	1.54184 Å	
Crystal system	triclinic	
Space group	P -1	
Unit cell dimensions	a = 11.2373(11) Å	α = 79.728(5)°.
	b = 18.3845(8) Å	β = 77.533(8)°.
	c = 20.4193(15) Å	γ = 88.574(6)°.
Volume	4052.6(5) $Å^3$	
Z	2	
Density (calculated)	1.362 Mg/m^3	
Absorption coefficient	7.482 mm^{-1}	
F(000)	1704	
Crystal size	0.170 x 0.130 x 0.040 mm	
Theta range for data collection	3.015 to 76.964°.	
Index ranges	-14<=h<=14, -22<=k<=23, -25<=l<=25	
Reflections collected	113961	
Independent reflections	16829 [R(int) = 0.0532]	
Completeness to theta = 67.684°	100.0 %	
Absorption correction	Semi-empirical from equivalents	
Max. and min. transmission	1.00 and 0.555	
Refinement method	Full-matrix least-squares on F^2	
Data / restraints / parameters	16829 / 125 / 998	
Goodness-of-fit on F^2	0.982	
Final R indices [I>2sigma(I)]	R1 = 0.0462, wR2 = 0.1150	
R indices (all data)	R1 = 0.0498, wR2 = 0.1177	
Extinction coefficient	n/a	
Largest diff. peak and hole	1.485 and -0.892 e.$Å^{-3}$	
CCDC #	1058561	

Supplementary Table 5. Crystal data and structure refinement for [Cs_2•$\mathbf{1}_2$•terephthalate].

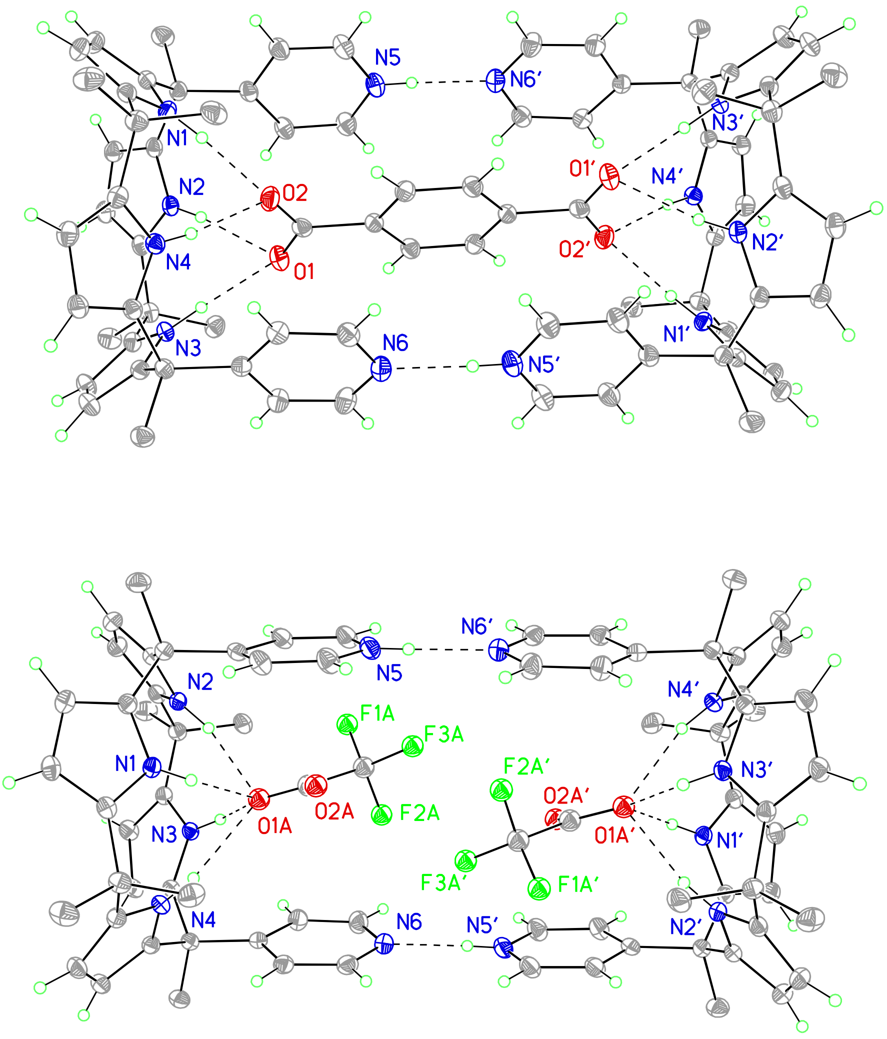

Supplementary Figure 53. Views of [H_2•**1**$_2$•terephthalate] (top) and [H_2•**1**$_2$•trifluoroacetate] (bottom) showing a partial atom labeling scheme. Displacement ellipsoids are scaled to the 50% probability level.

Supplementary Note 8.

X-ray Experimental for complex 2 ($C_{36}H_{39}N_6$) 0.93($C_8H_4O_4$) 0.07 ($C_2F_3O_2$) 4 C_2H_3N: Crystals grew as colorless prisms by evaporation from acetonitrile. The data crystal had approximate dimensions; 0.29 x 0.15 x 0.07 mm. The data were collected on an Agilent Technologies SuperNova Dual Source diffractometer using a μ-focus Cu Kα radiation source (λ =

1.5418 Å) with collimating mirror monochromators. A total of 965 frames of data were collected using ω-scans with a scan range of 1° and a counting time of 2 seconds per frame with a detector offset of +/- 40.5° and 5 seconds per frame with a detector offset of +/- 111°. The data were collected at 100 K using an Oxford Cryostream low temperature device. Details of crystal data, data collection and structure refinement are listed in Supplementary Table 6. Data collection, unit cell refinement and data reduction were performed using Agilent Technologies CrysAlisPro V 1.171.37.31.[14] The structure was solved by direct methods using SuperFlip[12] and refined by full-matrix least-squares on F^2 with anisotropic displacement parameters for the non-H atoms using SHELXL-2013.[7] Structure analysis was aided by use of the programs PLATON98[8] and WinGX.[9] The hydrogen atoms on carbon were calculated in ideal positions with isotropic displacement parameters set to 1.2xUeq of the attached atom (1.5xUeq for methyl hydrogen atoms). The hydrogen atoms on the pyrrole nitrogen atoms were observed in a ΔF map and refined with isotropic displacement parameters.

The anion encapsulated in the calixpyrrole dimer was composed of both terephthalate and trifluoroacetate ions. The trifluoroacetate ion is present in a small quantity of approximately 7%.

The function, $\Sigma w(|F_o|^2 - |F_c|^2)^2$, was minimized, where $w = 1/[(\sigma(F_o))^2 + (0.0446*P)^2 + (1.864*P)]$ and $P = (|F_o|^2 + 2|F_c|^2)/3$. $R_w(F^2)$ refined to 0.102, with R(F) equal to 0.0401 and a goodness of fit, S, = 1.02. Definitions used for calculating R(F), $R_w(F^2)$ and the goodness of fit, S, are given as $R_W(F^2) = \{\Sigma w(|F_O|^2 - |F_C|^2)^2/\Sigma w(|F_O|)^4\}^{1/2}$ where w is the weight given each reflection, $R(F) = \Sigma(|F_O| - |F_C|)/\Sigma|F_O|\}$ for reflections with $F_O > 4(\sigma(F_O))$, and $\Sigma = [\Sigma w(|F_O|^2 - |F_C|^2)^2/(n - p)]^{1/2}$, where n is the number of reflections and p is the number of refined parameters. The data were checked for secondary extinction effects but no correction was necessary. Neutral atom scattering factors and values used to calculate the linear absorption coefficient are from the International Tables for X-ray Crystallography (1992).[10] All figures were generated using SHELXTL/PC.[11]

Empirical formula	C87.50 H93.50 F0.50 N16 O4	
Formula weight	1442.78	
Temperature	100(2) K	
Wavelength	1.54184 Å	
Crystal system	monoclinic	
Space group	P 21/n	
Unit cell dimensions	a = 15.1217(4) Å	α = 90°.
	b = 15.2911(3) Å	β = 99.493(2)°.
	c = 16.9007(3) Å	γ = 90°.
Volume	3854.39(15) Å3	
Z	2	
Density (calculated)	1.243 Mg/m3	
Absorption coefficient	0.631 mm-1	
F(000)	1534	
Crystal size	0.290 x 0.150 x 0.068 mm	
Theta range for data collection	3.636 to 76.354°.	
Index ranges	-19<=h<=18, -18<=k<=19, -20<=l<=17	
Reflections collected	22578	
Independent reflections	7834 [R(int) = 0.0238]	
Completeness to theta = 67.684°	99.6 %	
Absorption correction	Semi-empirical from equivalents	
Max. and min. transmission	1.00 and 0.954	
Refinement method	Full-matrix least-squares on F^2	
Data / restraints / parameters	7834 / 10 / 542	
Goodness-of-fit on F2	1.023	
Final R indices [I>2sigma(I)]	R1 = 0.0401, wR2 = 0.0973	
R indices (all data)	R1 = 0.0461, wR2 = 0.1015	
Extinction coefficient	n/a	
Largest diff. peak and hole	0.377 and -0.326 e.Å-3	
CCDC #	1558007	

Supplementary Table 6. Crystal data and structure refinement for [H_2•**1**$_2$•terephthalate] + [H_2•**1**$_2$•trifluoroacetate].

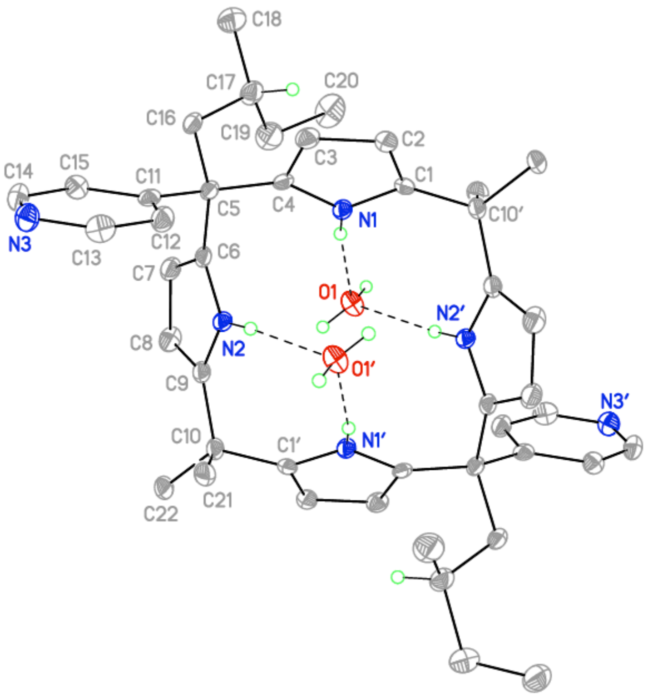

Supplementary Figure 54. View of the **9** showing the atom labeling scheme. Displacement ellipsoids are scaled to the 50% probability level.

Supplementary Note 9.

X-ray Experimental for $C_{44}H_{54}N_6 – 2\ H_2O$: Crystals grew as clear, colorless needles by slow evaporation from acetonitrile. The data crystal was cut from a larger crystal and had approximate dimensions; 0.31 x 0.15 x 0.11 mm. The data were collected at -150 °C on a Nonius Kappa CCD diffractometer using a Bruker AXS Apex II detector and a graphite monochromator with MoKα radiation (λ = 0.71073 Å). Reduced temperatures were maintained by use of an Oxford Cryosystems 600 low-temperature device. A total of 629 frames of data were collected using w and ϕ-scans with a scan range of 1.1° and a counting time of 85 seconds per frame. Details of crystal data, data collection and structure refinement are listed in Supplementary Table 7. Data reduction were performed using SAINT V8.27B.[12] The structure was solved by direct methods using SHELXT[11] and refined by full-matrix least-squares on F^2 with anisotropic displacement parameters for the non-H atoms using SHELXL-2014/7.[7] Structure analysis was aided by use of the programs PLATON98[8] and

WinGX.[9] The hydrogen atoms bound to carbon atoms were calculated in idealized positions. The hydrogen atoms on the water molecule were observed in a ΔF map and constrained in a riding model with Uiso set to 1.5xUeq of the water molecule, O3. The hydrogen atoms on the pyrrole nitrogen atoms were observed in a ΔF map and refined with isotropic displacement parameters.

The calixpyrrole di-hydrate complex resides around a crystallographic inversion center at ½, ½, ½. The pyrrole NH groups H-bond to the water molecule, which, in turn, is H-bound to the pyridine ring. The terminal methyl group of the 2-methylbutyl group is disordered. The disorder was modeled by assigning the variable x to the site occupancy factor for C20 and (1-x) was assigned to the site occupancy factor for the alternate component, C20a, of the disordered atom. A common isotropic displacement parameter was refined for both atoms while refining x. The site occupancy for C20 refined to 52(1)%. The function, $\Sigma w(|F_o|^2 - |F_c|^2)^2$, was minimized, where $w = 1/[(s(F_o))^2 + (5.5758*P)]$ and $P = (|F_o|^2 + 2|F_c|^2)/3$. $R_W(F^2)$ refined to 0.187, with R(F) equal to 0.0865 and a goodness of fit, S, = 1.21. Definitions used for calculating R(F),$R_W(F^2)$ and the goodness of fit, S, are given as $R_W(F^2) = \{\Sigma w(|F_o|^2 - |F_c|^2)^2/\Sigma w(|F_o|)^4\}^{1/2}$ where w is the weight given each reflection. $R_W(F^2) = \{\Sigma w(|F_o|^2 - |F_c|^2)^2/\Sigma w(|F_o|)^4\}^{1/2}$ where w is the weight given each reflection, $R(F) = \Sigma(|F_o| - |F_c|)/\Sigma|F_o|\}$ for reflections with $F_o > 4(\sigma(F_o))$, and $\Sigma = [\Sigma w(|F_o|^2 - |F_c|^2)^2/(n - p)]^{1/2}$, where n is the number of reflections and p is the number of refined parameters. The data were checked for secondary extinction but no correction was necessary. Neutral atom scattering factors and values used to calculate the linear absorption coefficient are from the International Tables for X-ray Crystallography (1992).[10] All figures were generated using SHELXTL/PC.[11] Tables of positional and thermal parameters, bond lengths and angles, torsion angles and figures may be obtained from the Cambridge Crystallographic Data Centre.

Empirical formula	C44 H58 N6 O2	
Formula weight	702.96	
Temperature	123(2) K	
Wavelength	0.71073 Å	
Crystal system	monoclinic	
Space group	P 21/n	
Unit cell dimensions	a = 10.6246(10) Å	α = 90°.
	b = 12.3223(13) Å	β = 98.778(6)°.
	c = 14.8745(14) Å	γ = 90°.
Volume	1924.6(3) $Å^3$	
Z	2	
Density (calculated)	1.213 Mg/m^3	
Absorption coefficient	0.075 mm^{-1}	
F(000)	760	
Crystal size	0.31 x 0.15 x 0.11 mm^3	
Theta range for data collection	2.157 to 27.640°.	
Index ranges	-13<=h<=13, -15<=k<=16, -18<=l<=19	
Reflections collected	26322	
Independent reflections	4419 [R(int) = 0.0895]	
Completeness to theta = 25.242°	99.9 %	
Absorption correction	Semi-empirical from equivalents	
Max. and min. transmission	1.00 and 0.770	
Refinement method	Full-matrix least-squares on F^2	
Data / restraints / parameters	4419 / 2 / 266	
Goodness-of-fit on F^2	1.210	
Final R indices [I>2sigma(I)]	R1 = 0.0865, wR2 = 0.1755	
R indices (all data)	R1 = 0.1272, wR2 = 0.1871	
Extinction coefficient	n/a	
Largest diff. peak and hole	0.441 and -0.293 e.$Å^{-3}$	
CCDC #	1583699	

Supplementary Table 7 Crystal data and structure refinement for **9**.

Supplementary References

1. Sokkalingam, P., Kim, D. S., Hwang, H., Sessler, J. L. & Lee, C.-H. A dicationic calix[4]pyrrole derivative and its use for the selective recognition and displacement-based sensing of pyrophosphate. *Chem. Sci.* **3,** 1819–1824 (2012).
2. Kim. D. S., Lynch, V. M., Park, J. S. & Sessler, J. L. Three distinct equilibrium states via self-assembly: simple access to a supramolecular ion-controlled NAND logic gate. *J. Am. Chem. Soc.* **135,** 14889–14894 (2013).
3. Shimada, H. *et al*. Comparative inhibition of tetrameric carbonyl reductase activety in pig heart cytosol by alkyl 4-pyridyl ketones. *J. Enzyme Inhib. Med. Chem.* **29,** 397–400 (2014).
4. Gans, P., Sabatini, A. & Vacca, A. Investigation of equilibria in solution. Determination of equilibrium constants with the HYPERQUAD suite of programs. *Talanta* **43,** 1739–1753 (1996).
5. Rigaku Americas Corportion. CrystalClear 1.40, The Woodlands, TX. (2008).
6. Altomare, A. *et al*. SIR97. A program for crystal structure solution. *J. Appl. Cryst.* **32,** 115–119 (1999).
7. Sheldrick, G. M. Program for the refinement of crystal structures. *Acta Cryst.* **A64,** 112–122 (2008).
8. Spek, A. L. PLATON. A multipurpose crystallographic tool. Utrecht University, The Netherlands (1998).
9. Farrugia, L. J. WinGX 1.64. An integrated system of windows programs for the solution, refinement and analysis of single crystal X-ray diffraction data. *J. Appl. Cryst.* **32,** 837–838 (1999).
10. A. J. C. Wilson. Tables for X-ray Crystallography Vol. C, Tables 4.2.6.8 and 6.1.1.4, Kluwer Academic Press International (1992).
11. Sheldrick, G. M. SHELXTL/PC (Version 5.03). Siemens Analytical X-ray Instruments, Inc., Madison, Wisconsin, USA (1994).
12. SAINT V8.27B Bruker AXS Inc, Madison, WI (2012).
13. Palatinus, L. & Chapuis, G. Superflip – A computer program for the solution of crystal structures by charge flipping in arbitrary dimensions. *J. Appl. Cryst.* **40,** 786–790 (2007).
14. CrysAlisPro. Agilent Technologies Agilent Technologies UK Ltd., Oxford, UK, SuperNova CCD System, CrysAlicPro Software System, 1.171.37.31 (2013).
15. *van der* Sluis, P. & Spek, A. L. SQUEEZE. *Acta Cryst.* **A46,** 194–201 (1990).
