# Supplementary material for: Control over multiple molecular states with directional changes driven by molecular recognition
Source: Nat Commun. 2018 Feb 26;9:823. doi: 10.1038/s41467-018-03220-0 (PMC5827562; doi:10.1038/s41467-018-03220-0)
Supplement: Supplementary file 2 — Peer Review File [file 41467_2018_3220_MOESM2_ESM.pdf]

Editorial Note: This manuscript has been previously reviewed at another journal that is not operating a transparent peer review scheme. This document only contains reviewer comments and rebuttal letters for versions considered at Nature Communications.

REVIEWERS' COMMENTS:

Reviewer #4 (Remarks to the Author):

I reviewed the previous version of this manuscript, and the authors have fully addressed my and other reviewers' comments. Hence, it is recommended for publication in Nature Comm. as such.
